# Recommendations for clinical management of excessive daytime sleepiness in obstructive sleep apnoea – A Delphi consensus study

**DOI:** 10.1016/j.sleep.2023.10.001

**Published:** 2023-10-09

**Authors:** Joerg S. Steier, Richard K. Bogan, Irene M. Cano-Pumarega, John A. Fleetham, Giuseppe Insalaco, Chitra Lal, Jean-Louis Pépin, Winfried J. Randerath, Susan Redline, Atul Malhotra

**Affiliations:** a Centre for Human and Applied Physiological Sciences, Faculty of Life Sciences and Medicine, King’s College London, London, UK; b Medical University of South Carolina, Charleston, SC, USA; c Sleep Unit, Respiratory Department, Ramón y Cajal University Hospital, IRYCIS, CIBERES, Madrid, Spain; d Department of Medicine, University of British Columbia, Vancouver, BC, Canada; e Institute of Translational Pharmacology, Italian National Research Council, Palermo, Italy; f Pulmonary, Critical Care, Allergy and Sleep Medicine, Medical University of South Carolina, College of Medicine, Charleston, SC, USA; g Grenoble Alpes University, INSERM, University Hospital Grenoble Alpes, HP2, Grenoble, France; h Institute of Pneumology at the University of Cologne, Bethanien Hospital, Clinic for Pneumology and Allergology, Centre of Sleep Medicine and Respiratory Care, Solingen, Germany; i Brigham and Women’s Hospital, Harvard Medical School, Boston, MA, USA; j University of California, San Diego Health, La Jolla, CA, USA

**Keywords:** Sleep apnoea syndromes, Practice guideline, Expert testimony

## Abstract

**Study objective:**

Excessive daytime sleepiness is common with obstructive sleep apnoea and can persist despite efforts to optimise primary airway therapy. The literature lacks recommendations regarding differential diagnosis and management of excessive daytime sleepiness in obstructive sleep apnoea. This study sought to develop expert consensus statements to bridge the gap between existing literature/guidelines and clinical practice.

**Methods:**

A panel of 10 international experts was convened to undertake a modified Delphi process. Statements were developed based on available evidence identified through a scoping literature review, and expert opinion. Consensus was achieved through 3 rounds of iterative, blinded survey voting and revision to statements until a predetermined level of agreement was met (≥80 % voting “strongly agree” or “agree with reservation”).

**Results:**

Consensus was achieved for 32 final statements. The panel agreed excessive daytime sleepiness is a patient-reported symptom. The importance of subjective/objective evaluation of excessive daytime sleepiness in the initial evaluation and serial management of obstructive sleep apnoea was recognised. The differential diagnosis of residual excessive daytime sleepiness in obstructive sleep apnoea was discussed. Optimizing airway therapy (eg, troubleshooting issues affecting effectiveness) was addressed. The panel recognised occurrence of residual excessive daytime sleepiness in obstructive sleep apnoea despite optimal airway therapy and the need to evaluate patients for underlying causes.

**Conclusions:**

Excessive daytime sleepiness in patients with obstructive sleep apnoea is a public health issue requiring increased awareness, recognition, and attention. Implementation of these statements may improve patient care, long-term management, and clinical outcomes in patients with obstructive sleep apnoea.

## 1. Introduction

Recent estimates suggest that nearly one billion adults worldwide have obstructive sleep apnoea (OSA) [1]. Excessive daytime sleepiness (EDS) is the cardinal symptom of OSA [2]. Some studies estimate that 55%–80% of patients with OSA report EDS prior to initiating therapy [3–5], although this may vary depending on the setting and definition [6]. EDS persists in some patients despite normalisation of breathing, oxygenation, and sleep quality when treated with primary OSA therapy, such as continuous positive airway pressure (CPAP) [7,8]. Population-based studies have estimated that between 10% and 28% of patients treated with CPAP continue to experience residual EDS, even when other potential causes of sleepiness (eg, depression, other sleep disorders, medications, comorbidities, and inadequate sleep duration) are controlled [5,9,10]. Recent publications have provided general reviews on EDS in OSA [11,12]; however, there remains a need for consensus in practical guidance on the differential diagnosis and management of EDS in patients with OSA.

The Delphi method is a well-established process for achieving consensus from multiple stakeholders that has been used in healthcare for many purposes [13,14]. It is often employed as a means of eliciting expert opinion on topics for which there is an absence of definitive evidence to guide decisions. Experts in the field participate in rounds of anonymous, iterative survey voting, during which statements/guidelines are voted on and revised accordingly until consensus is achieved [13,14]. In a conventional Delphi method, the survey process is conducted electronically [14].

The current study was designed to address key questions and controversies related to the differential diagnosis and management of EDS in patients with OSA. A Delphi approach was used to develop a set of expert consensus statements to bridge the gap between existing literature/guidelines and real-world practice by providing practical guidance necessary to support clinical decision-making.

## 2. Methods

### 2.1. Participants

The Delphi panel included ten international experts in sleep and respiratory medicine from North America and Europe. Two panellists served as co-chairs (J.S. and A.M.) and eight served as section leads (R. B., I.C.-P., J.F., G.I., C.L., J.-L.P., W.R., S.R.). Panellists were invited by the co-chairs based on expertise within the field, diversity in demographics and geography, and background in respiratory and sleep disorders extending beyond sleep apnoea, as well as experience in conducting clinical trials, including those involving pharmacotherapy. Panellists included experts who were clinically active at tertiary referral centres with academic input. Per the protocol, invited panellists who were unable to commit to all rounds of the Delphi process were to be excluded; all invited panellists were included in this study. The study protocol was registered through King’s College London via the study co- chair, J.S. Jazz Pharmaceuticals financially sponsored this study. Financial support for this manuscript was provided by Jazz Pharmaceuticals and Axsome Therapeutics. The co-chairs and faculty panel led all steps involved in the planning, evidence review, survey process, and the statement development, which were conducted independently of the industry sponsor.

### 2.2. Modified Delphi process

This study utilised a modified Delphi method, which differs from a conventional Delphi method such that part of the survey process (eg, final voting) is conducted during a live meeting [14]. The modified Delphi process involved two parts: scoping literature review and survey voting (Supplementary Fig. 1). Co-chairs developed questions related to the differential diagnosis and management of EDS in OSA, which were grouped into four topics (two section leads [one from North America and one from Europe] per topic) (Supplementary Table 1). These questions were used to guide the literature review and generation of statements. The protocol was registered on ClinicalTrials.gov (NCT05055271).

#### 2.2.1. Scoping literature review

To ensure the initial statements were guided by existing evidence, a scoping literature review (English language, no limitation on year) was conducted by the study coordinator under the direction of section leads (who determined search parameters, queries, and selection criteria) (see Supplementary Tables 2 and 3 in the online data supplement). Section leads developed initial statements that reflected available evidence and their expert opinion based on experience in/knowledge of the clinical management of EDS in OSA.

#### 2.2.2. Survey voting

An anonymous online survey was developed (compiled by the study coordinator and administered via SurveyMonkey [Momentive, San Mateo, CA]) to determine the panel’s level of agreement with the initial statements. The survey was administered to the full panel for each round of remote voting. The first 2 rounds of voting were conducted remotely, while the final round was conducted at a live virtual meeting. All votes and responses for all rounds of voting (remote and live/virtual) were blinded to all panellists. Level of agreement was rated on a five-point Likert scale: strongly agree (A+), agree with reservation (A), undecided (U), disagree (D), or strongly disagree (D+). The survey also included a field for open-ended feedback.

Upon receipt of the first round of responses, section leads revised the statements to achieve further agreement within the panel. At the discretion of the section leads, statements could be regrouped, combined, and/or new statements could be generated to address the feedback. Updated statements were compiled into a new survey and redistributed for a second round of remote voting and subsequent revisions.

Final round of voting was conducted at a two-day live virtual meeting (hosted on Zoom [San Jose, CA]; moderated by co-chairs) to allow the panel to discuss the rationale for the statements, how the statements had evolved, and any remaining reservations for each statement. Statements were further revised at the live meeting based on the discussion, and consensus was determined via anonymous voting (Poll Everywhere, San Francisco, CA). Additional voting and revision continued until consensus was achieved or the statement was removed.

The primary outcome measure was the level of agreement from the panel for all statements. Consensus was predefined as ≥80% of the panel rating a statement A+ or A.

## 3. Results

### 3.1. Rationale for recommendations

The recommendation statements were based on the Delphi consensus process, supported by existing data from selected literature (see Supplementary Tables 4–12 in the online data supplement) and guided by expert opinion based on experience and knowledge of the clinical management of EDS in OSA. All panellists participated in all review rounds.

### 3.2. Summary of key evidence and recommendations

The panel’s agreement on the statements gradually increased across review rounds as the statements were revised based on iterative survey voting and feedback from each member (Supplementary Fig. 2). At the completion of final voting, consensus was achieved for 32 final statements.

#### 3.2.1. Topic 1: how to define and evaluate EDS

Consensus was achieved for all final statements for Topic 1 (Table 1). EDS is defined by difficulty maintaining alertness during the wake periods of a 24-h sleep-wake cycle. Given the prevalence and importance of EDS in the management of patients with OSA, the panel recommended that EDS be assessed during the initial evaluation of OSA and serially over time (at six weeks to three months following initiation of therapeutic intervention). Thereafter, patients should be reassessed using an appropriate and validated instrument on at least an annual basis to ensure optimal management. Various instruments for assessing EDS were identified, including questionnaires (eg, the Epworth Sleepiness Scale [ESS]) and objective tests (eg, Multiple Sleep Latency Test [MSLT], Maintenance of Wakefulness Test [MWT]; Table 2). Although one specific instrument was not recommended over another, ESS is the most commonly used questionnaire to assess EDS, with changes of two to three points or a ≥25% reduction from baseline in ESS score indicating a minimum clinically important difference [15–17]. The ESS is, however, limited by its poor predictive value for neurocognitive outcomes and/or motor vehicle accidents and variable test-retest and construct validity, with specific concerns regarding poor psychometric performance in certain populations (eg, older individuals) [18,19]. Recent data have shown that the ESS has good test-retest reliability in controlled clinical trial settings [20] and less reliability when administered across different healthcare settings (eg, between a primary care visit and sleep specialist/secondary care visit) [19,21]. The Patient-Reported Outcomes Measurement Information System (PROMIS) Sleep Related Impairment scale (SRI), based on item response theory, has been increasingly used in research settings and holds promise for clinical use, but requires further evaluation for use in management of OSA [22]. The ability of the PROMIS SRI to gauge the severity of sleep-wake problems on a continuum could be particularly useful to enable patients to capture real-time feedback on sleep disturbances and EDS. Most of the alternative questionnaires have not been fully evaluated in terms of their predictive value for important health outcomes (eg, occupational accidents) or psychometric properties (Table 2) [18,19,23–33]. Objective testing of the level of sleepiness with the MSLT or MWT can be useful for quantifying EDS and diagnosing specific conditions (eg, narcolepsy), but these tests require controlled and time-consuming laboratory procedures, precluding widespread and repeated use. The interpretation of any subjective or objective tests for EDS should be undertaken in the appropriate clinical context, with particular attention to the risks of EDS in high-risk individuals (eg, commercial drivers).

#### 3.2.2. Topic 2: how to define and evaluate residual EDS in OSA in patients treated with primary OSA therapy

Consensus was achieved for all final statements for Topic 2 (Table 3). If a patient treated for OSA is suspected of having residual EDS, it is important to first ensure that EDS is not due to suboptimal treatment of OSA, including inadequate adherence to primary OSA therapy. Studies have shown that three months of CPAP therapy can result in resolution of EDS in 66% of patients who are sleepy at baseline [34], and longer durations of treatment (beyond three to six months) may produce further improvement in EDS [5]. Thus, the panel recommended that EDS should be reassessed after three to six months of optimal CPAP therapy, although this window can be individualised. The impact of EDS on alertness, fatigue, cognitive function, and mood should also be recognised.

While the optimal duration of nightly CPAP usage varies, depending on the outcome studied, longer duration of CPAP use per night is beneficial [34]. CPAP usage of more than 6 h per night has been shown to improve several long-term outcomes, including survival [35] and cognition [36], hence the panel recommended that CPAP be used for more than 6 h per 24-h period. In sum, the panel recommended that a diagnosis of residual EDS should only be considered after six months of optimal (≥6 h/night) CPAP therapy.

If a patient continues to experience EDS after primary OSA therapy has been optimised, the clinician should evaluate for other factors that may be contributing to EDS, such as insufficient sleep, lifestyle factors (eg, diet and exercise), overlapping sleep disorders (eg, narcolepsy and idiopathic hypersomnia), comorbidities (eg, hypothyroidism), concomitant medications, and illicit drug use [37,38]. Long-term management and decision making regarding EDS should include discussion of these factors. Sleep durations of less than 7 h per night can increase the risk of adverse health sequelae, such as cardiovascular disease and diabetes [39]. Sleeping more than 9 h per night is also associated with an increased risk for adverse health outcomes. Hence, the panel recommended that an optimal sleep duration for healthy adults is seven to 9 h per night. This is consistent with recommendations from the National Sleep Foundation and the American Thoracic Society [40,41]. While good sleep hygiene is of paramount importance to ensure optimal nocturnal sleep, short naps can help to achieve an adequate amount of sleep in a 24-h day [42]. Studies have used questionnaires, sleep diaries, and actigraphy to quantify sleep duration; however, the panel noted there are no strong data to recommend one method over another.

#### 3.2.3. Topic 3: how to address specific clinical challenges related to residual EDS in OSA

Consensus was achieved for all final statements for Topic 3 (Table 4). Some patients with residual EDS in OSA struggle with CPAP therapy because of challenges with achieving high adherence (eg, due to nasal congestion, pressure intolerance, side effects, mask leaks, residual events). Remote monitoring of CPAP downloads can provide valuable information when attempting to address problems related to adherence. In cases related to pressure intolerance, in-laboratory pressure titration can be helpful to optimise pressure settings and, when necessary, provide alternative positive airway pressure modalities (eg, bi-level ventilation). Educational and behavioural strategies (eg, intensive support), technological tools (eg, patient engagement approaches, telemedicine), and pharmacotherapy (eg, drugs to address insomnia or anxiety) are other helpful techniques to facilitate CPAP adherence [43,44]. If a patient is unable or unwilling to continue with CPAP therapy or if CPAP does not achieve adequate results, alternative therapies for OSA should be considered (eg, oral appliance, positional therapy, hypoglossal nerve stimulation, and upper airway or bariatric surgery in selected cases) [45].

If the patient continues to experience EDS after treatment of underlying OSA has been maximized, then additional diagnostic testing (eg, sleep diaries, actigraphy, MSLT) might be helpful in assessing other potential causes of EDS. Diagnostic criteria for idiopathic hypersomnia (IH) includes careful consideration and *exclusion* of other disorders that may cause sleepiness [46]. Considering this definition and from a semantic point of view, concomitant OSA and IH can exist if a patient has previously been diagnosed with IH (at which time a diagnosis of OSA was appropriately excluded), and later develops OSA. If a patient is diagnosed with OSA and then presents with EDS, IH should not be considered as an additional diagnosis.

#### 3.2.4. Topic 4: when and how pharmacological treatment for EDS in OSA should be initiated

Consensus was achieved for all final statements for Topic 4 (Table 5). The panel reinforced the notion that there are some patients who have residual EDS despite adequate primary therapy for OSA. In general, wake-promoting agents should be considered for residual EDS in OSA following optimisation of OSA therapy. However, there may also be some patients who are partially or incompletely treated (such as patients who are poorly adherent with OSA therapy or those in whom the residual apnoea-hypopnoea index [AHI] of *<*5/hour cannot be achieved). For such patients, pharmacotherapy may be beneficial and could be considered on an individual basis by an experienced sleep clinician. The panel acknowledged that there are limited data to support this recommendation [47,48]; however, this recommendation was prompted by the significant adverse consequences of EDS (such as higher risk of motor vehicle and occupational accidents, poor quality of life, and cognitive impairment) and favourable safety profiles of newer wake-promoting agents. The panel stressed that all efforts to optimise the delivery of treatments aimed to improve airway patency should have been made and other causes of EDS identified and addressed before initiating pharmacotherapy. There are limited data regarding the efficacy and safety of pharmacotherapy in untreated OSA and, therefore, the long-term outcomes of pharmacotherapy in those patients are not conclusively clarified. Individuals who might be amenable to pharmacological intervention need to be carefully evaluated for other underlying causes of EDS and should be closely monitored on treatment.

The majority of studies identified in the literature review (see Supplementary Table 12 in the online data supplement) included patients with EDS despite optimal CPAP use and/or patients who were nonadherent to CPAP therapy despite intensive efforts. The studies excluded patients with concurrent sleep, general medical, neurological, or psychiatric disorders that could cause sleepiness. There is no evidence that wake-promoting drugs influence the underlying pathophysiology of the concomitant sleep disorders. However, short- and long-term use (eg, 12 weeks and 1 year, respectively) of wake-promoting agents have been shown to improve residual EDS in OSA, often accompanied by an improved quality of life [49–67]. Data also show that wake-promoting agents are associated with improvement in validated self-assessment questionnaires and objective outcome measures, such as vigilance tests or electrophysiological tests. Pharmacotherapy may also be beneficial for patients with symptoms at work (eg, drivers) when close monitoring and supervision are provided. The available wake-promoting drugs may differ in terms of adverse effects (eg, changes in blood pressure, changes in heartrate, headache, etc), which might influence the selection of pharmacotherapy for individual patients.

## 4. Discussion

The synthesis of the literature and the consensus of the Delphi panel generated 32 statements on the definition and evaluation of residual EDS in OSA, how to address specific challenges in its management, and when and how to consider and initiate pharmacotherapy (Table 1, 3–5). EDS is a patient-reported symptom and the importance of subjective and objective evaluation of EDS in the initial evaluation and the serial management of patients was recognised. Optimizing primary OSA airway therapy, including troubleshooting potential issues influencing its efficacy, remains an essential component of the therapeutic approach; algorithms for symptom management have been reported elsewhere [68]. Clinicians need to consider the differential diagnoses of residual EDS in OSA and the need to evaluate patients for underlying causes that are not related to OSA. Finally, the panel recognised the occurrence of residual EDS in OSA despite optimised primary therapy and recommended the consideration of adjunct pharmacotherapy in its management. The use of wake-promoting agents to treat EDS in specific cases is increasingly recognised based on data from clinical trials, although there remains a need for further long-term data on safety and efficacy [52].

There were several limitations of this Delphi consensus. Because of the limitations of the published evidence, some of the recommendations provided by the panel extend beyond the existing literature and are also based on expert opinion from clinical experience, which must be corroborated by future research. In addition, the number of panellists was relatively small and only comprised experts from North America and Europe; thus geographic representation was limited to these regions and potential variations in clinical management that may be applicable to other areas of the world may not be accounted for in these recommendations. Further, the sociocultural context of EDS was discussed based on the available literature, which is limited by the lack of data for large portions of the world’s population. Prior to submission, a respiratory research and patient advocate reviewed the manuscript and statements; however, direct insight from patients with OSA was not included as part of this study. Additionally, a scoping review, rather than a traditional systematic review, was conducted on English-language publications only, although the authors were not aware of major overlooked publications with validated questionnaires from other languages. Of note, the final recommendation statements have not yet been validated by a larger group of healthcare providers. Future work is needed to assess the feasibility of implementing these guidelines in various clinical practice settings.

## 5. Conclusions

There was general agreement among the Delphi panel that residual EDS in OSA is a major public health problem that requires increased awareness, recognition, and attention. The tools available for evaluating EDS are limited and need to be interpreted in the appropriate clinical context. Residual EDS should be addressed and pharmacological intervention considered once CPAP or other primary therapy for OSA has been optimised. Encouraging engagement and support from family and friends may help facilitate patient adherence with primary treatment, thereby improving management. The panel also recommended that in selected patients with partially treated or incompletely treated OSA, pharmacotherapy may be beneficial and could be considered on an individual basis and prescribed by an experienced sleep clinician with close clinical follow-up. Implementation of the consensus recommendations will help to improve patient care, long-term management, and clinical outcomes of patients with OSA.

## Supplementary Material

sup

## Figures and Tables

**Table 1:** Level of agreement/disagreement with each statement during each round:^a^ How to define and evaluate EDS.

Statement Number	Statement^b^	Response	Response Rate (%)		
			Round 1	Round 2	Round 3
1	Sleepiness should be assessed at baseline and then re-assessed within 6 weeks to 3 months, and then annually thereafter, following initiation of therapy for OSA. In well-treated patients without clinical need, less frequent assessments may also be acceptable. More frequent assessments may be needed in cases with patient-initiated contact, severity and clinical impact of EDS, reasons for concern, changes in health status (eg, weight gain), or other considerations (eg, high-risk occupations, such as professional drivers or those operating machinery)	Strongly agree Agree with reservation Undecided Disagree Strongly disagree	60	100	100
			40	0	0
			0	0	0
			0	0	0
			0	0	0
2	Sleepiness and EDS are patient-reported symptoms that can be defined by the inability to perform/master tasks that require vigilance or to stay awake against intention. Assessment of the symptom in a real-world population requires the use of a careful history, supplemented by standardized questionnaires that quantify the degree of EDS and related impairment. In the context of evaluating the spectrum of sleep disorders, the assessment maybe complemented by a clinician-guided choice of objective markers (eg, Maintenance of Wakefulness Test, Multiple Sleep Latency Test, Psychomotor Vigilance Task)	Strongly agree Agree with reservation Undecided Disagree Strongly disagree	80	80	100
			20	20	0
			0	0	0
			0	0	0
			0	0	0
3	The Epworth Sleepiness Scale (ESS) is the most commonly used questionnaire to assess EDS in adult patients with OSA within a clinical setting; however, alternative questionnaires (eg, Patient Reported Outcomes Measurement System Sleep-Related Impairment [PROMIS SRI]) may be appropriate. Additional evaluation using objective measures may be more appropriate for certain patient populations (eg, where questionnaire items may be unsuitable for different cultures or populations) and in those where there are discrepancies between questionnaire-based assessments and clinical history	Strongly agree Agree with reservation Undecided Disagree Strongly disagree	50	60	100
			50	30	0
			0	0	0
			0	10	0
			0	0	0
4	Clinicians are encouraged to understand the limitations associated with each instrument and use psychometric characteristics to choose the most appropriate tool for a specific population and context. Table 2 lists instruments used to assess sleepiness and related outcomes in patients with OSA and their psychometric properties, including sensitivity, specificity, reliability (internal consistency), repeatability (test-re-test reliability), construct validity, and clinical relevance.	Strongly agree Agree with reservation Undecided Disagree Strongly disagree	80	90	100
			20	0	0
			0	10	0
			0	0	0
			0	0	0
5	Future research is needed to validate and generate additional psychometric data on promising instruments to measure sleepiness and other patient-reported outcomes, with particular attention to certain populations (eg, depending on age, sociocultural background, language, special needs, and other factors).	Strongly agree Agree with reservation Undecided Disagree Strongly disagree	80	90	100
			20	10	0
			0	0	0
			0	0	0
			0	0	0
6	Although EDS occurs across a continuum, cut-off values are useful for defining clinically important levels of EDS (Table 2) but should not be used in isolation. These values can be used in the appropriate clinical context to identify patients who may benefit from additional intervention.	Strongly agree Agree with reservation Undecided Disagree Strongly disagree	50	80	100
			50	10	0
			0	0	0
			0	10	0
			0	0	0
7	Clinicians should consider objective assessments in appropriate clinical contexts when caring for patients with EDS where results may change management (eg, discordance between patient report and clinician or partner perception).	Strongly agree Agree with reservation Undecided Disagree Strongly disagree	80	90	100
			10	10	0
			10	0	0
			0	0	0
			0	0	0

a Consensus was considered to be achieved if ≥ 80% of respondents agreed or agreed with reservation.

b Final statements shown. CPAP = continuous positive airway pressure, EDS = excessive daytime sleepiness, ESS = Epworth Sleepiness Scale, IH = idiopathic hypersomnia, OA = oral appliance, OSA = obstructive sleep apnoea, PAP = positive airway pressure, PROMIS SRI=Patient-Reported Outcomes Measurement Information System Sleep-Related Impairment, QoL = quality of life.

**Table 2:** Instruments used to assess sleepiness and related outcomes in patients with OSA and their psychometric properties.

Tool	Sensitivity	Specificity	Reliability (Internal Consistency)	Repeatability (Test-retest Reliability)	Construct Validity	MCID Threshold
Epworth Sleepiness Scale (ESS)	93⋅5% (for cutoff score *>*10) [23]	100% (for cutoff score *>*10) [23]	Cronbach’s α = 0⋅70–0⋅88 (acceptable) [18] Not unidimensional [18]	Recent data have questioned the short-term and long-term reliability of the ESS with insufficient test-retest reliability even within the same day, suggesting situational sleepiness (ie, time of test administration) may influence ESS scores [19,24] Studies have found the ESS to have good test-retest reliability in controlled clinical trials settings and less reliability when administered across different primary/secondary care settings (eg, between PCP and sleep specialist visit) [19, 24]	Moderate association between ESS and MWT [18] Weak association between ESS and MSLT [18] ESS shows statistical, but perhaps not clinically meaningful, differences across groups with known differences in EDS [18]	Decrease of 2–3 points, although some suggest a decrease of 5–6 points be considered as the MCID for clinical decision-making or when assessing treatment effects to account for low reliability [24,25]
Patient-Reported Outcomes Measurement Information System Sleep-Related Impairment (PROMIS SRI)	NA^a^	NA^a^	Cronbach’s α = 0.96 (high) [26]	NA^a^	PROMIS shows statistical differences between individuals with and without a sleep disorder, as well as between those with treated and untreated sleep disorders [26]	≥2 points^b^ [27]
Multiple Sleep Latency Test (MSLT)	80⋅9% (for cutoff <5 min) [23] 94⋅5% (for cutoff *<*8 min)^c^ [23] Low sensitivity for severe levels of EDS	89⋅8% (for cutoff *<*5 min) [23] 73⋅3% (for cutoff *<*8 min)^c^ [23]	Can be sensitive to a basement effect, as well as affected by activity prior to testing, caffeine use, age, and instructions [23]	High test-retest reliability in healthy individuals [23,28] Excellent intra- and interrater reliability in sleep disorder populations, although variable depending on sleep disorder (test-retest correlation r = 0⋅65 for insomnia and r = 0⋅93 for narcolepsy) [23,28]	MSLT mean sleep latency is characteristically increased after administration of stimulant medication and reduced after administration of sedating medication [23]	There are no established levels to indicate what magnitude of change is considered clinically important [28]
Maintenance of Wakefulness Test (MWT)	84% (for cutoff *<*12 min)^‡^ [23]	98% (for cutoff *<*12 min)^c^ [23]	Can be sensitive to a ceiling effect, as well as affected by activity prior to testing, caffeine use, age, and instructions [23]	NA	MWT mean sleep latency is increased after the administration of wake- promoting medication,^a^ stimulants, and CPAP treatment and is reduced after the administration of sedating medication [28]	There are no established levels to indicate what magnitude of change is considered clinically important [28]
Functional Outcomes of Sleep Questionnaire (FOSQ)	AHI ≥5 = 32⋅8% [29] AHI ≥15 = 34⋅4% [29] AHI ≥30 = 33⋅3% [29]	AHI ≥5 = 66⋅7% [29] AHI ≥15 = 77⋅8% [29] AHI ≥30 = 67⋅9% [29] NA	Cronbach’s α = 0⋅87 [30]	Test-retest correlation of Total score was r = 0⋅90; test-retest reliability of individual subscales ranges from r = 0⋅81 to r = 0⋅90 [31]	Acceptable as determined by correlation of FOSQ and SAQLI with ESS [32]	Change of 1 SEM [32]
Sleep Apnea Quality of Life Index (SAQLI)	May be insensitive to important changes in QoL that patients with OSA experience as a result of therapy [33]	NA	Cronbach’s α ≥ 0⋅90 [33]	Test-retest correlation ranges from r = 0⋅91 to r = 0⋅94 [33]	Moderate to high correlation between SAQLI and related measures on related questionnaires (symptoms, daily life activities, social interactions, and emotions on the SF-36, ESS, Beck Depression Inventory, and Symptom Checklist 90) [33]	Change of 1 SEM [32]

AHI = apnoea-hypopnoea index, EDS = excessive daytime sleepiness, ESS = Epworth Sleepiness Scale, FOSQ=Functional Outcomes of Sleep Questionnaire, MCID = minimum clinically important difference, MSLT = Multiple Sleep Latency Test, MWT = Maintenance of Wakefulness Test, NA = not applicable, OSA = obstructive sleep apnoea, PCP = primary care physician, PROMIS SRI=Patient-Reported Outcomes Measurement Information System Sleep-Related Impairment, QoL = quality of life, SAQLI=Sleep Apnea Quality of Life Index, SEM = standard error of mean, SF-36 = 36-Item Short Form Health Survey.

a NA for adult OSA population.

b Not a sleep disorder population.

c In patients with narcolepsy.

**Table 3:** Level of agreement/disagreement during each round:^a^ How to define and evaluate residual EDS in OSA in patients treated with primary OSA therapy.

Statement Number	Statement^b^	Response	Response Rate (%)		
			Round 1	Round 2	Round 3
8	Before diagnosing residual EDS associated with OSA, the patient should use optimal primary airway therapy (eg, CPAP, oral appliance) for 3–6 months, although the clinician should use his/her clinical judgment as longer treatment periods may be necessary to observe improvements in some patients. The clinician should first assess whether OSA treatment is optimised based on clinical assessment, CPAP downloads, and CPAP titration as necessary. If the patient is on CPAP treatment, clinicians should assess for good compliance (used throughout sleep, ideally for at least 6 h per night) and for correct mask fitting with limited leaks should be ensured; if the patient is treated with OA, appropriate adjustment to minimize residual sleep-disordered breathing and a good compliance should be assessed.	Strongly agree Agree with reservation Undecided Disagree Strongly disagree	40	40	90
			50	40	10
			0	0	0
			10	20	0
			0	0	0
9	For optimal treatment of OSA, it is recommended that CPAP is used throughout sleep, ideally for at least 6h per night, although longer durations of use may be needed to observe improvement in some outcomes.	Strongly agree Agree with reservation Undecided Disagree Strongly disagree	70	90	90
			20	10	10
			10	0	0
			0	0	0
			0	0	0
10	Prior to diagnosing residual EDS associated with OSA, other causes should be evaluated, including inadequate sleep duration, drug abuse (eg, tetrahydrocannabinol), comorbidities (eg, depression, hypothyroidism), concomitant medications, and other sleep disorders (eg, idiopathic hypersomnia, narcolepsy). A differential diagnosis can often be made by the primary care physician based on medical history, but referral to a sleep specialist may be warranted for more complex cases.	Strongly agree Agree with reservation Undecided Disagree Strongly disagree	70	100	90
			30	0	10
			0	0	0
			0	0	0
			0	0	0
11	Adequate self-reported sleep duration in adults is optimally 7–9 h per 24-h period, however, there may be a wide range of what is considered normal. Sleep durations ≤6 h per 24-h period are associated with documented adverse health sequelae, such as impaired immunity, cardiometabolic disorders and increased risk of mortality. Although there is some individual variability, sleep durations greater than 9h per 24-h period, can be associated with adverse health-related sequelae, and may reflect underlying sleep disorders or comorbidities.	Strongly agree Agree with reservation Undecided Disagree Strongly disagree	70	80	90
			30	20	10
			0	0	0
			0	0	0
			0	0	0
12	There is no gold standard for assessment of sleep duration. To establish whether the above criterion for adequate sleep has been met for an individual patient, clinicians may consider assessment of sleep duration using sleep diaries over a 2-week period, actigraphy, or other validated wearable technologies.	Strongly agree Agree with reservation Undecided Disagree Strongly disagree	90	70	80
			0	10	20
			0	10	0
			10	10	0
			0	0	0
13	In patients with EDS and chronic partial sleep deprivation (less than 7 h per 24-h period), it is recommended to encourage healthy sleep and circadian practices and consider short naps as an initial approach to ensure an adequate amount of sleep is obtained over a 24-h period.	Strongly agree Agree with reservation Undecided Disagree Strongly disagree	60	70	100
			30	20	0
			0	10	0
			10	0	0
			0	0	0
14	Some individuals have residual EDS, even after optimizing primary airway therapy and in the absence of other identified causes, although the mechanisms are unclear.	Strongly agree Agree with reservation Undecided Disagree Strongly disagree	90	100	100
			10	0	0
			0	0	0
			0	0	0
			0	0	0

a Consensus was considered to be achieved if ≥ 80% of respondents agreed or agreed with reservation.

b Final statements shown. CPAP = continuous positive airway pressure, EDS = excessive daytime sleepiness, ESS = Epworth Sleepiness Scale, IH = idiopathic hypersomnia, OA = oral appliance, OSA = obstructive sleep apnoea, PAP = positive airway pressure, PROMIS SRI=Patient-Reported Outcomes Measurement Information System Sleep-Related Impairment, QoL = quality of life.

**Table 4:** Level of agreement/disagreement with each statement during each round:^a^ How to address specific clinical challenges related to residual EDS in OSA.

Statement Number	Statement^b^	Response	Response Rate (%)		
			Round 1	Round 2	Round 3
15	In patients with EDS who are intolerant to PAP therapy (or other primary therapies) or have attempted/are attempting therapy, clinicians should identify and attempt to resolve all issues related to therapy intolerance and consider appropriate alternative therapies.	Strongly agree Agree with reservation Undecided Disagree Strongly disagree	80	100	100
			20	0	0
			0	0	0
			0	0	0
			0	0	0
16	Optimizing primary therapy for OSA to improve adherence and efficacy is important and could include educational and behavioral strategies, technological tools (telemedicine), and pharmacological treatments (eg, for insomnia, anxiety).	Strongly agree Agree with reservation Undecided Disagree Strongly disagree	70	100	100
			20	0	0
			10	0	0
			0	0	0
			0	0	0
17	Main strategies to improve adherence are to: a) provide intensive educational support, patient engagement tools, cognitive behavioral therapy, and/or mask desensitization; b) check the mask fit for optimal comfort and leaks; c) address nasal patency; d) identify and treat any side effects, including consideration of a chin strap or humidification; e) assess the correct pressure titration and consider additional PAP modalities; f) assess and treat any comorbid sleep disorders (eg, insomnia) and consider use of sedative-hypnotic treatment during CPAP initiation; g) problem solve any barriers including psychosocial, financial, and/or motivational.	Strongly agree Agree with reservation Undecided Disagree Strongly disagree	60	100	100
			30	0	0
			0	0	0
			10	0	0
			0	0	0
18	For patients who refuse PAP or remain non-adherent despite the previous strategies, clinicians should prioritize lifestyle interventions and consider the use of alternative therapies including oral appliances, positional therapy, surgery (including hypoglossal nerve stimulation, adenotonsillectomy, maxillomandibular osteotomy, bariatric surgery), and/or upper airway neuromuscular intervention.	Strongly agree Agree with reservation Undecided Disagree Strongly disagree	60	100	100
			40	0	0
			0	0	0
			0	0	0
			0	0	0
19	Efforts to treat underlying OSA should be maximized; in sleepy patients who are sub-optimally treated for OSA, clinicians should consider pharmacological alerting therapy for EDS if there is a good risk:benefit ratio.	Strongly agree Agree with reservation Undecided Disagree Strongly disagree	60	90	100
			30	10	0
			10	0	0
			0	0	0
			0	0	0
20	If OSA is being optimally treated and the patient continues to have residual EDS, the clinician should recognize and ensure treatment of any other causes of EDS, including sleep deprivation, psychiatric illness (in particular depression), idiopathic hypersomnia, narcolepsy, circadian rhythm disorders, hypoventilation, neurological diseases, chronic medical conditions (eg, hypothyroidism), concomitant medications, or illicit drug use.	Strongly agree Agree with reservation Undecided Disagree Strongly disagree	90	90	100
			10	10	0
			0	0	0
			0	0	0
			0	0	0
21	If the clinician suspects the cause of EDS is due to an etiology other than OSA, additional diagnostic testing may be required, including a Multiple Sleep Latency Test following a polysomnography on airway therapy (to rule out other disorders of hypersomnolence, such as narcolepsy), actigraphy, or sleep diaries (to rule out insufficient sleep).	Strongly agree Agree with reservation Undecided Disagree Strongly disagree	100	90	100
			0	10	0
			0	0	0
			0	0	0
			0	0	0
22	A patient can have a diagnosis of OSA with comorbid IH if the patient was previously diagnosed with IH (with OSA excluded) and later developed OSA. If a patient with OSA was not previously diagnosed with IH, the persistent EDS should not be considered IH.	Strongly agree Agree with reservation Undecided Disagree Strongly disagree	50	90	90
			40	10	10
			10	0	0
			0	0	0
			0	0	0
23	To distinguish between a diagnosis of EDS due to OSA and OSA with hypersomnia due to other disorders, it is important to take a comprehensive medical history to establish a timeline as to when symptoms occurred.	Strongly agree Agree with reservation Undecided Disagree Strongly disagree	90	100	100
			10	0	0
			0	0	0
			0	0	0
			0	0	0

a Consensus was considered to be achieved if ≥ 80% of respondents agreed or agreed with reservation.

b Final statements shown. CPAP = continuous positive airway pressure, EDS = excessive daytime sleepiness, ESS = Epworth Sleepiness Scale, IH = idiopathic hypersomnia, OA = oral appliance, OSA = obstructive sleep apnoea, PAP = positive airway pressure, PROMIS SRI=Patient-Reported Outcomes Measurement Information System Sleep-Related Impairment, QoL = quality of life.

**Table 5:** Level of agreement/disagreement with each statement during each round:^a^ When and how pharmacological treatment for EDS in OSA should be initiated.

Statement Number	Statement^b^	Response	Response Rate (%)		
			Round 1	Round 2	Round 3
24	Pharmacological treatment of residual EDS due to OSA should be considered if efficacy of therapy for OSA has been optimised; possible underlying medical, neurologic, psychiatric, and sleep disorders have been addressed to achieve adequate sleep; and there is good risk:benefit ratio with therapy.	Strongly agree Agree with reservation Undecided Disagree Strongly disagree	70	100	100
			30	0	0
			0	0	0
			0	0	0
			0	0	0
25	Pharmacological treatment of residual EDS due to OSA should be considered if EDS substantially reduces quality of life, impairs ability to work or learn, or increases risk of motor vehicle or workplace accidents. It is recognised these agents may improve EDS but do not treat sleep disordered breathing or reduce medical consequences of OSA.	Strongly agree Agree with reservation Undecided Disagree Strongly disagree	100	100	100
			0	0	0
			0	0	0
			0	0	0
			0	0	0
26	In selected cases with EDS, pharmacological treatment may be considered for patients who are not adherent to or could not be treated with primary therapies. However, ongoing clinical efforts to treat OSA adequately are strongly recommended.	Strongly agree Agree with reservation Undecided Disagree Strongly disagree	70	80	100
			10	20	0
			20	0	0
			0	0	0
			0	0	0
27	The selection of wake-promoting drugs should be based on potential efficacy, safety, and risk:benefit ratio of therapy and cardiovascular, central nervous system, and psychiatric comorbidities, as well as accompanying sleep disorders.	Strongly agree Agree with reservation Undecided Disagree Strongly disagree	80	100	100
			20	0	0
			0	0	0
			0	0	0
			0	0	0
28	The use of pharmacological treatment of EDS must not impede the early diagnosis and management of OSA and underlying conditions. Pharmacological treatment of EDS due to OSA may be considered in patients after motor vehicle or workplace accidents or near-accidents	Strongly agree Agree with reservation Undecided Disagree Strongly disagree	90	100	100
			10	0	0
			0	0	0
			0	0	0
			0	0	0
29	Validated patient reported outcome measures (Table 2) facilitate clinical assessment of the presence and severity of EDS, including the potential impact of EDS on QOL as well as safety. These measures supplement clinical assessment of severity and change with OSA management.	Strongly agree Agree with reservation Undecided Disagree Strongly disagree	80	80	100
			20	20	0
			0	0	0
			0	0	0
			0	0	0
30	In selected cases, objective measurements of EDS due to OSA may be performed to help resolve discrepancies in clinical history and questionnaire responses, rule out other sleep disorders, or evaluate efficacy of therapy. Measurements may include Multiple Sleep Latency Test, Maintenance of Wakefulness Test, or vigilance testing.	Strongly agree Agree with reservation Undecided Disagree Strongly disagree	60	70	100
			20	30	0
			10	0	0
			10	0	0
			0	0	0
31	The safety and efficacy of pharmacological treatment of residual EDS due to OSA should be clinically assessed and monitored periodically (frequency depending on clinical context) as part of a comprehensive continuum of care.	Strongly agree Agree with reservation Undecided Disagree Strongly disagree	60	100	100
			30	0	0
			10	0	0
			0	0	0
			0	0	0
32	For the quantification of severity and impact of EDS due to OSA Agree with reservation and the effects of therapeutic interventions, validated subjective and objective testing can complement the clinical assessment (Table 2).	Strongly agree Undecided Disagree Strongly disagree	90	90	90
			10	0	10
			0	10	0
			0	0	0
			0	0	0

a Consensus was considered to be achieved if ≥ 80% of respondents agreed or agreed with reservation.

b Final statements shown. CPAP = continuous positive airway pressure, EDS = excessive daytime sleepiness, ESS = Epworth Sleepiness Scale, IH = idiopathic hypersomnia, OA = oral appliance, OSA = obstructive sleep apnoea, PAP = positive airway pressure, PROMIS SRI=Patient-Reported Outcomes Measurement Information System Sleep-Related Impairment, QoL = quality of life.

## Abbreviations:

AHI: apnoea-hypopnoea index
CPAP: continuous positive airway pressure
EDS: excessive daytime sleepiness
ESS: Epworth Sleepiness Scale
IH: idiopathic hypersomnia
MWT: Maintenance of Wakefulness Test
MSLT: Multiple Sleep Latency Test
OSA: obstructive sleep apnoea
PROMIS SRI: Patient-Reported Outcomes Measurement Information System Sleep Related Impairment scale
