# Supplementary material for: Recommendations for clinical management of excessive daytime sleepiness in obstructive sleep apnoea – A Delphi consensus study
Source: Sleep Med. Author manuscript; Available in PMC 2024 Jun 1. (PMC10841517; doi:10.1016/j.sleep.2023.10.001)
Supplement: sup [file NIHMS1938130-supplement-sup.docx]

**Recommendations for Clinical Management of Excessive Daytime Sleepiness in Obstructive Sleep Apnoea – A Delphi Consensus Study**

Joerg S. Steier, MD^a†^, Richard K. Bogan, MD^b^, Irene M. Cano-Pumarega, MD^c^,
John A. Fleetham, MD^d^, Giuseppe Insalaco, MD^e^, Chitra Lal, MD^f^,
Jean-Louis Pépin, MD^g^, Winfried J. Randerath, MD^h^,
Susan Redline, MD, MPH^i^, Atul Malhotra, MD^j†^

^a^Centre for Human and Applied Physiological Sciences, Faculty of Life Sciences and Medicine, King’s College London, London, UK; ^b^Medical University of South Carolina, Charleston, SC, USA; ^c^Sleep Unit, Respiratory Department, Ramón y Cajal University Hospital, IRYCIS, CIBERES, Madrid, Spain; ^d^Department of Medicine, University of British Columbia, Vancouver, BC, Canada; ^e^Institute of Translational Pharmacology, Italian National Research Council, Palermo, Italy; ^f^Pulmonary, Critical Care, Allergy and Sleep Medicine, Medical University of South Carolina, College of Medicine, Charleston, SC, USA; ^g^Grenoble Alpes University, INSERM, University Hospital Grenoble Alpes, HP2, Grenoble, France; ^h^Institute of Pneumology at the University of Cologne, Bethanien Hospital, Clinic for Pneumology and Allergology, Centre of Sleep Medicine and Respiratory Care, Solingen, Germany; ^i^Brigham and Women’s Hospital, Harvard Medical School, Boston, MA, USA; ^j^University of California, San Diego Health, La Jolla, CA, USA

^†^Study Co-Chairs

**Supplementary Fig. 1.** Modified Delphi process

**
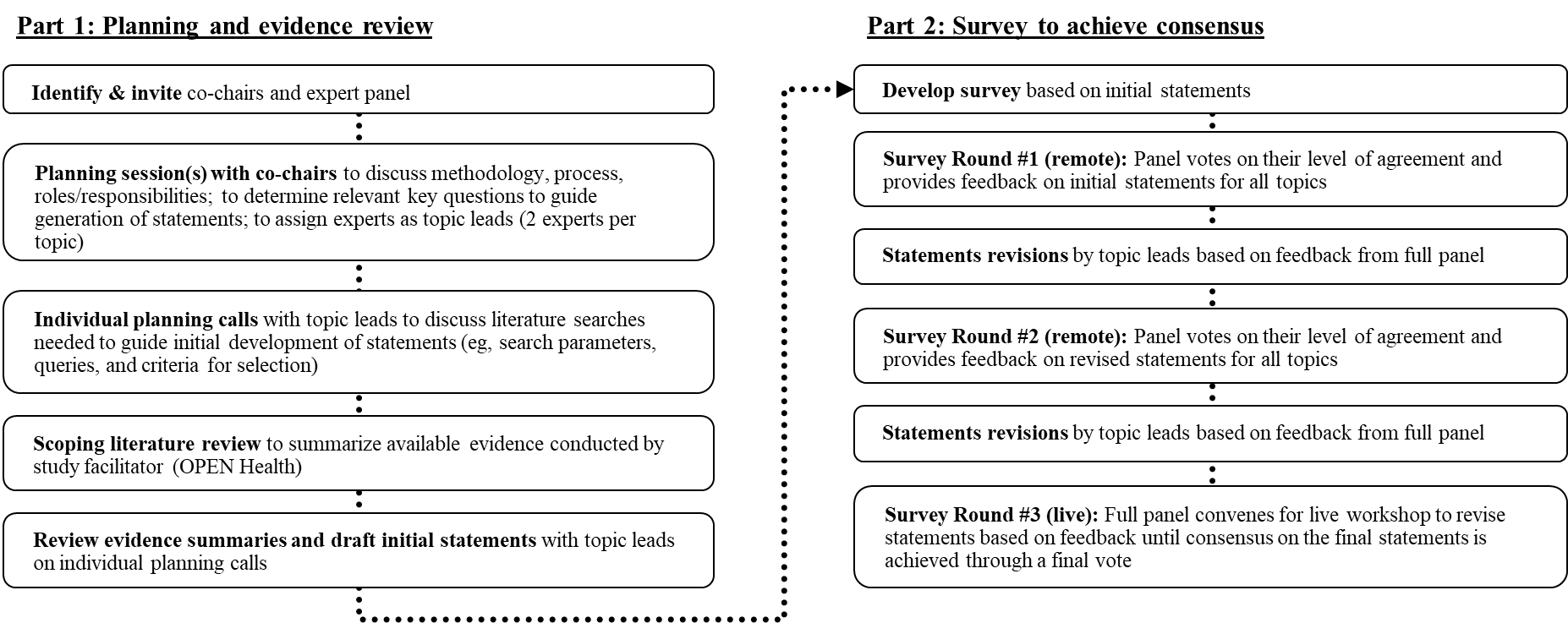
**

**Supplementary Fig. 2.** Statement agreement* based on consensus voting across three rounds of review

*Agreement based on percentage of respondents who *strongly agree* or *agree with reservation*. ^†^During Round #3, two statements were combined, resulting in a total of 32 statements in Round #3, compared with 33 statements in Rounds #1 and #2.


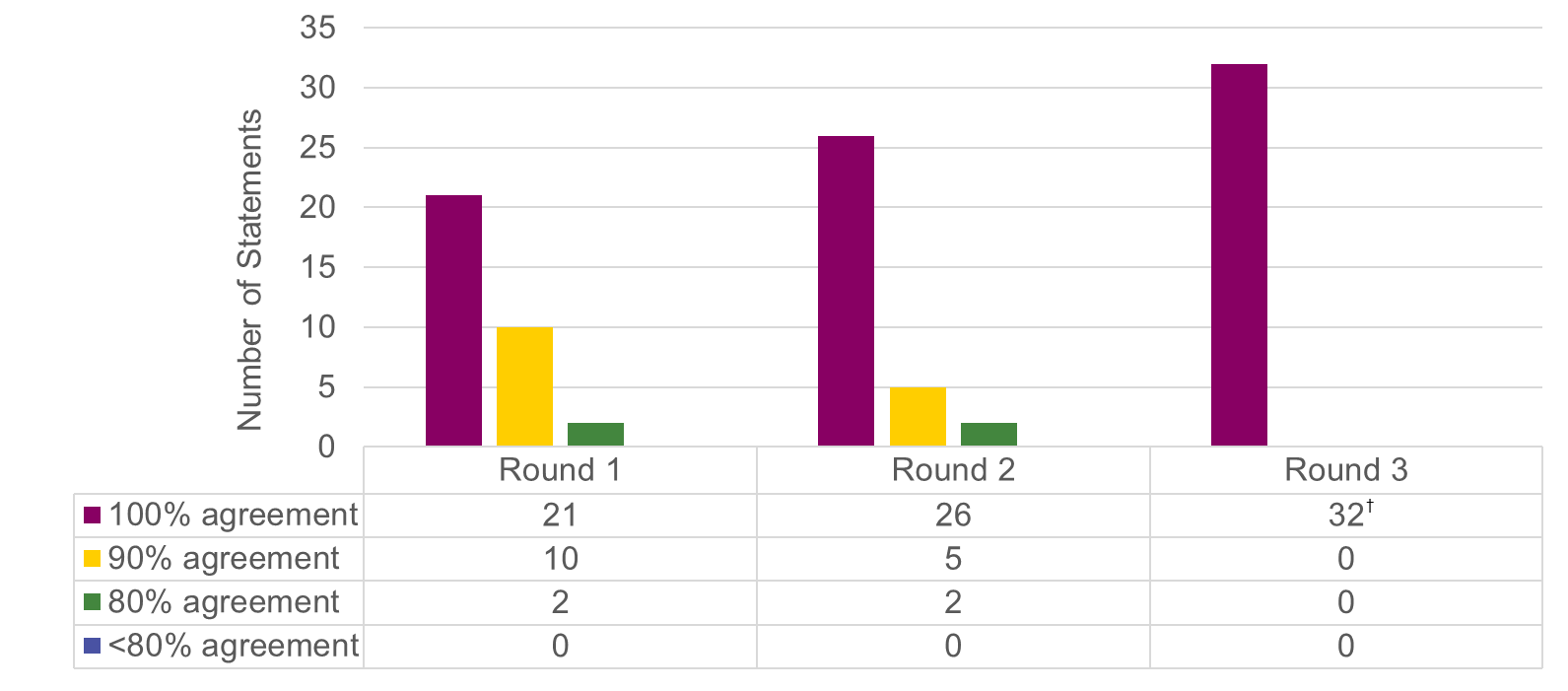


**Supplementary Table 1. Questions used to guide the development of the statements.**

| **Question** | **Associated Statement(s)** |
| --- | --- |
| **Topic 1: How to define and evaluate EDS** | |
| How frequently should sleepiness be evaluated following a diagnosis of OSA to ensure adequate resolution of EDS and ongoing maintenance of wakefulness? | #1 |
| How are sleepiness and EDS defined in a real-world clinical population (definitions of objective and subjective sleepiness)? | #2 |
| What tools should be used to assess EDS in OSA within a clinical setting? | #3–5 |
| What are the outcomes on those tools indicate that further management of EDS is required? | #6 |
| How should differences between objective and subjective measures of sleepiness be addressed in clinical practice? | #7 |
| **Topic 2: How to define and evaluate residual EDS due to OSA in patients treated with primary OSA therapy** | |
| Within a real-world clinical population, how do you define optimal treatment of sleep apnea? What are the parameters that equate to optimized CPAP therapy as it pertains to utilization and duration of CPAP therapy? | #8–10 |
| Within a real-world clinical population, how do you define adequate amounts of sleep and establish whether this criterion has been met for an individual patient? | #11–12 |
| Within a real-world clinical population, what are the potential causes and/or mechanisms of residual EDS in a patient with OSA and how do you sufficiently rule them out? | #13–14 |
| **Topic 3: How to address specific clinical challenges** | |
| How do you approach patients with EDS who are intolerant to CPAP therapy (or other primary therapies) or have attempted/are attempting therapy? | #15–19 |
| How do you manage a patient who has persistent EDS that is likely due to other reasons beyond OSA? How does it differ from EDS due to OSA specifically? | #20–21 |
| How do you distinguish between a diagnosis of EDS in OSA and OSA with comorbid idiopathic hypersomnia? Can a patient have a diagnosis of OSA with comorbid idiopathic hypersomnia? | #22–23 |
| **Topic 4: When and how pharmacological treatment for EDS due to OSA should be initiated** | |
| Which clinical considerations and/or patient-specific factors should guide decision making related to initiation of pharmacological treatment? | #24–27 |
| Are there concerns that would lead you to consider implementing pharmacotherapy earlier in some patients (eg, driving risk, patient worries about losing their job)? | #28 |
| What metrics should be used to determine success of a pharmacological treatment? | #29–32 |

CPAP, continuous positive airway pressure; EDS, excessive daytime sleepiness; OSA, obstructive sleep apnoea.

**Supplementary Table 2.** Literature search queries

| **Set#** | **Searched for** | **Results** | **Search #** |
| --- | --- | --- | --- |
| S1 | TI,AB(“sleep apnea” OR “sleep apnoea” OR "OSA") OR EMB.EXACT.EXPLODE(“sleep disordered breathing”) OR MESH.EXACT.EXPLODE(“Sleep Apnea, Obstructive”) | 129964* |  |
| S2 | (TI,AB(sleepy OR sleepiness OR drowsy OR drowsiness OR somnolence) AND TI,AB(excessive OR persistent OR residual OR refractory OR "day time" OR daytime)) OR TI,AB(hypersomnia) OR EMB.EXACT("excessive daytime sleepiness") OR MESH.EXACT.EXPLODE("Disorders of Excessive Somnolence") | 40566* |  |
| S3 | TI,AB(frequency OR frequently OR intervals) AND TI,AB(evaluation OR evaluated OR assessment OR assessed OR monitor OR monitoring OR monitored) | 1586614* |  |
| **S4** | **S1 AND S2 AND S3 AND LA(English)** | **758** | **1A** |
| S5 | TI(tool OR tools OR test OR tests OR scale OR scales OR score OR scores OR measure OR measures OR measurement OR measurements OR assessment OR assessments OR instrument OR instruments) | 2339560* |  |
| S6 | TI,AB(recommend OR recommended OR robust OR validity OR valid OR usability OR real-world OR real OR practice OR psychometric OR practical) | 5688640* |  |
| **S7** | **S2 AND S5 AND S6 AND LA(English)** | **440** | **1B** |
| S8 | TI,AB((sleep OR sleeping) NEAR/3 (duration OR hours OR time OR length)) | 76205* |  |
| S9 | TI,AB(adult OR adults OR elderly) | 3588890* |  |
| S10 | TI(impact OR ideal OR quality OR recommended OR recommend OR adequate OR sufficient OR short OR optimal OR optimum) | 1612878* |  |
| S11 | TI,AB(general OR healthy OR population-based) | 4690140* |  |
| **S12** | **S8 AND S9 AND S10 AND S11 AND LA(English)** | **412** | **2A** |
| S13 | TI("positive airway pressure" OR PAP OR CPAP) OR EMB.EXACT.EXPLODE("continuous positive airway pressure") OR MESH.EXACT("Continuous Positive Airway Pressure") | 30626* |  |
| S14 | TI(impact OR ideal OR quality OR recommended OR recommend OR adequate OR sufficient OR short OR optimal OR optimum) | 1612878* |  |
| **S15** | **S1 AND S2 AND S13 AND S14 AND LA(English)** | **120** | **2B** |
| S16 | TI,AB(refuse OR refusal OR refused OR intolerant OR intolerance OR refractory OR unable OR "not responsive" OR unresponsive) | 847006* |  |
| **S17** | **S1 AND S2 AND S13 AND S16 AND LA(English)** | **92** | **3A** |
| S18 | TI,AB(comorbid OR comorbidity OR comorbidities OR coincident OR coincidence OR overlapping OR underlying OR "differential diagnosis") | 2337668* |  |
| **S19** | **S1 AND S2 AND S18 AND LA(English)** | **1157** | **2C** |
| S20 | TI(“sleep apnea” OR “sleep apnoea” OR "OSA") OR EMB.EXACT.EXPLODE(“sleep disordered breathing”) OR MESH.EXACT.EXPLODE(“Sleep Apnea, Obstructive”) | 106056* |  |
| S21 | (TI(sleepy OR sleepiness OR drowsy OR drowsiness OR fatigue OR fatigued OR somnolence) AND TI,AB(excessive OR persistent OR residual OR refractory OR "day time" OR daytime)) OR TI(hypersomnia) OR EMB.EXACT("excessive daytime sleepiness") OR MESH.EXACT.EXPLODE("Disorders of Excessive Somnolence") | 17087* |  |
| S22 | TI(management OR treatment OR therapy OR guidelines OR recommendations) | 5332017* |  |
| **S23** | **S20 AND S21 AND S22 AND LA(English)** | **256** | **3B** |
| S24 | TI("idiopathic hypersomnia") OR EMB.EXACT(hypersomnia) OR MESH.EXACT("Idiopathic Hypersomnia") | 5139* |  |
| S25 | TI(guidelines OR recommendations OR diagnosis OR diagnostic) | 1557618* |  |
| **S26** | **S25 AND (S4 OR (S21 AND S20)) AND LA(English)** | **55** | **3C** |
| S27 | TI(management OR treatment OR therapy OR guidelines OR recommendations OR post-marketing OR safety OR “side effect”) OR TI,AB(driving OR “motor vehicle” OR cognition OR workplace OR work OR employment OR “quality of life” OR memory OR executive OR function OR functional) | 16491771* |  |
| S28 | TI,AB(modafinil OR Provigil OR armodafinil OR Nuvigil OR "JZP-110" OR solriamfetol OR methylphenidate OR Ritalin OR amphetamine OR Adderall OR Evekeo OR Metadate OR methylin OR dextroamphetamine OR Dexedrine OR Spansule OR Procentra OR Zenzedi OR “wake-promoting” OR “wake promoting”) OR EMB.EXACT(modafinil OR armodafinil OR methylphenidate OR dexamphetamine) OR MESH.EXACT(Modafinil OR Methylphenidate OR Amphetamine OR Dextroamphetamine) | 105453* |  |
| **S29** | **S1 AND S2 AND S27 AND S28 AND LA(English)** | **382** | **4** |

*Count includes duplicates between Embase and MEDLINE databases. Note that combined searches were deduplicated.

**Supplementary Table 3.** Summary of literature searches

1. **Topic 1: Sleepiness definition and evaluation – how to define and evaluate EDS**

|  | **Search 1A** | **Search 1B** |
| --- | --- | --- |
| Overall topic | Evaluation/assessment of EDS | Tools to assess EDS |
| Population | OSA with EDS | Any persons with EDS |
| Intervention | Any | Any |
| Outcome | Patient monitoring frequency | Tools to measure sleepiness – psychometric properties, tool-based management decisions |
| Study design | Longitudinal RWE, guidelines | RWE, psychometric literature, guidelines |
| Questions | How frequently should sleepiness be evaluated following diagnosis of OSA to ensure adequate resolution and ongoing maintenance of wakefulness? | What tools should be used to assess EDS in OSA within a clinical setting?  What outcomes on EDS assessment tools indicate further EDS management is required (eg, is it ≥11 on the ESS or could 8 on ESS suggest a need to follow up)? |
| Related subtopics | ─ | Cover objective and subjective sleepiness |
| Search query | S4 | S7 |
| Hits (pre-screening) | 758 | 427 |
| Selections | 8 | 51 |
| Potential interest | 3 | 10 |

1. **Topic 2: Residual/persistent sleepiness definition – how to define and evaluate residual EDS due to OSA in patients treated with primary OSA therapy**

|  | **Search 2A** | **Search 2B** | **Search 2C** |
| --- | --- | --- | --- |
| Overall topic | Adequate amounts of sleep | Optimal treatment for OSA | Potential causes/mechanisms of residual EDS |
| Population | General | EDS in OSA | Any patients with EDS |
| Intervention | None | CPAP and non-CPAP | Any |
| Outcome | Relationship between sleep and morbidity/mortality | Relationship between CPAP and EDS | Identification of EDS cause |
| Study design | Longitudinal RWE | Trials, RWE | RWE |
| Questions | Within a real-world clinical population, how do you define adequate amounts of sleep and establish whether this criterion has been met for an individual patient? | Within a real-world clinical population, how do you define optimal treatment of sleep apnea?  What are the parameters that equate to optimized CPAP therapy as it pertains to utilization and duration on CPAP therapy? | Within a real-world clinical population, what are the potential causes and/or mechanisms of residual EDS in a patient with OSA and how do you sufficiently rule them out? |
| Related subtopics | ─ | ─ | ─ |
| Search query | S12 | S15 | S19 |
| Hits (pre-screening) | 412 | 120 | 1157 |
| Selections | 19 | 23 | 40 |
| Potential interest | 1 | 6 | 5 |

1. **Topic 3: Practical recommendations – how to address specific clinical challenges**

|  | **Search 3A** | **Search 3B** | **Search 3C** |
| --- | --- | --- | --- |
| Overall topic | Patients with EDS in OSA who refuse CPAP | Management of EDS in OSA due to other conditions | Idiopathic hypersomnia diagnosis vs OSA diagnosis |
| Population | EDS in OSA | EDS in OSA | IH or OSA with EDS |
| Intervention | Not CPAP | Any | Any |
| Outcome | Population size, treatment pathways | Patient management | Diagnostic criteria |
| Study design | Trials, RWE | RWE, guidelines | Guidelines |
| Questions | How do you approach patients with EDS who are intolerant to PAP therapy or have attempted/are attempting therapy?  How many people refuse CPAP? | How do you manage a patient who has persistent EDS that is likely due to other reasons beyond OSA? What should be done to treat the other conditions?  How does EDS due to other conditions differ from EDS due to OSA specifically? | How do you distinguish between a diagnosis of EDS in OSA and OSA with comorbid idiopathic hypersomnia?  Can a patient have a diagnosis of OSA with comorbid idiopathic hypersomnia? |
| Related subtopics | ─ | ─ | ─ |
| Search query | S17 | S23 | S26 |
| Hits (pre-screening) | 92 | 256 | 55 |
| Selections | 25 | 13 | 2 |
| Potential interest | 0 | 2 | 2 |

1. **Topic 4: Management of residual EDS – when and how pharmacological treatment for EDS due to OSA should be initiated**

|  | **Search 4A** |
| --- | --- |
| Overall topic | When/how/why to initiate pharmacological therapy |
| Population | OSA with EDS |
| Intervention | Pharmacological |
| Outcome | Patient management |
| Study design | Trials, RWE, guidelines |
| Questions | When and how should pharmacological treatment for EDS in OSA be initiated?  Which clinical considerations and/or patient-specific factors should guide decision making related to initiation of pharmacological treatment?  Are there concerns that would lead you to consider implementing pharmacotherapy earlier in some patients (eg, driving risk, patient worries about losing their job)?  What metrics should be used to determine success of a pharmacological treatment (must align with Group 1 Search 1B)? |
| Related subtopics | ─ |
| Search query | S29 |
| Hits (pre-screening) | 382 |
| Selections | 40 |
| Potential interest | 8 |

For more information on search queries, see **Supplementary** **Table 2**.

CPAP, continuous positive airway pressure; EDS, excessive daytime sleepiness; ESS, Epworth Sleepiness Scale; IH, idiopathic hypersomnia; OSA, obstructive sleep apnoea; PAP, positive airway pressure; RWE, real-world evidence.

**Supplementary Table 4. Supporting evidence for Search 1A: How frequently should sleepiness be evaluated following diagnosis of OSA to ensure adequate resolution and ongoing maintenance of wakefulness?**

| **Supporting evidence for Search 1A: How frequently should sleepiness be evaluated following diagnosis of OSA to ensure adequate resolution and ongoing maintenance of wakefulness?** | | | | |
| --- | --- | --- | --- | --- |
| **Study** | **Article type/**  **Study design** | **Study population** | **Setting** | **Recommended length of follow-up** |
| Patil SP, et al. Treatment of adult obstructive sleep apnea with positive airway pressure: an American Academy of Sleep Medicine clinical practice guideline. *J Clin Sleep Med.* 2019;15(2):335-343. | Practice guideline | Patients with OSA treated with PAP | N/A | - Initial treatment of OSA requires close monitoring and early identification of difficulties with PAP use, as adherence over the first few days to weeks has been shown to predict long-term adherence. - The timing of adequate follow-up after treatment is initiated will vary depending on patient circumstances. - Patients should be followed in the initial weeks to months after PAP initiation to promote adherence and assess response to treatment. - Subsequently, yearly evaluation by a trained HCP is reasonable, although longer periods of follow-up may be appropriate for selected patients who are highly adherent to PAP therapy, have sustained resolution of OSA-related symptoms, and have no concerns regarding their PAP therapy. - In contrast, patients with persistent or recurrent sleep-related complaints or persistent difficulties with PAP use should receive more frequent follow-up to address their issues. |
| Tietjens JR, et al. Obstructive sleep apnea in cardiovascular disease: a review of the literature and proposed multidisciplinary clinical management strategy. *J Am Heart Assoc*. 2019;8(1):e010440. | Literature review/practice guidelines | Patients with OSA and CVD | N/A | - Due to the high prevalence of OSA in patients with CVD, this article recommends screening for SDB symptoms (eg, STOP-Bang, ESS, or Berlin questionnaires) in patients with established CVD during routine preoperative assessment. - Diagnostic testing is suggested for all patients, including those with no SDB symptoms, with these forms of CVD:   - Resistant hypertension, defined as inadequately controlled blood pressure despite therapy with ≥3 oral antihypertensive agents including a diuretic, or adequate blood pressure control requiring ≥4 agents.   - Pulmonary hypertension.   - Recurrent atrial fibrillation following either cardioversion or ablation. |
| Aurora RN, et al. Quality measures for the care of adult patients with obstructive sleep apnea. *J Clin Sleep Med.* 2015;11(3):357-383. | Literature review | Patients ≥18 y with OSA | N/A | - Patients ≥18 y with suspected OSA should have a record of their presenting symptoms (including snoring and sleepiness) at initial evaluation, and should be assessed regularly for symptom development and progression. - Untreated OSA is associated with sleepiness and lower QoL; therefore, adherence to primary therapy (PAP) should be assessed. - Self-reported sleepiness should also be monitored in patients being treated for OSA. During history, HCPs should follow sleepiness or utilize a validated subjective sleepiness scale (eg, ESS). |
| Ronksley PE, et al. Excessive daytime sleepiness is associated with increased health care utilization among patients referred for assessment of OSA. *Sleep.* 2011;34(3): 363-370. | Cross-sectional study | Patients ≥18 y referred for sleep diagnostic testing by GP due to suspected sleep disorder (including OSA) (n=2149) | Clinic-based | N/A |
| Weaver TE. Outcome measurement in sleep medicine practice and research. Part 1: assessment of  symptoms, subjective and objective daytime  sleepiness, health-related quality of life and functional status. *Sleep Med Rev.* 2001;5(2):103-128. | Literature review | N/A | N/A | - Initial follow-up assessment of subjective sleepiness, HRQoL, functional status, mood, and adherence should occur during the first week of treatment to determine effectiveness. |
| He K, et al. Sleep-disordered breathing and excessive daytime sleepiness. *Sleep Med Clin.* 2017;12(3):369-382. | Review | Patients with EDS in OSA | N/A | - Sleepiness is commonly caused by insufficient sleep; therefore, providers should ask patients about their sleep-wake schedules, as differences of >1 h between weekdays and weekends can signify work-related sleep restriction. - Some medical conditions can mimic or contribute to sleepiness (depression, hypothyroidism, iron deficiency, vitamin D deficiency); providers should determine use of prescription/nonprescription sedatives or alerting substances. - Lab testing (MSLT, MWT, metabolic panel, blood counts, thyroid function, testosterone levels in men, iron studies, urine drug screen, vitamin B12 and D levels) for EDS in OSA is typically only necessary to differentially diagnose the underlying cause of EDS (ie, if symptoms indicate a comorbid or different cause than OSA) or in patients who have residual sleepiness despite optimal treatment of OSA. - Lifelong monitoring and treatment are needed due to the chronic nature of OSA. After therapy initiation, patients should receive long-term follow-up, including ESS assessment of EDS. - For patients on PAP, equipment maintenance and data monitoring should take place periodically to ensure efficiency and adherence. Non-PAP therapy efficacy should be determined with a sleep study in patients with moderate to severe OSA or mild OSA with residual EDS. - In some individuals who achieve significant, sustained weight loss and EDS resolution, treatment can be discontinued after evaluation. |
| Epstein LJ, et al. Clinical guideline for the evaluation, management, and long-term care of obstructive sleep apnea in adults. *J Clin Sleep Med.* 2009;5(3):263-276. | Practice guidelines | Adult patients with OSA | N/A | - Routine health maintenance evaluations should include patient history of snoring and EDS and an assessment for the presence of obesity, retrognathia, or hypertension; positive findings should lead to a more comprehensive exam. - Comprehensive sleep history in patients suspected of having OSA should include assessments for snoring, apneas, gasping/choking episodes, unexplainable EDS (including ESS assessment), total sleep amount, nocturia, morning headaches, sleep fragmentation/maintenance, insomnia, and decreased concentration/memory. - Patients with OSA should undergo long-term management, especially those on chronic therapy, to monitor adherence, side effects, complications, and continued resolution of symptoms. - Those with OSA elimination due to weight loss or surgery should be monitored for risk factors and symptom return. |
| Yaremchuk K, et al. Change in Epworth Sleepiness Scale after surgical treatment of obstructive sleep apnea. *Laryngoscope*. 2011;121(7):1590-1593. | Retrospective case series with chart review | Adult patients undergoing uvulopalatopharyngo-plasty, tonsillectomy, or radiofrequency ablation of the base of the tongue for OSA | Data were obtained from the hospital’s medical record system | - Postoperatively, patients completed the ESS questionnaire at a median of 6 wk and repeated PSG at a median of 3 mo. |

CPAP, continuous positive airway pressure; CVD, cardiovascular disease; EDS, excessive daytime sleepiness; ESS, Epworth Sleepiness Scale; GP, general practitioner; HCP, healthcare professional; HRQL, health-related quality of life; min, minutes; MSLT, multiple sleep latency test; MWT, maintenance of wakefulness test; OSA, obstructive sleep apnea; PAP, positive airway pressure; PSG, polysomnography; QoL, quality of life; RCT, randomized controlled trial; SDB, sleep-disordered breathing; STOP-Bang, Snoring, Tiredness, Observed apnea, blood Pressure, Body mass index, Age, Neck circumference and Gender; T2DM, type 2 diabetes mellitus.

**Supplementary Table 5. Supporting evidence for Search 1B: What tools should be used to assess EDS in OSA within a clinical setting, and what are the outcomes on those tools that require further management of their sleepiness?**

| **Supporting evidence for Search 1B: What tools should be used to assess EDS in OSA within a clinical setting, and what are the outcomes on those tools that require further management of their sleepiness?** | | | | | |
| --- | --- | --- | --- | --- | --- |
| **Study** | **Article type/**  **Study design** | **Study population** | **Setting** | **Tool(s) used in study** | **Key finding(s)** |
| Nishiyama T, et al. Criterion validity of the Pittsburgh Sleep Quality Index and Epworth Sleepiness Scale for the diagnosis of sleep disorders. *Sleep Med.* 2014;15:422-429. | Psychometric validation study and meta-analysis | Psychometric analysis: Untreated patients with a variety of sleep disorders (OSA, PLMD, RBD, and narcolepsy) (N=432)  Meta-analysis: Patients with OSA (N=1280 across 7 studies) | Clinic | ESS and PSQI | - Neither the ESS nor the PSQI were accurate for identifying sleep disorders (AUC<0.9; pooled area under ROC curve=0.48). - The ESS and PSQI are more influenced by psychological symptoms than PSG indices; therefore, these tools should not be used to screen for or diagnose PSG-defined sleep disorders. |
| Kaplan KA, et al. Psychometric scales measuring hypersomnolence. *Curr Sleep Med Rep.* 2020;6(3):111-120. | Review | Patients with mood disorders and idiopathic hypersomnia | N/A | - SSS - KSS - ESS - pESS - BSI - ODSI - SWIFT - ToDSS - SWAI - DSS - Resistance to Sleepiness Scale - FACES – Sleepiness Subscale - Iowa Sleep Disturbances Inventory – Excessive Sleep Subscale - PDSS - Cleveland Adolescent Sleepiness Questionnaire - ESS for Children and Adolescents - Children’s Sleep Habits Questionnaire – Daytime Sleepiness Subscale - School Sleep Habits Survey – Sleepiness Subscale - Pictorial Sleepiness Scale | - Collectively, these measures have at least 3 limitations to be addressed by future research:   - Data on test-retest reliability and sensitivity to clinical intervention is not available for some instruments, limiting their use.   - Complaints of hypersomnolence are usually evaluated with objective measures (PSG, MSLT), yet many of the instruments do not report convergent validity with objective sleep assessment; other scales show indistinct relationships to objective measures (eg, ESS scores have been correlated with MSLT scores in only some research).   - Few of the scales are designed to measure long nighttime sleep duration and only consider long sleep during the day (eg, the Iowa Sleep Disturbances Inventory – Excessive Sleep Subscale). |
| Murray BJ. Subjective and objective assessment of hypersomnolence. *Sleep Med Clin*. 2020;15(2):167-176. | Review | N/A | N/A | - Subjective assessments:   - Sleep log   - ESS   - BSI   - ODSI   - SSS   - KSS   - SWIFT   - ToDSS   - PSQI   - Leeds Sleep Evaluation Questionnaire   - Pediatric Scales - Objective assessments:   - Clinical observation   - Clinical neurophysiology   - MSLT   - MWT   - Ambulatory EEG   - Actigraphy   - PVT   - OSLER   - SART   - Eyelid movement   - Eye movements   - Pupillography   - Driving simulators | - Problems with subjective assessments:   - Patients who have subjective sleepiness may not realize the severity of their impairment.   - A disconnect may exist between subjective (ESS) and objective (MSLT) measures of sleepiness.   - Patients may exaggerate symptoms to seek stimulants.   - Interpretation of subjective scales is best done when the clinician can gauge the reliability of the patient and their insight.   - Tracking changes on these scales may be more useful than using absolute values to establish a diagnosis. - Relying on objective measures may be needed when there are concerns about patient reliability and/or significant implications of abnormal test results, although refinements and standardization of objective scales are required.   - The MSLT and MWT continue to be the best standardized tests to date despite their problems.   - A combination of physiologic assessments may provide more information. - Generally, objective and subjective scales should be used together to assess sleepiness. |
| Silva FSL, et al. Are the International Classification of Functioning, Disability, and Health (ICF) domains considered in the obstructive sleep apnea instruments? An integrative review. *Sleep Breath.* 2021;25(2):1089-1100. | Integrative literature review (includes articles published in English, Portuguese, and Spanish) | Adults with OSA | N/A | PSQI, ESS, BQ, FOSQ, SAQLI, GSAQ, SEMSA, SOS, QSQ, SBQ, FOSQ-10, SQS, CCUQ, MOSAS, pSSAS, CCSESA-J in relation to the ICF | - The validated OSA instruments included in this review have significant ICF concepts with the prevalence of the function domain, and the remaining domains being poorly explored. - Further studies are needed to address the functioning of patients with OSA. - Authors proposed an instrument that incorporates concepts of the biopsychosocial mode to allow understanding of the influence of personal/ environmental factors, activity, and participation components in functioning. |
| Hurlston A, et al. The Epworth Sleepiness Scale in service members with sleep disorders. *Mil Med.* 2019;184(11-12):e701-e707. | Retrospective review | Military personnel who were referred for evaluation to a sleep disorders center (n=488) | Academic military sleep disorders center | ESS | - Results suggested that there is a 59% likelihood that random participants meeting diagnostic criteria for only insomnia would have higher ESS scores than those with only OSA. - Elevated ESS score indicates OSA, insomnia, or COMISA and should be evaluated accordingly. |
| Kaplan KA, et al. Development and validation of the Hypersomnia Severity Index (HSI): a measure to assess hypersomnia severity and impairment in psychiatric disorders. *Psychiatry Res*. 2019;281:112547. | Online research study | Undergraduates from University of California, Berkley (n=381) and individuals with MDD (n=21) and BD (n=89) | Online | HSI | - Internal consistency of the Distress/Impairment factor, as well as HSI overall, was high, although for the Hypersomnia Symptoms factor, Cronbach’s alpha coefficients decreased. - Optimal cutoff score = 10. - High test-retest reliability and stability in the subset of patients with BD. - Future studies are needed to determine whether the HSI is sensitive to treatment response. |
| Virtanen I, et al. Can real-life driving ability be predicted by the Maintenance of Wakefulness Test? *Traffic Inj Prev*. 2019;20:601-606. | Retrospective chart analysis | Patients with an MWT and a driving ability assessment (n=63) | Clinic | MWT | - Patients with MWT mean sleep latency ≥32.6 min (without posterior alpha activity or theta of drowsiness) can reliably be permitted to drive. - Importantly, MWT latency did not reliably predict successful performance on a 2-h driving test. |
| Foster S, et al. Excessive daytime somnolence in patients with obstructive sleep apnea adequately treated with positive airway pressure. *Sleep*. 2018; 41(suppl 1):A193. [Abstract] | Retrospective review | Adult patients with OSA and EDS (ESS >10) on PAP therapy (4 h/night for ≥70% of nights with residual AHI <10) (n=31) | Clinic | ESS | - Despite self-reported symptoms, most patients with OSA were not objectively sleepy, as determined by in-lab PSG followed by MSLT. |
| Hazumi M, et al. Development and validation of the “Scale of Severity of Sleepiness-specific Distress: SSSD.” *Sleep Biol Rhythms*. 2018; 16(4):497. [Abstract] | Cross-sectional psychometric validation study | Patients with suspected hypersomnia (n=93) | Clinic | SSSD | - The SSSD has construct validity and internal consistency; the “self-consciousness about somnolence” component predicts mental health problems in patients with hypersomnia. |
| Kurtis MM, et al. A review of scales to evaluate sleep disturbances in movement disorders. *Front Neurol*. 2018;9:369. | Literature review | N/A | N/A | SSS, ESS, ISCS, SCOPA-Sleep-DS | - The SSS is “suggested” for rating sleepiness and measuring severity at a specific point in time in patients with PD and sleep disturbances; a limitation is the lack of data on the psychometric characteristics of the SSS. - The ESS is recommended for screening and evaluating DS severity in patients with PD, and has shown sensitivity to change after intervention. However, it does not include: - Data from an outside source (eg, a partner), without which risk may be underestimated. - A risk of falling asleep while driving item. - A risk of sudden sleep attacks item. - The ISCS is “suggested” for evaluating sleep attacks and DS and use in conjunction with the ESS is recommended. There is no published psychometric analysis for ISCS; terminology may lead to confounders because “sudden blank spells” may be owed to sleep attacks, syncope, or partial seizures. - The SCOPA-Sleep-DS subscale is recommended for screening and measuring DS severity and sleep attacks in PD, but sensitivity to change has not been investigated. |
| Lapin BR, et al. The Epworth Sleepiness Scale: validation of one-dimensional factor structure in a large clinical sample. *J Clin Sleep Med.* 2018;14(8):1293-1301. | Retrospective cohort study | Patients presenting to a sleep clinic for the first time (n=10,785) | Clinic | ESS | - This study confirmed the unidimensionality of the ESS in a large population. - The ESS provides an accurate measure of the construct of EDS whether within patients with severe sleep apnea or in a population of young healthy respondents. |
| Li Y, et al. The clinical utility of subjective vs. objective tests of excessive daytime sleepiness in the assessment of patients with sleep apnoea. *J Sleep Res.* 2018;27(suppl 2):e12765. [Abstract] | Cross-sectional study | Patients with OSA (n=58) | In-laboratory | ESS, MSLT, PVT | - MSLT is associated with low-grade inflammation and ESS with impaired sustained attention/ vigilance (as measured by PVT) in patients with OSA. - MSLT predicts CV morbidity, whereas ESS is a good predictor for impaired performance. |
| Plante DT, et al. Multimodal hypersomnolence assessment substantially increases objective identification of hypersomnolence in patients referred for multiple sleep latency testing. *Sleep*. 2018; 41(suppl):A227-A228. [Abstract] | Psychometric validation study | Patients referred for PSG and MSLT (n=100) | Clinic | MSLT and MHA  [Objective hypersomnolence on MHA defined as ≥ 1 of: MSLT mean sleep latency ≤8 min, mean PUI ≥9.8, mean PVT lapses ≥4.8, or total sleep duration (ad libitum PSG plus MSLT naps) ≥11 h] | - MSLT detected 26 patients who were objectively sleepy, while the MHA detected an additional 36 patients as hypersomnolent. - In patients referred for PSG/MSLT, MHA more than doubled the proportion of patients who were found to have objective hypersomnolence, indicating that hypersomnolence assessment should expand beyond MSLT. |
| Cameron K, et al. Validation of the Flinders Fatigue Scale as a measure of daytime fatigue. *Sleep Med.* 2017;30:105-112. | Case-control study | Community volunteers (n=439), respondents with CFS/ME (n=66), untreated adults with insomnia (n=292), and untreated adults with OSA (AHI ≥15; n=132) | Online | FFS | - FFS showed satisfactory internal consistency; convergent validity was confirmed by significant correlations with the Fatigue Severity Scale. - FFS showed adequate discriminant validity in clinically fatigued populations, with nonsignificant correlations with ESS. - FFS showed sensitivity to fatigue variances between diagnostic subgroups. - Suggested cutoff scores were: 13 (borderline; scores ≥13 reflect an “average” level of fatigue in patients with insomnia), 16 (moderate; maximally discriminates people with insomnia from a nonclinical population), 21 (high; maximally discriminates people with CFS/ME from a nonclinical population; scores ≥21 reflect “average” level of fatigue in patients with CFS/ME). |
| Dauvilliers Y, et al. Measurement of narcolepsy symptoms: the Narcolepsy Severity Scale. *Neurology.* 2017;88(14):1358-1365. | Psychometric validation study | Untreated (n=70) and treated (n=105, taking meds to treat EDS or cataplexy) adults with NT1 | Clinic | NSS | - The NSS has internal consistency, content validity, reproducibility, and responsiveness to medication, indicating that it is a valid, reliable, and informative tool to assess symptom severity and detect changes on treatment. - NSS measures all clinical narcolepsy symptoms (EDS, cataplexy, hallucinations, sleep paralysis, disturbed nighttime sleep). |
| Evans KA, et al. Screening commercial vehicle drivers for obstructive sleep apnea. *Workplace Health Saf*. 2017;65(10):487-492. | Quality improvement project | Commercial vehicle drivers who presented for their DOT physical exams (N=382) | Clinic | STOP-Bang Questionnaire, OSAEW | - 44% (n=161) of all drivers screened were positive on the STOP-Bang, the OSAEW, or both.   - 23 had known OSA   - 2 had previous negative sleep studies   - 136 were untested/undiagnosed; of these only 50 were referred for sleep studies, 12 of whom completed sleep studies (9 of 12 were positive for OSA) - HCPs were reluctant to refer high-risk commercial drivers for sleep studies and drivers were reluctant to cooperate with sleep testing. |
| Gallais B, et al. Further evidence for the reliability and validity of the Fatigue and Daytime Sleepiness Scale. *J Neurol*. 2017;375:23-26. | Psychometric validation study | Adult participants with DM1 (molecular confirmation and late onset or adult phenotype) (n=48) | Clinic | FDSS | - The FDSS showed moderate/acceptable internal consistency (Cronbach’s alpha=0.60) and good intra-rater reliability (ICC=0.83). - Participants with complaints of both fatigue and daytime sleepiness had higher FDSS scores than those without (*P*=0.01). - FDSS is reliable and valid for patients with DM1. |
| Hosokawa K, et al. Comparison of sleep latency measured by the Oxford Sleep Resistance Test and simultaneous EEG in Japanese patients. *Sleep Med*. 2017; 40(suppl 1):e134-e135. [Abstract] | Psychometric validation study | Males with OSA (n=17) | Clinic | OSLER test and EEG | - A significant positive correlation was observed between the OSLER and EEG (*P*<0.0001, r=0.963). |
| Kallweit U, et al. Patient-reported measures of narcolepsy: the need for better assessment. *J Clin Sleep Med*. 2017; 13(5):737-744. | Literature review | Patients with OSA and/or insomnia or narcolepsy (n=35 studies) | N/A | ESS, UNS, SSI, CETQ, NSSQ, NASQ | - For the ESS, internal consistency was good (Cronbach’s alpha, 0.73–0.86), although pooled correlations with other constructs varied from moderate (MWT [rho, −0.43]) to weak (MSLT [rho, −0.27] and OSA-related variables [rho, 0.11–0.23]); evidence for test-retest reliability was lacking. - The ESS (score ≥14) had a sensitivity of 91% and specificity of 54%. - The UNS, SSI, CETQ, NSSQ, and NASQ were evaluated as narcolepsy screening tools |
| Lehert P. Epworth Sleepiness Scale ESS: determination of a minimum clinically relevant difference. *Sleep Med.* 2017; 40(suppl 1):e186-e187. [Abstract] | Psychometric analysis | Patients with narcolepsy who participated in trials of pitolisant (n=531) | 3 RCTs and 1 open-label trial | ESS | - The minimally clinically relevant difference on the ESS was determined to be a change of ±3 points, which corresponded with the perceived change of the Clinical Global Impression of the patients. |
| Putilov AA, et al. Reliability and external validity of the six scales of 72-item Sleep-Wake Pattern Assessment Questionnaire (SWPAQ). *Biol Rhythm Res*. 2017; 48(2):275-285. | Psychometric validation study | Russian residents (n=160) | N/A | SWPAQ | - Depending on the subsamples, reliability of each of 6 SWPAQ’s scales was good or acceptable. - Scores on any of the 6 scales were significantly predicted by behavior and habitual measures of sleep-wake cyclicity, such as sleep latency, sleep timing, and sleepiness after sleepless night. |
| Marques DR, et al. The DSPS-4: a brief measure of perceived daytime sleepiness. *J Sleep Res.* 2016; 25(suppl 1):156-157. [Abstract] | Psychometric validation study | Undergraduate medical students (n=344) | N/A | DSPS-4 | - The DSPS-4 demonstrated good reliability (α=0.72) and validity. - DSPS-4 appears to be useful in routinely assessing perceived daytime sleepiness. |
| Onen F, et al. A three-item instrument for measuring daytime sleepiness: the Observation and Interview Based Diurnal Sleepiness Inventory (ODSI). *J Clin Sleep Med*. 2016; 12(4):505-512. | Psychometric validation study | Elderly patients with OSA (n=73) and controls (n=60) | Clinic and community | ODSI | - Mean ODSI scores were significantly higher in older patients with OSAS (8.4±5.2) compared with healthy older controls (2.6±1.5; *P*<0.001). - ODSI has acceptable internal consistency (reliability), validity, and high test-retest reliability properties. - ODSI has acceptable correlation with the ESS (Pearson r=0.697). - Receiver operating characteristic analysis indicated that a cutoff point of 6 is successful for identifying older adults who have EDS. |
| Rosenthal L. Assessment of sleepiness: are these scales useful? *Sleep*. 2016;39(suppl): A368. [Abstract] | Psychometric validation study | Sleep medicine clinic patients (n=46) | Clinic | ESS and ToDSS | - ESS and ToDSS provided acceptable sensitivity in identifying waketime sleepiness; however, these scales misidentified 10% of patients with a false-negative assessment of subjective sleepiness. |
| Guaita M, et al. The Barcelona Sleepiness Index: a new instrument to assess excessive daytime sleepiness in sleep disordered breathing. *J Clin Sleep Med*. 2015;11(11):1289-1298. | Psychometric validation study | Patients complaining of snoring with and without EDS (n=98) | Clinic | BSI | - BSI is highly correlated with objective assessments of sleepiness and is sufficiently sensitive to change post-CPAP in patients with SDB. |
| Van der Heide A, et al. Comparing treatment effect measurements in narcolepsy: the Sustained Attention to Response Task, Epworth Sleepiness Scale, and Maintenance of Wakefulness Test. *Sleep*. 2015;38(7):1051-1058. | Psychometric analysis | Patients with narcolepsy who participated in a study of pitolisant (n=95) | RCT | ESS, SART, and MWT | - SART is useful in measuring treatment efficacy in narcolepsy, with a reliability of >0.8. - Using SART and ESS in combination includes a complete assessment of treatment effects in narcolepsy, as ESS is a subjective evaluation of how sleepy patients feel while SART is objective. |
| Alakuijala A, et al. The Oxford Sleep Resistance test (OSLER) and the Multiple Unprepared Reaction Time Test (MURT) detect vigilance modifications in sleep apnea patients. *J Clin Sleep Med*. 2014; 10(10): 1075-1082. | Psychometric validation study | Patients with suspected OSA (n=192) and healthy adult controls (n=34) | Clinic | OSLER and MURT | - Reaction times <250 msec and <10 invalid hits per test in the MURT with the OSLER-2 device are considered normal. - The OSLER and MURT tests are practical and reliable tools for measuring improvement in vigilance due to CPAP therapy in professional drivers with OSA. |
| Geiger Brown J, et al. Measuring subjective sleepiness at work in hospital nurses: validation of a modified delivery format of the Karolinska Sleepiness Scale. *Sleep Breath.* 2014;18(4):731-739. | Psychometric validation study | Hospital nurses (n=40) | Community | KSS, ESS, PVT | - High KSS scores (KSS=9) were associated with longer reaction times on PVT. - Higher sleepiness was found in nurses working night shift (on the third sequential night compared to the first, among those with sleep disorder symptoms ([especially insomnia]), and in those with trait sleepiness on ESS. |
| Kendzerska TB, et al. Evaluation of the measurement properties of the Epworth Sleepiness Scale: a systematic review. *Sleep Med Rev*. 2014;18(4):321-331. | Psychometric validation study; literature review | 35 studies | N/A | ESS | - There is moderate evidence to support test-retest reliability of the ESS. - There is strong evidence that not all ESS items belong to 1 underlying dimension. - Cronbach’s alpha=0.7–0.9, demonstrating internal consistency for within- or between-group comparisons. - Larger correlations of ESS were found with other measures of daytime sleepiness, such as the MWT and MSLT vs severity of OSA and general health measures. |
| Kendzerska T, et al. How does the Epworth Sleepiness Scale measure the underlying continuum of daytime sleepiness? Item response theory models. *Am J Respir Crit Care Med*. 2013;187: A3454. [Abstract] | Psychometric validation study | Adults who underwent a sleep study and patients with symptomatic osteoarthritis | N/A | ESS | - ESS has high reliability (rho: 0.84–0.85). - ESS is a unidimensional cumulative scale that meets minimal requirements of the item response theory models. |
| Apolonio J. Comparison of Berlin, STOP, and STOP-Bang questionnaires as screening tool in adult patients with obstructive sleep apnea. *Respirology*. 2011;16(suppl 2):242-243. [Abstract] | Psychometric validation study | Patients referred or admitted for evaluation of OSA (N=88) | Hospital | Berlin, STOP-Bang questionnaires | - Berlin is better at identifying patients who are low-risk for OSA; STOP-Bang is more sensitive and specific in detecting patients who are high-risk for OSA. - If a patient is at risk for OSA with Berlin, STOP-Bang should then be utilized to identify whether the risk of OSA is moderate or high. |
| Yu L, et al. Development of short forms from the PROMIS sleep disturbance and Sleep-Related Impairment item banks. *Behav Sleep Med*. 2011;10(1):6-24. | Psychometric validation study | Patients with various health conditions (N=2252) individuals | Clinic; online | PROMIS SD and SRI item banks, SD and SRI short forms, PSQI, and ESS | - There were larger product-moment correlations between SD and PSQI than between SRI and ESS, supporting the validity of the SD and SRI full banks and 8-item short forms. - SRI Ɵ values and the 8-item short form and full bank correlated more strongly with PSQI than ESS, although correlations with the ESS were larger for SRI than for SD. - SD and SRI 8-item form have construct validity. - PROMIS SD and SRI will be most appropriate for clinical and research applications requiring unidimensional severity scales; they are not appropriate in obtaining quantitative sleep estimates. |
| Buettner A, et al. A measuring instrument of daytime sleepiness. *J Sleep Res*. 2010; 19(suppl 2):303-304. [Abstract] | Psychometric validation study | Patients with OSA (N=45) | Clinic-based | ESS, Mackworth-Clock (Q&M), sustained attention test CARDA, pupillography, the Read Test | - Read and Q&M had the highest sensitivity and specificity. - ESS showed congruity with the Q&M and Read tests; both instruments are good for measuring objective daytime sleepiness. |
| Shahid A, et al. Measurements of sleepiness and fatigue. *Psychosom Res*. 2010; 69(1):81-89. | Psychometric validation study | N/A | N/A | MSLT, MWT, SSS, KSS, ESS, SWAI, DSS, and NSOS | - MSLT test-retest reliability was good to excellent (r values = 0.65–0.97). - With a 5-min cutoff, MSLT sensitivity and specificity were 80.9% and 89.8%, respectively; 94.5% and 73.3%, respectively, with a cutoff of 8 min; and 52% and 98.3%, respectively, when cutoff was ≤3 min. - MSLT is reliable, valid, and accurate, but may fail to assess some aspects of sleepiness. - When MWT cutoff was set at 12 min, sensitivity was 84.3% and specificity was 98.4%. - Mean SSS ratings positively correlated (r=0.68) with those of the Wilkinson test, which measures performance after a 2-hr reduction in sleep time. - The KSS can be useful to assess changes in response to environmental factors, circadian rhythm, and effects of drugs; it has not been used widely for clinical purposes because it does not measure trait sleepiness. - ESS test-retest reliability was high (Pearson correlation between 2 scores during 5-mo period = 0.822). - ESS has high internal consistency (Cronbach’s alpha = 0.88). - SWAI has high internal consistency for the factor of EDS (Cronbach’s alpha = 0.89). - MSLT was used to validate SWAI, and the EDS factor of SWAI showed a high predictive value of mean MSLT scores. - The revised 8-item DSS has good internal consistency (Cronbach’s alpha = 0.71), and acceptable construct validity. - NSOS has a reasonable internal consistency (Cronbach’s alpha = 0.58). |
| Marin HA, et al. State Trait Sleepiness Inventory, a new instrument for measuring daytime sleepiness. *Sleep*. 2009;32(suppl):A383.  [Abstract] | Psychometric validation study | University students (N=451); individuals with snoring symptoms were excluded | N/A | STSS | - Cronbach’s alpha for trait sleepiness was 0.72 and 0.70 for state sleepiness, showing internal consistency. |
| Martinez-Martin P, et al. SCOPA-Sleep and PDSS: two scales for assessment of sleep disorder in Parkinson’s Disease. *Mov Disord*. 2008;23(12):1681-1688. | Psychometric validation study | 187 patients with idiopathic PD | N/A | PDSS and SCOPA-Sleep | - Cronbach’s alpha reached values apt for group for both scales, but not for individual comparisons. |
| Maldonado CC, et al. A pictorial sleepiness scale based on cartoon faces. *Sleep*. 2004;27(3):541-548. | Psychometric validation study | Patients from diagnostic sleep laboratory and healthy university students (N=835) | N/A | PSS | - PSS can be useful in illiterate or non-English-speaking workers and children; it is also simple, quick, and inexpensive. - PSS can be used to measure self-reported sleepiness in children as young as 4 y. - PSS can be used for epidemiologic studies or fieldwork. |
| Weaver TE. Outcome measurement in sleep medicine practice and research. Part I: assessment of symptoms, subjective and objective daytime sleepiness, health-related quality of life and functional status. *Sleep Med Rev*. 2001; 5(2):103-128. | Review | N/A | N/A | SSS, KSS, ESS, SWAI, IDS of the SSSA, Rotterdam Daytime Sleepiness Scale, MSLT, MWT, OLSER | - SSS is positively correlated with PSG and MSLT sleep latency. - SSS is negatively correlated with PSG and MSLT sleep efficiency and total sleep time. - SSS has been valuable in documenting immediate treatment outcomes. - KSS related well to physiological sleepiness and responses on performance tasks. - SWAI has moderate to high internal consistency. - SWAI predicted and discriminated EDS based on MWT mean sleep latency, demonstrating construct validity. - ESS has a Cronbach’s alpha >0.70, showing internal consistency suitable for research and practice. - IDS had high test-retest reliability and internal consistency that make it appropriate for use in research; the magnitude of change in IDS scores was consistent with those measured by the SSS and ESS, showing construct validity. - RDSS was not related to affective psychological scales, but was moderately associated with the Fatigue and Vigor subscale of the Profile of Mood States and weakly correlated with the Somatic Scale of the Hopkins Symptom Checklist. - MSLT has construct validity and can distinguish between individuals with and without pathological EDS, as demonstrated by changes in sleep latency after prolonged partial sleep deprivation. - The clinical utility and application of the MSLT in OSA treatment remains unresolved. - MWT can be utilized to measure ability to drive or work. - OSLER successfully differentiated normal subjects from those with OSA and has reproducibility comparable to MWT. |
| Van Knippenberg FCE, et al. The Rotterdam Daytime Sleepiness Scale: a new daytime sleepiness scale. *Psychol Rep*. 1995;76(1):83-87. | Psychometric validation study | Patients referred for excessive snoring or suspected sleep apnea syndrome (N=96) | Home | Rotterdam Daytime Sleepiness Scale (RDSS) | - Global evaluation of daytime sleepiness correlated significantly with behavioral impact (Spearman rho=0.73, *P*<0.001) and affected life domains (rho=0.61, *P*<0.001). - Scores on each subscale were correlated with physical experience scales (eg, more fatigue, less vigor, and more somatic complaints), supporting convergent validity. - No correlations between RDSS and affective psychological scales, supporting discriminant validity. |
| Goldstein C. Current and future roles of consumer sleep technologies in sleep medicine. *Sleep Med Clin*. 2020;15(3):391-408. | Non-comprehensive review | N/A | N/A | CST | - For a CST to be considered an acceptable method to measure sleep, the algorithm output must be compared against a scored PSG. - CSTs may overestimate total sleep time and underestimate wakefulness compared with PSG. |
| Grewe FA, et al. Low repeatability of the Epworth Sleepiness Scale after short intervals in a sleep clinic population. *J Clin Sleep Med*. 2020;16(5):757-764. | Psychometric validation study (repeatability) | Patients with suspected OSA (N=40) | Clinic-based | ESS | - ESS scores varied within the same day and same week. - Between sequential ESS scores, scores varied by ≥2 points in 63% of participants, by ≥3 points in 48%, by ≥5 points in 20%, and by ≥7 points in 8%. - This study found insufficient test-retest reliability of the ESS even when retesting within 1 day. |
| Taylor E, et al. The reliability of the Epworth Sleepiness Score in a sleep clinic population. *J Sleep Res*. 2019;28(2): e12687. | Retrospective review; psychometric validation study (test-retest reliability) | Sleep clinic patients (N=133) | Clinic-based | ESS | - ESS test-retest reliability was poor when scores were obtained in different healthcare settings separated by long periods of time (ie, median of 91 days between primary care [GP] ESS and the first secondary care [Oximetry] ESS). |
| Campbell AJ, et al. Clinical reproducibility of the Epworth Sleepiness Scale for patients with suspected sleep apnea. *J Clin Sleep Med*. 2018;14(5):791-795. | Retrospective psychometric validation study | Patients referred for sleep study assessment (N=154) | Clinic-based | ESS | - An ESS score difference of ≥3, ≥5, and ≥7 occurred in 46%, 21%, and 8% of patients, respectively. |
| Khosla S, et al. Consumer sleep technology: an American Academy of Sleep Medicine position statement. *J Clin Sleep Med*. 2018;14(5):877-880. | Position statement | N/A | N/A | CST | - CST must be FDA cleared and rigorously tested against current gold standards if intended to render diagnosis and/or treatment. - Given the unknown potential of CST to measure sleep or assess for sleep disorders, these tools are not substitutes for evaluation, but can enhance patient-doctor interaction. |
| Bonzelaar LB, et al. Validity of the Epworth Sleepiness Scale as a screening tool of obstructive sleep apnea. *Laryngoscope*. 2017; 127(2):525-531. | Retrospective chart review | Patients evaluated at otolaryngology clinic (N=100) | Clinic-based | ESS | - Both subject and partner ESS scores had significantly weak/moderate positive correlations with AHI (*P*=0.34 and *P*=0.36, respectively). - ESS is a subjective scale and may be vulnerable to bias, falsification, or lack of comprehension. |
| Thomann J, et al. Psychomotor vigilance task demonstrates impaired vigilance in disorders with excessive daytime sleepiness. *J Clin Sleep Med*. 2014; 10(9):1019-1024. | Psychometric validation study | Patients referred to sleep laboratory (N=143) and healthy controls (N=67) | Clinic-based | Psychomotor vigilance task (PVT) | - PVT demonstrates different patterns between healthy subjects and those who have sleep-wake disorders, as well as between patients with different sleep-wake disorders. - Patients with narcolepsy or hypersomnia were more likely to have abnormal PVT reaction times compared with patients with Insufficient Sleep Syndrome. - Age significantly influences PVT in healthy subjects, but not in those with sleep-wake disorders. - PVT may aid in diagnosis of sleep-wake disorders. |
| Arand D, et al. The clinical use of the MSLT and MWT. *Sleep.* 2005;28(1): 123-144. | Literature review | N/A | N/A | MSLT, MWT | - MSLT correlates with subjective reports, varies with prior total sleep time, and responds to the effects of sedating/alerting agents, demonstrating face value validity as a quantification of sleepiness. - In studies of patients with OSA, the mean sleep latency (MSL) from the MWT and MSLT overlapped extensively with those of normal control subjects. - MSL from 20-min MWT provided best separation between groups, followed by 40-min MWT. - MSL on both the MSLT and MWT does not differentiate well between patients with sleep disorders and normal populations due to large standard deviation and floor/ceiling effects. |
| Littner MR, et al. Practice parameters for clinical use of the multiple sleep latency test and the maintenance of wakefulness test. *Sleep*. 2005;28(1): 113-121. | Practice parameter guidelines | N/A | N/A | MSLT, MWT | - MSLT shows excellent interrater and intrarater reliability for sleep latency assessments and REM onset scores in a sleep-disordered population. - For both MSLT and MWT, findings are most valuable when paired with clinical history, patient adherence with treatment, and when the test is performed while the patient is on their usual sleep/wake schedule. - MWT 40-min protocol can be utilized to measure a person’s capacity to stay awake when their incapacity to stay awake represents a public or personal safety issue. - MWT can be used in patients with EDS to assess treatment response. |
| Le Grande MR, et al. Diagnostic accuracy and suitability of instruments that screen for obstructive sleep apnoea, insomnia and sleep quality in cardiac patients: a meta-analysis. *Sleep Med*. 2021;86:135-160. | Meta-analysis | Patients with cardiac disorders | N/A | BQ, STOP, STOP-Bang, NoSAS score, Mallampati score, Sleep Apnea Scale of Sleep Disorders Questionnaire, ESS, SSS, FOSQ, FOSQ-10, PSQI, Verran and Snyder-Halpern Visual Analogue Sleep Scale, Richards-Campbell Sleep Questionnaire, Uppsala Sleep Inventory, Uppsala Sleep Inventory – Chronic Heart Failure, Jenkins Sleep Scale | - BQ and STOP-Bang revealed acceptable sensitivity but poor specificity. - Specificity of STOP-Bang may be increased by increasing the cut-off value from the standard score of ≥3. - Using a combination of scores, such as ESS and STOP-Bang, has been shown to increase specificity or sensitivity, although it is thought that the ESS has poor diagnostic accuracy. - A combination of FOSQ-10 and STOP-Bang may maximize predictive ability while keeping the assessment practical. |
| Benitez I, et al. Validation of the Satisfaction, Alertness, Timing, Efficiency and  Duration (SATED) questionnaire for sleep health measurement. *Ann Am Thorac Soc*. 2020; 17(3):338-343. | Psychometric validation study | Community samples (phase 1, N=4385; phase 2, N=200) | At home | SATED questionnaire | - SATED showed adequate internal consistency (Cronbach’s α=0.77). - Criterion validity was established with correlations with ESS, PSQI, and a 1-wk sleep diary. - Construct validity was established with correlations with STAI and POMS. - Test-retest reliability showed high correlation between the answers to the test and its repetition 1 wk later. |
| Hazumi M, et al. Development and validation of the hypersomnia-specific beliefs scale. *Sleep Med*. 2020;75:256-262. | Psychometric validation study | Patients with NT1 (N=10) and idiopathic hypersomnia (N=1) | Clinic-based | HSB scale | - The authors developed the HSB scale, comprising 3 subscales: “aversion toward doze,” “hypersensitivity toward others’ reactions about my doze,” and “sense of defeat caused by doze” using qualitative and quantitative approaches. - The items on this scale were found to be specific to patients with NT1, IHS, depression, and social anxiety. |
| Hunasikatti M. Low repeatability of the Epworth Sleepiness Scale and the need to redefine the minimal clinically important difference. *J Clin Sleep Med*. 2020;16(10):1827. | Letter to the Editor | N/A | N/A | ESS | - ESS reliability is not adequate to provide the foundation for clinical decisions or to measure treatment effects because baseline fluctuation of scores reaches or exceeds the MCID. - Due to this variance, the author suggests changing the MCID from 2–3 points to 5–6 points. |

AHI, Apnea-Hypopnea Index; AUC, area under curve; BD, bipolar disorder; BMI, body mass index; BQ, Berlin Questionnaire; BSI, Barcelona Sleepiness Index; CCUQ, Cues to CPAP Use Questionnaire; CETQ, Cataplexy Emotional Trigger Questionnaire; CFS/ME, Chronic Fatigue Syndrome/Myalgic Encephalitis; CI, confidence interval; COMISA, comorbid insomnia/obstructive sleep apnea; CPAP, continuous positive airway pressure; CSESA-J, CPAP Self-Efficacy Questionnaire for Sleep Apnea in Japanese; CST, consumer sleep technology; CV, cardiovascular; DM1, myotonic dystrophy type 1; DOT, Department of Transportation; DS, daytime sleepiness; DSPS, Daytime Sleepiness Perception Scale; DSS, Daytime Sleepiness Scale; EDS, excessive daytime sleepiness; EEG, electroencephalography; ESS, Epworth Sleepiness Scale; FDA, US Food and Drug Administration; FDSS, Fatigue and Daytime Sleepiness Scale; FFS, Flinders Fatigue Scale; FOSQ-10, short version of the Functional Outcomes of Sleep Questionnaire; GP, general practitioner; GSAQ, Global Sleep Assessment Questionnaire; HCP, healthcare professional; HSB, Hypersomnia-Specifics Belief scale; HSI, Hypersomnia Severity Index; ICF, International Classification of Functioning, Disability, and Health; IDS, Index of Daytime Sleepiness; ISCS, Inappropriate Sleep Composite Score; KSS, Karolinska Sleepiness Scale; MCID, minimal clinically important difference; MDD, major depressive disorder; MHA, multimodal hypersomnolence assessment; min, minutes; MOS, Medical Outcome Study; MOSAS, Maugeri Obstructive Sleep Apnea Syndrome; MSP, Mokken Scale Procedure; MSLT, multiple sleep latency test; MURT, Multiple Unprepared Reaction Time Test; MWT, maintenance of wakefulness test; NoSAS, Neck circumference, Obesity, Snoring, Age, Sex; NSAQ, Narcolepsy Symptoms Assessment Questionnaire; NSOS, Nocturnal Sleep Onset Scale; NSS, Narcolepsy Severity Scale; NSSQ, Narcolepsy Symptom Status Questionnaire; NT1, narcolepsy type 1; ODSI, Observation and Interview Based Diurnal Sleepiness Inventory; OSA, obstructive sleep apnea; OSAEW, obstructive sleep apnea evaluation worksheet; OSAS, obstructive sleep apnea syndrome; OSLER, Oxford Sleep Resistance; PAP, positive airway pressure; PD, Parkinson’s disease; PDSS, Pediatric Daytime Sleepiness Scale; PLMD, periodic limb movement disorder; pESS, Pictorial Epworth Sleepiness Scale; POMS, Profile of Mood States; PROMIS, Patient-Reported Outcomes Measurement Information System; PSG, polysomnography; PSQI, Pittsburgh Sleep Quality Index; PSS, Pictorial Sleepiness Scale; pSSAS, Pictorial Epworth Sleepiness Scale; PVT, psychomotor vigilance task; Q&M, Quatember and Maly; QSQ, Quebec Sleep Questionnaire; RBD, REM behavior disorder; RCT, randomized controlled trial; REM, rapid eye movement; ROC, receiver operating curve; SAQLI, Sleep Apnea Quality of Life Index; SART, Sustained Attention to Response Task; SATED, Satisfaction, Alertness, Timing, Efficiency and Duration; SBQ, STOP-Bang Questionnaire; SCOPA-Sleep, Scales for Outcomes in Parkinson’s Disease Sleep; SCOPA-Sleep-DS, SCOPA-Sleep Daytime Sleepiness; SD, standard deviation or sleep disturbance (Yu 2011); SDB, sleep-disordered breathing; SEM, standard error measurement; SEMSA, The Self Efficacy Measure for Sleep Apnea; SF-36, Medical Outcomes Study 36-Item Short Form Health Survey; SNS, Swiss Narcolepsy Scale; SOS, Snore Outcomes Survey; SQS, Sleep Quality Scale; SRI, sleep-related impairment; SSS, Stanford Sleepiness Scale; SSSA, Survey Screen for Sleep Apnea; SSSD, Scale of Severity of Sleepiness-specific Distress; SSI, Stanford Sleep Inventory; STAI, State-Trait Anxiety Inventory; STOP-Bang, Snoring, Tiredness, Observed apnea, blood Pressure, Body mass index, Age, Neck circumference and Gender; STSS, State Trait Sleepiness Inventory; SWAI, Sleep Wake Activity Inventory; SWIFT, Sleepiness-Wakefulness Inability and Fatigue Test; SWPAQ, Sleep-Wake Pattern Assessment Questionnaire; ToDSS, Time of Day Sleepiness Scale; TSS, trait sleepiness scale; UNS, Ullanlinna Narcolepsy Scale; WPA, wake-promoting agent.

**Supplementary Table 6. Supporting evidence for Search 2A: Within a real-world clinical population, how do you define adequate amounts of sleep and establish whether this criterion has been met for an individual patient?**

| **Supporting evidence for Search 2A: Within a real-world clinical population, how do you define adequate amounts of sleep and establish whether this criterion has been met for an individual patient?** | | | | | |
| --- | --- | --- | --- | --- | --- |
| **Study** | **Article type/**  **Study design** | **Study population** | **Setting** | **Method used to determine optimal sleep duration** | **Definition of optimal sleep duration** |
| Song C, et al. Sleep quality and risk of cancer: findings from the English longitudinal study of aging. *Sleep.* 2021;44(3):zsaa192. | Observational, prospective, longitudinal, cohort study | English Longitudinal Study of Aging national cohort of adults ≥50 y living in England (2008–2009) (N=10,036) | Population-based | Prespecified based on previous studies | 6 to 9 h/night |
| Pérez-Tasigchana RF, et al. Combined impact of traditional and nontraditional healthy behaviors on frailty and disability: a prospective cohort study of older adults. *J Am Med Dir Assoc*. 2020;21(5): 710.e1-710.e9. | Prospective cohort study | Community-dwelling individuals ≥60 y living in Spain (N=4008) | Population-based | Prespecified in methods | 7 to 8 h/d |
| Jansen EC, et al. Associations between sleep duration and dietary quality: results from a nationally-representative survey of US adults. *Appetite*. 2020;153:104748. | Cross-sectional survey | Adults (N=12,930) | Population-based | Analysis of self-reported data collapsed into 4 categories (≤5 h/d; 6 h/d; 7–8 h/d; ≥9 h/d) | 7 to 8 h/d   - Individuals with ≤5 h and ≥9 h of sleep had worse overall dietary quality vs those with 7–8 h |
| Suzuki M, et al. Sleep disturbance is associated with not only shorter sleep duration, but also longer time in bed: a Japanese general population survey. *Biol Rhythms*. 2019;17(4): 407-415. | Cross-sectional survey using face-to-face interviews | Adults (N=2542) | Population-based | Analysis of data obtained using Japanese PSQI | > 6 h/night   - Difficulty initiating sleep, difficulty maintaining sleep, early morning awakening, and any sleep difficulty were associated with sleep duration < 6 h |
| Lallukka T, et al. Association of sleep duration and sleep quality with the physical, social, and emotional functioning among Australian adults. *Sleep Health*. 2018;4(2):194-200. | Cross-sectional study | Australia residents (N=14,571) | Population-based | Analysis of self-reported data collapsed into 3 categories (short sleep [<6 h/night] mid-range sleep [6–8 h/night], and long sleep [>8 h/night]) | 6 to 8 h/night   - Short and long sleep durations had poorer functioning vs those with 6–8 h sleep, regardless of sleep quality |
| Bayan-Bravo A, et al. Combined impact of traditional and non-traditional health behaviors on health-related quality of life: a prospective study in older adults. *PLoS One.* 2017;12(1):e0170513. | Prospective study with telephone interview | Spain residents (N=4008) | Population-based | Prespecified in methods based on public health guidelines and previous studies | 7 to 8 h/d |
| Mossavar-Rahmani Y, et al. Actigraphic sleep measures and diet quality in the Hispanic Community Health Study/Study of Latinos Sueño ancillary study. *J Sleep Res*. 2017;26(6): 739-746. | Cross-sectional ancillary study | Patients with no severe sleep disorders (AHI <50), no treatment for sleep apnea, and no diagnosis for narcolepsy (N=2189) | Population-based | Actigraphy and sleep diary analyzed continuously and in 3 categories (short [<6 h], intermediate [6 to <8 h], long [≥8 h]) | ≥6 h/night   - Short sleep duration was associated with worse dietary quality |
| Westerlund A, et al. Relationships between questionnaire ratings of sleep quality and polysomnography in healthy adults. *Behav Sleep Med*. 2016;14(2): 185-199. | Questionnaire | Patients with no complaints of sleep disturbances (N=33) | Population-based | Analysis of KSQ and home PSG data used to assess total sleep time of population (not optimal sleep duration specifically) | Median values: 443 min (KSQ) and 371 min (PSG) per night |
| Bonsen T, et al. Sleep quality and duration are related to microvascular function: the Amsterdam Growth and Health Longitudinal Study. *J Sleep Res.* 2015;24(2): 140-147. | Longitudinal study | Participants from the Amsterdam Growth and Health Longitudinal Study population (N=259) | Clinic-based | Prespecified based on previous studies | ≥7 h/night |
| Gaines J, et al. Short- and long-term sleep stability in insomniacs and healthy controls. *Sleep*. 2015;38(11): 1727-1734. | Observational short-term and prospective studies | Patients with insomnia (N=150) and normal-sleeping controls (N=151) | Clinic-based | Analysis of PSG data used to assess sleep time in this population (not specifically to define optimal duration) | Median sleep duration   - ≥425 to ≥430 min (controls) - ≥391 to ≥413 min (insomnia) |
| Vgontzas AN, et al. Unveiling the longitudinal association between short sleep duration and the incidence of obesity: the Penn State Cohort. *Int J Obese*. 2014;38(6): 825-832. | Longitudinal study | 1741 random participants in phase 2, 1395 participants in a follow-up survey | At home interviews and sleep lab | Analysis of self-reported data collapsed into 4 categories (≤5 h/night; 5–6 h/night; 6–7 h/night; ≥7 h/night) and previous studies on the association of self-reported sleep duration with incident obesity | ≥7 h/night |
| Martinez-Gomez D, et al. Combined impact of traditional and non-traditional health behaviors on mortality: a national prospective cohort study in Spanish older adults. *BMC Med*. 2013;11:47. | Prospective cohort study | Noninstitutionalized older Spanish adults (≥60 y) (N=4008) | At home interviews | Prespecified in methods | 7 to 8 h/d |
| Vgontzas AN, et al. Persistent insomnia: the role of objective sleep duration and mental health. *Sleep*. 2012; 35(1):61-68. | Representative longitudinal study | Phase 1: N=16,583 (telephone survey); phase 2: N=1741; follow-up survey: N=1395 | At home interviews and sleep lab | Analysis of PSG data and previous studies (<6 h has been associated with greater morbidity and mortality) | ≥6 h/night |
| Zhang J, et al. Relationship of sleep quantity and quality with 24-hour urinary catecholamines and salivary awakening cortisol in healthy middle-aged adults. *Sleep*. 2011; 34(2):225-233. | Prospective, cross-sectional study | Individuals without significant self-reported sleep disturbances (≥3 times/wk) (wave 1, N=228; wave 2, N=108) | Clinic-based | Analysis of self-reported data (questionnaires, sleep logs), and actigraphy (lowest quartile of sleep duration [≤340 min] defined as short sleepers | >340 min |
| Faubel R, et al. Sleep duration and health-related quality of life among older adults: a population-based cohort in Spain. *Sleep*. 2009;32(8):1059-1068. | Prospective, population-based cohort study | Noninstitutionalized older Spanish adults (≥60 y) (N=4008) | At home telephone interviews | Analysis of self-reported data | 7 or 8 h   - Sleep duration of 7 or 8 h was associated with lower number of chronic conditions and lower frequency of cognitive impairment |
| Vitiello MV, et al. Age-related sleep change: gender and estrogen effects on the subjective-objective sleep quality relationships of healthy, noncomplaining older men and women. *J Psychosom Res*. 2004;56(5):503-510. | Analysis of baseline data from previous studies | Healthy older adults (N=150) | Clinic-based | Analysis of PSG and self-reported (PSQI) data | 386.1 (5.0) min (total sleep time among individuals with PSQI scores ≤5) |
| Middelkoop HAM, et al. Subjective sleep characteristics of 1,485 males and females aged 50-93: effects of sex and age, and factors related to self-evaluated quality of sleep. *J Gerontol A Biol Sci Med Sci*. 1996;51(3):M108-115. | Epidemiological cross-sectional study | General practice center patients aged ≥50 y (N=1485) | Population-based | Analysis of self-reported data used to assess sleep time in population by decade (not specifically to define optimal duration) | Mean total sleep time: 7.2 to 8.5 h |
| Watson NF, et al. Recommended amount of sleep for a healthy adult: a joint consensus statement of the American Academy of Sleep Medicine and Sleep Research Society. *Sleep*. 2015;38(6):843-844. | Consensus statement | N/A | N/A | Published scientific evidence addressing the relationship between sleep duration and health | ≥7 h/night |

AHI, Apnea-Hypopnea Index; EEG, electroencephalogram; KSQ, Karolinska Sleep Questionnaire; min, minutes; PSG, polysomnography; PSQI, Pittsburgh Sleep Quality Index.

**Supplementary Table 7. Supporting evidence for Search 2B: Within a real-world OSA population, how do you define optimal treatment, and what are the parameters that equate to optimized CPAP as it pertains to utilization and duration on CPAP therapy?**

| **Supporting evidence for Search 2B: Within a real-world OSA population, how do you define optimal treatment, and what are the parameters that equate to optimized CPAP as it pertains to utilization and duration on CPAP therapy?** | | | | | | |
| --- | --- | --- | --- | --- | --- | --- |
| **Study** | **Article type/**  **Study design** | **Study population** | **Setting** | **CPAP use^a^** | **ESS score^a^**  **(pre- vs post-CPAP)** | **Key finding(s)** |
| Venkatnarayan K, et al. The effect of CPAP therapy on excessive daytime sleepiness and quality of life in subjective with obstructive sleep apnoea: an AB design study. *Sleep Breath.* 2020;25(3):1351-1357. | Observational, prospective, cross-sectional study | Patients recently diagnosed with and untreated for moderate to severe OSA  (18–75 y; N=92) | Clinic | 5.8 (1.1) h/night for 1 mo | Pre-CPAP: 11.5 (5.6)  Post-CPAP: 6.8 (3.2)  (*P*=0.00001) | - EDS, as assessed with the ESS, and QoL, as assessed with the SAQLI, significantly improved after 1 mo of CPAP therapy (SAQLI: pre-CPAP, 2.6 [1.3]; post-CPAP, 1.4 [0.9]; *P=*0.00001). |
| Rotty MC, et al. Mask side-effects in long‑term CPAP‑patients impact adherence and sleepiness: the InterfaceVent real‑life study. *Respir Res*. 2021;22(1):17. | Prospective, cross-sectional study | Adult patients undergoing ≥3 mo of CPAP or noninvasive ventilation (N=1484) | Home | Median CPAP use was 6.8 h/d; adherence was <4 h/d for 8.6% of patients; median duration of CPAP treatment was 4.4 y | ESS reported for post-CPAP assessment only  Median (range): 5 (3–9)  Residual EDS (ESS ≥11): 16.17% | - 99.3% of patients position mask without assistance, 94.9% were satisfied with their mask, and 90.4% considered mask comfortable. - The most frequent side effects in long-term CPAP-treated patients are patient-reported leaks, which are not predicted by CPAP device-reported data. - In long-term CPAP-treated patients, MRSEs are independently negatively associated with CPAP adherence and positively associated with sleepiness. |
| Zhang XQ, et al. Change in quality of life of OSAHS patients with minimally invasive surgery or CPAP therapy: a 2-year retrospective, single-center parallel-group study. *Curr Mol Med*. 2020;20(3):231-239. | Retrospective, single-center, parallel group study | Patients with sleep disorders (N=90) | Clinic-based | Not reported | CPAP Group  Baseline: 13.46 (4.03)  0.5 y: 6.61 (3.07)  1 y: 5.42 (3.36)  2 y: 3.50 (1.31)  Surgery Group  Baseline: 11.52 (5.37)  0.5 y: 7.19 (4.42)  1 y: 5.50 (4.81)  2 y: 7.78 (5.95)  Untreated Group  Baseline: 10.41 (5.06)  0.5 y: 8.28 (4.82)  1 y: 7.55 (5.09)  2 y: 10.70 (4.69) | - EDS, as assessed by ESS, was improved most with CPAP, followed by surgery and no treatment. |
| Walia HK, et al. Upper airway stimulation vs positive airway pressure impact on BP and sleepiness symptoms in OSA. *Chest*. 2020;157(1):173-183. | Longitudinal comparative study | Patients receiving UAS who were intolerant to/had suboptimal adherence to PAP, history of moderate to severe OSA, and observed absence of circumferential airway collapse with drug-induced sedation endoscopy (N=278); patients using PAP (N=517) | Clinic-based | 39.2 h/wk (SD 14.8) | Pre-Propensity Score Matching in PAP group: 8.5 (5.0)  Post-Propensity Score Matching in PAP group: 10.4 (4.9)  Pre-PAP: 10.4 (range: 9.5–11.2)  Post-PAP: 7.7 (range: 6.8–8.6) *(P*<0.001) | - UAS and PAP groups showed significant improvements in ESS scores, although the UAS-treated patients had significantly greater improvements in ESS score than PAP-treated patients (−3.5 vs −2.7; *P*=0.046). - Patients using PAP therapy had significantly greater improvement in diastolic BP and mean arterial pressure compared with UAS-treated patients (3.7 mm Hg [*P*<0.001] and 2.8 mm Hg [*P*=0.008], respectively). |
| Serrano Merino J, et al. Impact of positive pressure treatment of the airway on health-related quality of life in elderly patients with obstructive sleep apnea. *Biol Res Nurs*. 2018;20(4):452-461. | Prospective pre-/post- intervention study | Patients (≥65 y) with OSA (N=103) | Clinic | 6.3 (1.41) h/d | Baseline: 10.43 (4.90)  Post-CPAP (3 mo): 5.22 (3.32)  (*P*<0.01) | - ESS post-therapy scores were significantly lower than pre-therapy (difference, 5.2 [4.47] [95% CI: 4.33, 6.07]; *P*<0.001). |
| Jacobsen AR, et al. Determinants for adherence to continuous positive airway pressure therapy in obstructive sleep apnea. *PLoS One*. 2017;12(12):e0189614. | Retrospective observational study | Sleep disorder clinic patients (N=695) | Clinic | 6.1 (1.5) h/night; 78% adherence rate after a median follow-up of 3 y  Mild OSA (n=54): 5.7 (1.6) h  Moderate OSA (n=144): 6.1 (1.2) h  Severe OSA (n=288): 6.2 (1.5) h | Persistent CPAP use (baseline): 11.0 (5.0)  Nonadherent CPAP use (baseline): 8.8 (4.6) | - AHI, ESS, and smoking status were independent predictors of adherence   - Higher AHI and EDS improved adherence; smoking decreased adherence |
| Zhao YY, et al. Effect of continuous positive airway pressure treatment on health-related quality of life and sleepiness in high cardiovascular risk individuals with sleep apnea: Best Apnea Interventions for Research (BestAIR) trial. *Sleep*. 2017;40(4):zsx040. | Randomized, parallel-group clinical trial | Untreated patients with moderate to severe OSA (AHI 4% ≥10 events/h or AHI 3% ≥15 events/h; N=169) | At home, sleep diary | 6 mo of CPAP:  3.82 (2.86) h/night  12 mo of CPAP:  3.44 (2.99) h/night | Baseline: 8.0 (4.5)  Post-6 mo of CPAP: 6.2 (3.8)  Post-12 mo of CPAP: 6.0 (4.0)  (*P*=0.040) | - CPAP led to a modest but significant improvement in self-reported EDS, despite an average CPAP use of <4 h/night. - CPAP improved multiple domains of HRQoL in relatively asymptomatic patients with moderate to severe OSA at high risk of CVD. |
| Batool-Anwar S, et al. Impact of continuous positive airway pressure (CPAP) on quality of life in patients with obstructive sleep apnea (OSA). *J Sleep Res*. 2016;25(6):731-738. | Multicenter, double-blind, randomized, 2-arm, sham-controlled intention-to-treat clinical trial | Patients with a clinical diagnosis of OSA (AHI ≥10; N=845) | Clinic | 2 mo (CPAP group): 4.31 (2.9) h/night  6 mo (CPAP group): 3.69 (3.1) h/night  (*P*<0.05 vs sham) | Change in ESS score:  Men (mild, CPAP group): 2.9 (4.8)  Men (moderate, CPAP group): 1.9 (4.1)  Men (severe, CPAP group): 3.1 (4.5)  Women (mild, CPAP group): 3.2 (4.0)  Women (moderate, CPAP group): 3.1 (4.3)  Women (severe, CPAP group): 3.5 (3.9) | - Significant ESS improvement was observed among the CPAP group (*P* <0.05 vs sham) in patients with moderate or severe OSA, particularly in women. - No significant ESS change was observed in the mild OSA cohort in either treatment arm. - In patients with severe OSA, ≥4 h/night of CPAP use enhances QoL as assessed by SAQLI. |
| Bjornsdottir E, et al. Quality of life among untreated sleep apnea patients compared with the general population and changes after treatment with positive airway pressure. *J Sleep Res.* 2015;24(3):328-338. | Prospective interventional study | Patients diagnosed with moderate to severe OSA (AHI ≥15; N=655) | Clinic | Full users: PAP used ≥20 days and ≥4 h/d on average for previous 4 wks (device data) or ≥5 nights/wk for ≥60% of the night (questionnaire data)  Non-users: returned PAP device within 1 year of therapy initiation (and no other intervention) | Baseline: 11.7 (5.1)  (post-CPAP scores not reported) | - Untreated patients with OSA showed diminished physical and mental QoL in comparison with a general population sample. - No significant differences were found in improvement of physical and mental QoL between full and nonusers of PAP from baseline to follow-up. |
| Avlonitou E, et al. Adherence to CPAP therapy improves quality of life and reduces symptoms among obstructive sleep apnea syndrome patients. *Sleep Breath*. 2012;16(2):563-569. | Interventional study | Patients referred for the evaluation of reported snoring and daytime sleepiness (N=50) | Clinic | 4.5 (0.5) h/night | Baseline: 13.7 (6.5)  Post-6 mo CPAP: 3.9 (3.8)  (*P*<0.001) | - Significant improvement in EDS and QoL were seen after 6 mo of CPAP treatment. |
| Holley AB, et al. Efficacy of an adjustable oral appliance and comparison with continuous positive airway pressure for the treatment of obstructive sleep apnea syndrome. *Chest*. 2011;140(6):1511-1516. | Retrospective study | Patients diagnosed with OSA and treated with an aOA (N=497) | Clinic and at home | N/A | Baseline: 13.0 (5.0)  Follow-up: 10.4 (5.3)  (*P*<0.001) | - Most patients using an aOA attained AHI <5 on PSG (with aOA titration); there was a significant decrease in ESS scores with aOA treatment. |
| Otake M, et al. Monitoring sleep-wake rhythm with actigraphy in patients on continuous positive airway pressure therapy. *Respiration*. 2011;82(2):136-141. | Prospective interventional study | Patients diagnosed with OSA (N=18) | Clinic | Not reported | Not evaluated | - CPAP significantly improved sleep efficiency (assessed via actigraphy) compared with the baseline night. - During the CPAP night, total sleep fragmentation and sleep fragmentation >5 min were significantly lower compared with baseline. |
| Antic NA, et al. The effect of CPAP in normalizing daytime sleepiness, quality of life, and  neurocognitive function in patients with moderate to severe OSA. *Sleep*. 2011;34(1):111-119. | Analysis of data collected as part of a randomized, controlled, open-label clinical trial | Sleep clinic patients (18–75 y) with ESS score ≥8, snoring “most” or “every” night, who were willing to try CPAP (N=174) | Clinic | Average nightly adherence categorized as: ≤2 h; >2 to <4 h; ≥4 to <5 h; ≥5 to <6 h; ≥6 to <7 h; ≥7 h | Baseline: 13.4 (4.0)   - ESS showed substantial and dose-dependent improvement following CPAP (*P*<0.001) | - Across the whole cohort, 59.5% had a normal ESS score post-treatment; among those who used CPAP ≥7 h/night, 80.6% had normal post-treatment ESS scores. - In participants with moderate to severe OSA, neurobehavioral responses to CPAP differed markedly based on the test used to assess the response. - There was a dose-response improvement in some, but not all, neurobehavioral measures; however, a substantial proportion of patients did not achieve normal functional status after 3 mo of optimal CPAP treatment (≥7 h/night). |
| Rosenberg R, Doghramji P. Optimal treatment of obstructive sleep apnea and excessive sleepiness. *Adv Ther*. 2009;26(3):295-312. | Literature review | N/A | N/A | N/A | N/A | - Many studies assume patients will need ≥4 h/night of CPAP therapy to achieve any benefit; therefore, use 4 h/night of CPAP therapy as a threshold for patient adherence. Studies defining adherence in this way report 29% to 83% of patients as nonadherent. - Successfully treating OSA and related EDS includes encouraging patients to adhere to CPAP treatment or oral appliances through simple interventions and patient support. |
| Siccoli MM, et al. Effects of continuous positive airway pressure on quality of life in patients with moderate to severe obstructive sleep apnea: data from a randomized controlled trial. *Sleep*. 2008;31(11):1551-1558. | Randomized, double-blind, controlled clinical trial | Patients with possible OSA and EDS (ESS ≥10) (N=102; sham CPAP, n=51; CPAP, n=51) | Clinic-based | CPAP group:  4.7 (2.1) h/night  Sham CPAP:  3.9 (2.5) h/night | CPAP group  Baseline: 15.8 (4.0)  Follow-up: 6.8 (5.1)  Sham CPAP  Baseline: 15.2 (4.0)  Follow-up: 11.9 (5.9)  (*P*<0.0001 CPAP vs sham) | - CPAP was superior to sham in most tested outcome measures of health status, including ESS. - ESS predicted CPAP response. |
| Loredo JS, et al. Effect of continuous positive airway pressure versus supplemental oxygen on sleep quality in obstructive sleep apnea: a placebo-CPAP–controlled study. *Sleep*. 2006;9(4):564-571. | Randomized, double-blind, placebo-controlled clinical trial | Patients with history of chronic loud snoring with or without EDS (N=63) | At home or sleep laboratory (dependent on OSA severity) | 6.61 (1.19) h for CPAP group | Baseline: 11.6 (4.9)  Post-2 wk CPAP: 8.2 (4.4) | - CPAP was not any more effective in decreasing ESS scores than placebo or supplemental oxygen after 2 wks of treatment. - Compared with placebo, CPAP was linked to swift improvement in sleep quality by decreasing sleep-stage shifts, reducing stage 1 sleep, and improving REM sleep, which continued over 2 wks of treatment. - CPAP also improved respiratory and arousal abnormalities characteristic of patients with OSA. |
| Hida W, et al. Nasal continuous positive airway pressure improves quality of life in obesity hypoventilation syndrome. *Sleep Breath*. 2003;7(1):3-12. | Interventional study | Patients with obesity and OSA (N=38) and patients of normal weight with OSA (N=48) | Clinic-based | 3 to 6 mo treatment (nightly use not reported) | Baseline (obese OSA): 12.5 (4.6) (*P*<0.01)  Baseline (nonobese OSA): 10.6 (3.8)  Follow-up: mean ESS score was <10 in both groups | - Nasal therapy improves sleep quality and sleep-disordered breathing, improves alertness, and eases EDS, which results in increased activity during the day and physical mobility as well as improvements in ability to think, emotional reactions, and QoL. |
| Beninati W, et al. Optimal continuous positive airway pressure for the treatment of obstructive sleep apnea/ hypopnea. *Sleep Med Rev.* 2001;5(1):7-23. | Review | N/A | N/A | N/A | N/A | - Literature supports recommending nightly CPAP use for the whole night or as much of the night as tolerated. |
| Akashiba T, et al. Optimal continuous positive airway pressure in patients with obstructive sleep apnoea: role of craniofacial structure. *Respir Med*. 2001;95(5): 393-397. | Interventional study with controls | Japanese men with OSA (N=27) | Clinic-based | Adherence/nightly use not reported (focus is optimizing pressure) | Not assessed | - The ideal CPAP level (ie, pressure) depended on oxygen desaturation during sleep and craniofacial structure. |
| Oh A, et al. What is a clinically meaningful target for positive airway pressure adherence? *Sleep Med Clin*. 2021;16(1):1-10. | Literature review | N/A | N/A | N/A | N/A | Studies suggest target nightly PAP usage to improve the following in patients with moderate to severe OSA:   - Daytime sleepiness: ≥4 to 5 h - Quality of life is ≥4 to 7 h - Neurocognitive outcomes: ≥6 h - Depression: ≥3.5 to 5 h - Hypertension (particularly, resistant hypertension): ≥4 to 5.5 h |
| Sanner BM, et al. Long-term treatment with continuous positive airway pressure improves quality of life in obstructive sleep apnoea syndrome. *Eur Respir J*. 2000;16(1): 118-122. | Prospective study | Patients with OSA (AHI ≥10), ESS >10, and willingness to try CPAP (N=39) | Clinic-based | 5.9 (1.1) h/night after 9 mo | Not assessed | - CPAP treatment was associated with significant improvement in several QoL measures, although correlations between CPAP adherence and change in QoL measures was not significant. - Long-term CPAP therapy improves pathological breathing patterns as well as factors that indicate QoL in those with OSAS. |
| Schöbel C, et al. Improved follow-up by peripheral arterial tonometry in CPAP-treated patients with obstructive sleep apnea and persistent excessive daytime sleepiness. *Sleep Breath.* 2018;22(4): 1153-1160. | Monocentric, prospective, randomized crossover pilot trial | Patients with OSA and sufficient CPAP treatment according to device-derived data (N=49) | Clinic-based | Eligibility: CPAP use for ≥12 mo and mean use >4 h/night  Mean CPAP treatment duration: 81 (63) months | Baseline: 8.4 (4.2)  No post-baseline assessment | - Peripheral arterial tonometry detected higher residual SDB rates in CPAP-treated patients with residual EDS, while CPAP device data and 6-channel portable monitoring showed no evidence of insufficient treatment. |
| McDaid C, et al. A systematic review of continuous positive airway pressure for obstructive sleep apnoea–hypopnoea syndrome. *Sleep Med Rev*. 2009;13(6):427-436. | Systematic literature review | N/A | N/A | N/A | N/A | - A statistically significant benefit was seen with CPAP compared with control (MD −2.7 ESS points; 95% CI: −3.5, −2.0). - Heterogeneity was high (I=71%) and was reduced when trials were subgrouped by baseline ESS. - Statistically significant improvement in EDS symptoms was seen with CPAP compared with control for all symptom severity levels, especially when baseline sleepiness was severe (MD −5.0, 95% CI: −6.5 to −3.5). - No statistically significant difference was seen on the impact on EDS (ESS) between CPAP and oral devices (MD −0.9, 95% CI: −2.1, 0.4). |
| Schwab RJ, et al. An official American Thoracic Society statement: continuous positive airway pressure adherence tracking systems. *Am J Respir Crit Care Med*. 2013;188(5): 613-620. | Official statement (American Thoracic Society) | N/A | N/A | N/A | N/A | - Adherence was defined as CPAP use >4 h/night or use >2 h/night and progressing toward improved EDS as assessed by ESS, subjective QoL improvement, or improvement of other OSA-related health issues (eg, diabetes, hypertension). - Partial use of CPAP is always better than no use, but the goal is for patients to use it full time during sleep. - Research has shown that CPAP adherence is associated with race, socioeconomic class, marital status, and psychiatric disease. - Some patients may find it hard to achieve Medicare-adherence models, thus certain population segments may potentially experience discrimination related to government-mandated reimbursement. |
| Patil SP, et al. Treatment of adult obstructive sleep apnea with positive airway pressure: an American Academy of Sleep Medicine clinical practice guideline. *J Clin Sleep Med*. 2019;15(2):335-343. | Clinical practice guideline; literature review | N/A | N/A | N/A | N/A | - Initial treatment of OSA requires close monitoring and early identification of difficulties with PAP use, as early adherence has shown to predict long-term adherence. - Yearly evaluation by a trained healthcare provider is reasonable. - It is recommended that clinicians use PAP, compared to no therapy, to treat OSA in adults with excessive sleepiness. |
| Weaver TE, et al. Relationship between hours of CPAP use and achieving normal levels of sleepiness and daily functioning. *Sleep*. 2007;30(6):711-719. | Multisite effectiveness study | Patients with OSA (AHI ≥ 15; N=149) | Clinic-based | All participants: 4.7 (2.1) h (range,  0–8.1); treatment duration: 3 mo  Participants with ESS ≤10 after CPAP: 5.1 (2.1)  Participants with ESS >10 after CPAP: 4.0 (2.3) (*P*=0.02) | Baseline (all participants): 14.7 (4.8)  Post-treatment:  Among patients with baseline ESS >10, percentage with ESS ≤10 at follow-up ranged from 41.2% with nightly CPAP use 0–2 h to 92.9% with use ≥7 h | - Patients whose sleepiness normalized after treatment (ESS ≤10) used CPAP ~1 h more/night than those whose sleepiness did not normalize. - Longer nightly CPAP durations resulted in decreased objective and subjective EDS and improved functioning to normal levels in patients who were impaired before CPAP initiation. |
| Budhiraja R, et al. Predictors of sleepiness in obstructive sleep apnoea at baseline and after 6 months of continuous positive airway pressure therapy. *Eur Respir J*. 2017;50(5):1700348. | Prospective, multicenter, RCT | Patients with OSA (AHI ≥10; N=1105; CPAP, n=558; sham CPAP, n=547) | Clinic | Mean nightly adherence at 6 mo in those randomized to active CPAP was 4.70 (2.04) h; 67.3% of participants were using CPAP >4 h/night | Baseline:  Total: 10.4 (4.4)  CPAP vs sham: 10.5 vs 10.5 (*P*=0.99)  Post-treatment (CPAP vs sham):  2 mo: 7.9 vs 8.8; *P*=0.003  4 mo: 7.0 vs 8.2; *P*<0.001  6 mo: 7.3 vs 8.4; *P*=0.003 | - CPAP use >4 h/night is linked to significantly reduced odds of sleepiness at 6 mo (OR: 0.425; *P*=0.001). - Increased CPAP adherence >4 h/night might result in additional EDS reduction although residual EDS may be due to factors other than OSA. - Lower average nightly CPAP use and presence of EDS at baseline were the primary determinants of residual EDS in patients using CPAP, even among those who are CPAP-adherent. |
| Cistulli PA, et al. Short-term CPAP adherence in obstructive sleep apnea: a big data analysis using real world data. *Sleep Med*. 2019;59:114-116. | Database analysis | Adults treated with AirSense/AirCurve 10 (ResMed Corp) PAP platform (Air10) and a single PAP modality for ≥1 hour during the first 90 days of therapy (N=2.62 million patients; 23.4% of patients were excluded) | Database analysis | Median: 93% of nights; mean use: 5.1 (2.5) h across all nights and 6.0 (2.0) h on nights used  Adherent: 75% (PAP use ≥4 h/night on 70% of nights in a consecutive 30-day period in the first 90 days of therapy) | N/A | - The short-term adherence rate of 75% in this analysis appears higher than is generally acknowledged in clinical practice and in the literature. - Treatment was efficacious, with a mean residual AHI of 3.2/h and acceptable leaks. - The mean usage of 5.1 h is also higher than that found in a meta-analysis of 82 clinical trials. |
| Gaisl T, et al. Effects of suboptimal adherence of CPAP therapy on symptoms of obstructive sleep apnoea: a randomised, double-blind, controlled trial. *Eur Respir J*. 2020;55(3):1901526. | Randomized, double-blind, controlled trial | Patients with moderate to severe OSA and pre-treatment EDS (ESS >10) treated with CPAP for ≥12 mo with suboptimal adherence (3–4 h/night) (N=52) | Clinic-based | 3–4 h/night | Baseline:   - Sham: 8.5 (3.9) - CPAP: 9.5 (4.7)   2-wk follow-up:   - Sham: 10.3 (5.6) - CPAP: 8.2 (3.7) - (*P*=0.010) | - CPAP withdrawal for 2 wk resulted in a 2-point increase in ESS score - This suggests that people with moderate to severe OSA who have EDS can substantially benefit from suboptimal CPAP adherence (although less than they might if they had higher adherence). |

^a^Mean (SD) unless otherwise specified.
AHI, Apnea-Hypopnea Index; aOA, adjustable oral appliance; BP, blood pressure; CI, confidence interval; CMT, conservative medical therapy; CPAP, continuous positive airway pressure; CVD, cardiovascular disease; EDS, excessive daytime sleepiness; ESS, Epworth Sleepiness Scale; HRQoL, health-related quality of life; MD, mean difference; MRSE, mask-related side effect; N/A, not applicable; ODI, oxygen desaturation index; OSA, obstructive sleep apnea; OSAHS, obstructive sleep apnea-hypopnea syndrome; OSAS, obstructive sleep apnea syndrome; PAP, positive airway pressure; PSG, polysomnography; QoL, quality of life; RCT, randomized controlled trial; REM, rapid eye movement; SAQLI, Sleep Apnea Quality of Life Index; SAQLI, Sleep Apnea Quality of Life Index; SBP, systolic blood pressure; SD, standard deviation; TST, total sleep time; UAS, upper airway stimulation.

**Supplementary Table 8. Supporting evidence for Search 2C: Within a real-world clinical population,** **what are the potential causes and/or mechanisms of residual EDS in a patient with OSA and how do you sufficiently rule them out?**

| **Supporting evidence for Search 2C: Within a real-world clinical population, what are the potential causes and/or mechanisms of residual EDS in a patient with OSA and how do you sufficiently rule them out?** | | | | | |
| --- | --- | --- | --- | --- | --- |
| **Study** | **Article type/**  **Study design** | **Study population** | **Setting** | **Residual EDS**  **(ESS score)^a^** | **Key finding(s)** |
| Adams GC, et al. An exploration of adult attachment insecurity and psychiatric symptoms in individuals with obstructive sleep apnea. *J Psychosom Res.* 2019;123:109731. | Longitudinal, cross-sectional study | Patients diagnosed with and untreated for OSA (N=102) | Clinic-based | N/A | - Patients with EDS (ESS ≥11) had significantly higher depression scores (median [IQR] PHQ-9, 11.5 [6.0–15.0]) and higher attachment anxiety (mean [SD] ECR anxiety, 3.5 [1.1]) compared with patients without EDS (ESS <11; PHQ-9, 5.5 [3.0–12.0]; ECR anxiety, 3.0 [1.1]; *P*<0.05). |
| Mari P-V, et al. Obstructive sleep apnea in sarcoidosis and impact of CPAP treatment on fatigue. *Sarcoidosis Vasc Diffuse Lung Dis*. 2020;37(2):169-178. | Single-group, open-label, prospective cohort study | Patients with  sarcoidosis (N=68) | Clinic-based | 4.0 (2.8) after 3 mo of CPAP use based on 20 patients | - CPAP reduced fatigue and sleepiness in patients with sarcoidosis. |
| Adams G, et al. Association of daytime sleepiness with obstructive sleep apnoea and comorbidities varies by sleepiness definition in a population cohort of men. *Respirology*. 2016;21(7):1314- 1321. | Telephone survey | Male patients who did not report any previous diagnosis of OSA (N=826) | Population-based | N/A | - ESS score was significantly associated with depression and nocturia, but not with AHI or other PSG indices. |
| Baniak L, et al. The effect of CPAP use on insomnia among persons with type 2 diabetes and obstructive sleep apnea. *Am J Respir Crit Care Med*. 2019;199(suppl):A2648. | Secondary analysis | Patients with comorbid T2DM and OSA (N=71; active CPAP, n=35; sham CPAP, n=36) | N/A | N/A | - CPAP use ≥5 h/night improved insomnia symptoms in patients with OSA and T2DM. |
| Zhang J, et al. White matter structural differences in OSA patients experiencing residual daytime sleepiness with high CPAP use: a non-Gaussian diffusion MRI study. *Sleep Med*. 2019;53:51-59. | Diffusion tensor imaging | Males diagnosed with severe OSA and treated with CPAP (≥6 h/night for ≥30 days) (N=27) | Clinic-based | Non-sleepy group: 6.7 (4.7)  Sleepy group: 8.8 (5.4)  (*P*=0.212) | - In individuals treated with CPAP, those who were sleepy showed higher heterogeneity and diffusion on MRI compared with those who were not sleepy, suggesting global and regional white matter injury in patients with OSA may be responsible for residual EDS (ie, the lack of response to CPAP treatment). |
| El-Sohl AA, et al. Comorbid insomnia and sleep apnea in Veterans with post-traumatic stress disorder. *Sleep Breath*. 2018;22(1):23-31. | Prospective cohort study | Veterans with comorbid insomnia and sleep apnea (COMISA; n=36) or OSA and a history of PTSD (n=36) | Clinic-based | Baseline (COMISA): 12.6 (4.2)  Baseline (OSA): 13.9 (4.6) | - Resolution of EDS (ESS ≤10) occurred in 31.5% of PAP-adherent and 12.8% of PAP-nonadherent patients with COMISA, compared with 52.1% of adherent and 30.4% of nonadherent patients with OSA and PTSD. - Patients with COMISA had greater EDS and worse QoL than patients with OSA and PTSD. - The presence of concomitant insomnia seems to reduce adherence. |
| Kim H, et al. Comorbidity of narcolepsy and obstructive sleep apnea: a case report. *Sleep Med Res*. 2018; 9(2):124-127. | Case report | Case 1: 78-year-old male with ESS=14; diagnosed with narcolepsy with cataplexy and severe OSA  Case 2: 28-year-old with ESS=15; diagnosed with narcolepsy with cataplexy and severe OSA  Case 3: 38-year-old male with ESS=18; diagnosed with narcolepsy without cataplexy and severe OSA | Clinic-based | Patient 1: 14 to 6  Patient 2: 15  Patient 3: 18 to 11 | - Patient 1 started taking modafinil and venlafaxine first, then CPAP was titrated. The patient clinically improved with venlafaxine treatment, as he had no cataplexy events since its initiation. After 2 treatments, ESS score was reduced to 6. - Patient 2 was prescribed 200 mg of modafinil daily but declined CPAP due to financial difficulties. Patient was lost to follow-up. - Patient 3 was prescribed 200 mg of modafinil daily but experienced side effects (headache, myalgia, mood swings, etc.). After modafinil treatment, ESS score improved to 13. After using CPAP, ESS score improved to 11. |
| El-Shabrawy M, et al. Treatment outcome and predictors of better response to thyroxin in hypothyroid patients with sleep apnea syndrome in Zagazig University hospital. *Egypt J Chest Dis Tuberculosis*. 2017; 66(2):285-291. | RCT | Patients with hypothyroid and OSA who started thyroxin therapy (n=65) and patients with euthyroid and OSA who were treated with CPAP (n=65) | Clinic-based | Baseline (hypothyroid): 12.56 (4.53)  Baseline (euthyroid):  10.94 (4.71)  (*P*=0.01)  Post- L-thyroxin: 10.29 (4.23)  Post L-thyroxin+ CPAP: 11.97 (5.29)  (*P*=0.09 vs L-thyroxin) | - Hypothyroid patients with OSA had higher BMI, ESS scores, and neck circumference than controls (patients with euthyroid). - Not all patients with hypothyroidism and OSA will improve on hormonal therapy alone; some may need to continue CPAP. |
| Lang CJ, et al. Associations of undiagnosed obstructive sleep apnea and excessive daytime sleepiness with depression: an Australian population study. *J Clin Sleep Med*. 2017;13(4):575-582. | Telephone interview | Randomly selected, community dwelling males without prior diagnosis of OSA (N=837) | At home and sleep lab | N/A | - An interaction between OSA and EDS was observed that is additive in terms of increasing the associations seen between OSA, EDS, and depression. The interaction was mostly clear in the group with mild–moderate OSA, resulting in a significant association with depression only when men had both mild–moderate OSA and EDS. |
| Neu D, et al. Complex sleep apnea at auto-titrating CPAP initiation: prevalence, significance and predictive factors. *Clin Respir J.* 2017; 11(2):200-209. | Retrospective cohort study | Patients with complex sleep apnea (CompSA; N=24) and controls (N=239) | Clinic-based | N/A | - AHI of patients with CompSA decreased from 52.7 events/h to 39.9 events/h compared with 40.9 to 7.3 events/h in patients without CompSA, suggesting a lack of adequate treatment response to CPAP in patients with CompSA. |
| Xiong Y, et al. Brain white matter changes in CPAP-treated obstructive sleep apnea patients with residual sleepiness. *J Magn Reson Imaging*. 2017; 45(5):1371-1378. | Diffusion tensor imaging study | Males with severe OSA who were treated with CPAP (≥6 h/night for ≥30 days) (N=29) | Clinic-based | After CPAP treatment (sleepy group): 8.9 (5.0)  After CPAP treatment (non-sleepy group):  5.7 (4.5) | - Patients with residual EDS due to OSA showed significantly different mean diffusivity (MD) and radial diffusivity in the whole brain white matter (WM) analysis and altered fractional anisotropy (FA) and MD values in specific WM regions compared with non-sleepy patients with similar high levels of CPAP use. - FA and MD can serve as sensitive imaging markers for the differing responses to CPAP treatment on OSA patients. |
| Chapman JL, et al. Residual daytime sleepiness in obstructive sleep apnea after continuous positive airway pressure optimization: causes and management. *Sleep Med Clin*. 2016;11(3):353-363. | Review | N/A | N/A | N/A | - Concomitant sleep disorders, mood disorders that coexist with or occur years after OSA diagnosis, medications (antihistamines, anticonvulsants, analgesics, certain antidepressants), neurological conditions (Parkinson’s, myotonic dystrophy, multiple sclerosis), and poor sleep hygiene can all be causes of residual EDS. Practitioners should evaluate for the presence of uncontrolled diabetes, hypothyroidism, anemia, and obesity, which can all contribute to feelings of tiredness and sleepiness. |
| Bjorvatn B, et al. Prevalence and correlates of insomnia and excessive sleepiness in adults with obstructive sleep apnea symptoms. *Percept Mot Skills*. 2014;118(2):571-586. | Survey | National Population Registry in Norway (N=1502) | Population-based | OSA symptoms were found in 6.2% of participants. EDS/ hypersomnia (ESS >10) was found in ~30% of these participants | - Hypersomnia was found to be more prevalent in patients with OSA symptoms than those without. |
| Bjornsdottir E, et al. Symptoms of insomnia among patients with obstructive sleep apnea before and after two years of positive airway pressure treatment. *Sleep*. 2013;36(12):1901-1909. | Longitudinal cohort study | Adults with OSA assessed prior to and 2 y after starting PAP treatment; 3 subtypes of insomnia were defined: initial (difficulty initiating sleep); middle (difficulty maintaining sleep); and late (early morning awakenings) (N=705) | Clinic-based | Baseline: 11.9 (5.0)  ESS change after 2 y (persistent vs improved insomnia):  Initial: −1.0 (4.3) vs −3.5 (4.8); (*P*=0.006)  Middle: −2.6 (4.5) vs −4.6 (5.0); (*P*<0.001)  Late: −1.8 (4.8) vs −3.9 (4.5); (*P*=0.002) | - Insomnia symptoms are common among patients with OSA, especially symptoms of middle insomnia, and these types of symptoms generally improve with PAP treatment. - Symptoms of initial insomnia tend to persist even though patients adhere to PAP and can also negatively affect adherence to PAP treatment. - Late insomnia symptoms are likely to improve in patients with OSA who are PAP nonusers. |
| Boethel CD, et al. Residual sleepiness in obstructive sleep apnea: differential diagnosis, evaluation, and possible causes. *Sleep Med Clin*. 2013;8(4):571-582. | Review | N/A | N/A | N/A | - Chronic intermittent hypoxia could result in neuronal brain injury through oxidative stress and inflammation/apoptosis. - Behaviorally induced insufficient sleep may lead to EDS in patients with OSA despite adequate nightly CPAP use. - Depression is prevalent among patients with OSA; the complex relationship between these conditions makes it difficult to assess underlying cause and impact of treatment. - RLS could cause insomnia, reducing total sleep time, resulting in EDS the following day. - Studies show the prevalence of OSA in narcolepsy patients ranges from 2% to 68%; this could result in residual EDS. - Inadequate CPAP treatment could also be a cause of residual EDS. - Cytokines, chemokines, and hormonal changes may cause neuronal injury leading to residual daytime sleepiness. |
| Launois SH, et al. On treatment but still sleepy: cause and management of residual sleepiness in obstructive sleep apnea. *Curr Opin Pulm Med*. 2013;19(6): 601-608. | Review | N/A | N/A | N/A | - Experimental studies in mice show intermittent hypoxia, possibly via cell injury and apoptosis after activation of inflammatory and oxidative pathways, causes permanent brain damage in regions implicated in wake and sleep regulation; these lesions were associated with sleepiness when the mice were returned to a normoxic environment. - Sleep disturbances or sleep deprivation could account for the inconsistent effect of CPAP. - Stimulant drugs may be needed in patients with residual sleepiness, especially those who drive. |
| Otuyama LJ, et al. The cholinergic system may play a role in the pathophysiology of residual excessive sleepiness in patients with obstructive sleep apnea. *Med Hypotheses*. 2013;81(3):509-511. | Hypothesis | N/A | N/A | N/A | - Acetylcholine (ACh) degradation by acetylcholinesterase in the brainstem was lower in patients with Alzheimer’s disease with sleepiness than in patients without sleep disturbances. - Decreased central cholinergic activity may play a role in the pathophysiology of residual EDS in patients with CPAP-treated OSA. - Central cholinergic medications may be a more specific treatment for this neurological condition. |
| Castiglioni P, et al. Why excessive sleepiness may persist in OSA patients receiving adequate CPAP treatment. *Eur Respir J*. 2012;39(1):226-227. | Hypothesis (Letter to the Editor) | N/A | N/A | N/A | - Obesity hypoventilation syndrome or a comorbidity with COPD, the so-called overlap syndrome, could be present in the OSA group with residual EDS. - Residual EDS could be linked to the possible presence of cardiovascular autonomic dysregulations induced by autonomic or “subcortical” arousals. - Testing for hypercapnia or autonomic alterations during sleep could improve understanding of the mechanisms responsible for RES in CPAP-treated OSA patients. |
| Stepnowsky CJ, et al. Fatigue in sleep apnea: the role of depressive symptoms and self-reported sleep quality. *Sleep Med*. 2011;12(9):832-837. | Interventional study | Patients with OSA who were CPAP-naïve (men, n=232; women n=8); depressive subgroups based on baseline CESD-10 score of <8 (low; n=70) or ≥8 (high; n=164) | At home | Baseline  All patients: 12.4 (5.6)  CESD-10 <8: 10.0 (5.4)  CESD-10 ≥8 : 13.4 (5.4)  (*P*<0.001 low vs high CESD) | - Depressive symptoms play a significantly larger role in fatigue than OSA severity. - The same combination of variables, including demographics, comorbidities, OSA severity, depressive symptoms, and self-reported sleep quality can explain a larger percentage of the variance in daytime fatigue than EDS (as measured by ESS). - Results support a greater association between fatigue and depressive symptoms than between fatigue and AHI or oximetry variables. |
| Habukawa M, et al. Effect of CPAP treatment on residual depressive symptoms in patients with major depression and coexisting sleep apnea: contribution of daytime sleepiness to residual depressive symptoms. *Sleep Med*. 2010;11(6):552-557. | Interventional study | Patients with suspected OSA who had been treated for MDD with antidepressants  and/or benzodiazepines (N=17) | Clinic-based | 2 mo of CPAP resulted  in a significant improvement in ESS scores (decreased from 13.2 [2.5] to 8.1 [3.5]; *P*<0.01) | - CPAP resulted in significant improvement in residual depressive symptoms. There was a significant correlation between improvement rates in residual depressive symptoms, as measured by BDI and HAMD, and those in self-reported daytime sleepiness by ESS (R=0.86, *P*<0.001 and R=0.75, *P*<0.01, respectively). |
| Fong SY, et al. Excessive daytime sleepiness in obstructive sleep apnea patients after home CPAP treatment: a long-term outcome study. *Sleep Biol Rhythms*. 2009;7(3):193-200. | Retrospective chart review study | Treatment-naïve patients with OSA (N=131) | Clinic-based | Pre-CPAP: 12.80 (5.29)  Post-CPAP (mean 3.4 [2.4] y treatment): 9.95 (4.95); *P*<0.001  38.4% (20/52) had residual ESS ≥14 despite regular CPAP treatment | - Persistence of EDS (assessed by ESS) despite adequate PAP use was not related to OSAS severity, obesity, or the result of inadequate CPAP pressure. - Hypoxic-related neuronal damages due to untreated OSAS has been proposed to cause residual EDS, but a proportional relationship between EDS and duration of untreated OSAS was not shown in the results. - Findings suggested the likelihood of genetic contribution (eg, HLA-DQB1*0602) in the shortened MSL and presence of post-CPAP sleep-onset REM periods in the subjects. |
| Koutsourelakis I, et al. Predictors of residual sleepiness in adequately treated obstructive sleep apnoea patients. *Eur Respir J*. 2009;34(3): 687-693. | Interventional study | Patients with OSA and EDS (ESS >10) (N=208) | Clinic-based | CPAP responders (n=94): 14.0 (3.0)  CPAP non-responders (n=114, follow-up 6 mo): 16.2 (3.0) | - Independent predictors of residual EDS following CPAP therapy were diabetes, heart disease, and a higher ESS and lower RDI score at baseline assessment. |
| Krystal AD, et al. Randomized, double-blind, placebo-controlled study of armodafinil in patients with residual excessive sleepiness associated with treated obstructive sleep apnea and comorbid depressive disorders. *Chest*. 2009;136(4 suppl): 70S. | Multicenter, randomized, double-blind, placebo-controlled, parallel-group study | CPAP-treated patients with OSA and RES, as well as an MDD or dysthymic disorder requiring antidepressant monotherapy | Clinic | N/A | - Patients with RES associated with CPAP-treated OSA who have a comorbid depressive disorder reported fatigue and functional impairment. |
| Lal C, et al. Excessive daytime sleepiness in obstructive sleep apnea: mechanisms and clinical management. *Ann Am Thorac Soc*. 2021;18(5): 757-768. | Review | N/A | N/A | N/A | - Mechanisms underlying residual EDS in patients with treated OSA are likely confounded by the unknown length of time a person has been exposed to the condition and differences in individual susceptibility to the consequences of OSA, as well as comorbid conditions, such as mood disorders and neurologic conditions. - Intermittent hypoxia is associated with oxidative injury, which in turn, is associated with wake impairments. - Chronic sleep fragmentation can result in wake impairments associated with neuronal degeneration and oxidative injury. - Neuroimaging studies show evidence of neuronal injury, however, the pathophysiologic mechanisms underlying residual EDS in OSA remain unclear. - Current treatments for EDS in OSA demonstrate improvements in measures of EDS, QoL, and work productivity. |
| Macey PM, et al. Relationship between obstructive sleep apnea severity and sleep, depression and anxiety symptoms in newly-diagnosed patients. *PLoS One*. 2010;5(4):e10211. | Cross-sectional study | Newly diagnosed and untreated patients with OSA without major comorbidities (N=49) | Clinic-based | N/A | - OSA was associated with abnormally high levels of depressive and anxious symptoms, daytime sleepiness, and poor sleep quality. - AHI is not necessarily a reliable predictor of OSA severity. |
| Barbera C, et al. Hot topic: residual daytime sleepiness (RES) after obstructive sleep apnea (OSA) treatment in the European Sleep Apnea Database (ESADA) study: prevalence and predictors. *J Sleep Res*. 2020;29(suppl 1):81. [Abstract] | Prevalence study | Patients with OSA treated with CPAP (N=5515) | Population-based | Pre- vs post-treatment  Total sample: Mean ESS 10.4 (5.2) vs 6.7 (4.8); prevalence of EDS 56.9% vs 28.3% Subjects with residual AHI data (n=2349): Mean ESS 10.2 (5.1) vs 8.0 (5.0); prevalence of EDS 54.0% vs 35.5% | - Stratification according to CPAP use and residual AHI revealed no significant differences in prevalence of RES or other variables among groups. - No comorbidity (ie, coronary artery disease, diabetes, hypertension, psychiatric disorders, COPD, or insomnia) significantly predicted RES. - RES occurred in >25% of CPAP-treated OSA patients. - RES is a complex clinical challenge involving other, yet unidentified, factors. |
| Passini VV, et al. Narcolepsy type 1 and REM sleep without atonia (RSWA): what is the relationship to REM sleep behavior disorder (RBD) and obstructive sleep apnea? Case report. *Brazilian Sleep Congress Abstract*. 2019. [Abstract] | Case report | Patient with a history of EDS since age 15 and diagnosed with narcolepsy at age 32 (N=1) | N/A | N/A | - Given the difficulty in controlling narcolepsy symptoms, investigating other sleep disorders, such as REM sleep behavior disorder and OSA, is important. Inquiring about dream enactment or symptoms of OSA may help define the differential diagnosis. |
| Tamisier R, et al. Prevalence and determinants of residual excessive daytime sleepiness (RES) in patients treated with continuous positive airway pressure (CPAP) for obstructive sleep apnea (OSA). *Eur Respir J*. 2020; 56(suppl 64):2493. [Abstract] | Cohort study | Patients with severe OSA treated with CPAP for >3 mo (N=307) | Database | 21 patients (5.54%) had RES (ESS >10) with no obvious cause of sleepiness,  adequate CPAP daily use (>4 h), and no severe cardiovascular comorbidity | - 5.54% of patients with OSA had RES and a possibly altered QoL and vigilance, despite being well treated. |
| De Lumban TC, et al. Sleep disturbances in thyroid malignancies. *Am J Respir Crit Care Med*. 2019;199 (suppl 1): A2282. [Abstract] | Retrospective review | Patients with thyroid malignancies who underwent a sleep consultation (N=54) | N/A | N/A | - There is a high prevalence of sleep disorders in patients with thyroid malignancies; screening these patients for OSA is recommended. |
| Velamuri K, et al. REM-only sleep apnea: an underrecognized disorder. *Sleep*. 2019;42(suppl): A188. [Abstract] | Retrospective review | Adults with REM-only OSA (N=420) | Clinic-based | N/A | - Patients with REM-only OSA have a similar degree of EDS and similar incidence of cardiovascular and metabolic comorbidities. Diagnosing these patients with OSA and starting treatment could be useful in reducing their EDS as well as comorbidities. |
| Velazquez VG, et al. Sleep Apnoea Syndrome (SAS) in dysthymia treated with selective serotonin reuptake inhibitors (SSRIs): profile of patients with residual excessive daytime sleepiness (EDS). *Eur Respir J*. 2019;54 (suppl 63):PA857. [Abstract] | 3-y prospective observational study | Patients with dysthymia treated with SSRIs and referred to sleep lab to rule out OSA due to EDS (ESS ≥12) and other related symptoms (N=66) | Clinic-based | Baseline: 14 (2.6)  RES with CPAP:  12.3 (1.4) | - EDS is the most common symptom before diagnosis of OSA in patients who have SSRI-treated dysthymia and residual EDS. - After sufficient CPAP treatment, patients who have dysthymia exhibit a sleep profile with a significantly greater number of sleep cycles, spontaneous arousals, and % REM stage and lower % of deep sleep; they also require lower CPAP pressures to manage SAS. |
| Wohlgemuth W, et al. Sleep apnea phenotypes in US veterans. *Psychosom Med*. 2019;81(4):A11-A12.  [Abstract] | Prospective study | Veterans diagnosed with OSA (N=660) | Clinic-based | N/A | - Insomnia and EDS in patients with OSA may be attributed to comorbid psychiatric conditions (eg, mood disorder, PTSD, and chronic pain). |
| Budhiraja R, et al. Prevalence and predictors of subjective  and objective sleepiness in patients with obstructive sleep apnea (OSA). *Sleep*. 2017;40(suppl 1):A169-A170. [Abstract] | Cohort study | Patients diagnosed with OSA (N=1105) | N/A | N/A | - ESS scores correlated with BMI, AHI, and HAMD score, and inversely correlated with age. - Patients with depression and chronic pain had higher ESS scores (>10). - Linear regression model revealed that younger age, greater AHI, and higher HAMD scores are independently associated with higher ESS scores. |
| Luyster FS, et al. Psychosocial and sleep characteristics in comorbid insomnia and sleep apnea. *Sleep*. 2015;38(suppl): A155-A156. [Abstract] | Prospective study | Adults with insomnia (n=17), OSA (n=50), or COMISA (n=10) | Clinic-based | N/A | - - Patients with COMISA appear to have psychosocial and sleep characteristics similar to those with insomnia only and OSA only, with the exception of self-reported sleep disturbance, which was similar to those with insomnia only and tended to be worse than those with OSA only. |
| Wohlgemuth W, et al. Latent profiles of depression in sleep apnea. *Psychosom Med*. 2015; 77(3):A9-A10. [Abstract] | Interventional study | Patients recently diagnosed with OSA (N=85) | Clinic-based | N/A | - Both self-reported insomnia and objectively measured sleep-related breathing dysfunction were related to severe depression in patients with OSA. |
| Sutton H. Persistent somnolence despite adequate CPAP therapy. *J Sleep Res*. 2014; 23(suppl 1):300. [Abstract] | Audit | Patients with CPAP-treated OSA with residual EDS (N=64) | Clinic-based | Not reported | - The greatest cause of EDS in these patients was attributable to periodic limb movement disorders. |
| Wohlgemuth W, et al. Comorbid insomnia and sleep apnea: complex relationships with daytime fatigue and sleepiness. *Sleep*. 2014;37 (suppl):A162. [Abstract] | Interventional study | Veterans with COMISA (prior to initiating CPAP) (N=440) | Clinic-based | N/A | - Insomnia symptoms at night were positively correlated with fatigue and sleepiness during the day. - OSA was not correlated with EDS and was inversely associated with daytime fatigue. |
| Wohlgemuth W, et al. CPAP adherence as a mediator between co-morbid insomnia, OSA and subjective daytime sleepiness. *Sleep Med*. 2013; 14(suppl 1):e306.[Abstract] | Observational | Veterans with OSA at CPAP follow-up (N=237) | Clinic-based | Not reported | - CPAP adherence was not a significant mediator between nighttime symptoms and daytime sleepiness. Only nighttime insomnia symptoms were related to daytime sleepiness. - Insomnia was negatively associated with adherence and positively associated with self-reported EDS. - Neither AHI nor adherence were associated with EDS. |
| Weaver TE. Failure to return to normal following optimal CPAP use. *J Sleep Res.* 2011; 20(suppl 1):9. [Abstract] | Symposia presentation | N/A | N/A | N/A | - Proposed causes of residual EDS include suboptimal CPAP, insufficient sleep time, somnolence-associated medications, comorbid conditions, or a second sleep disorder. - There is emerging evidence in animal models of injury to the wake-promoting ventral periaqueductal gray dopaminergic neurons in conditions of long-term intermittent hypoxemia, similar to that associated with OSA, which could explain residual EDS. |
| Vernet C, et al. Residual sleepiness in patients with obstructive sleep apnea/ hypopnea syndrome: a central post-hypoxic hypersomnia? *Sleep*. 2010;33(suppl): A115. [Abstract] | Comparative study | Patients with  OSA and adequate CPAP with (n=20) and without (n=20) residual EDS and healthy controls (n=20) | Clinic-based | 16.4 (3) | - The phenotype of RES in patients with OSA includes sleepiness, memory, mood, attention, and fatigue complaints that may go unnoticed by objective tests. - The association of RES, periodic leg movements, and decreased mood without depression may be caused by post-hypoxic lesions in noradrenalin, dopamine, and serotonin systems in selectively vulnerable patients. |
| Black J. Sleepiness and residual sleepiness in adults with obstructive sleep apnea. *Respir Physiol Neurobiol*. 2003; 136(2-3):211-220. [Abstract] | Review | N/A | N/A | N/A | - It has been hypothesized that microarousal disturbs the restorative processes of sleep and has been found to produce sleepiness and/or daytime functioning deficits when stimulated by sensory stimuli in normal participants. - Reductions in slow-wave sleep (stages 3 and 4) and REM percentages, with subsequent increases in lighter sleep, characterize these changes. - An increase in respiratory effort among patients with OSA may lead to EDS. - Other possible factors include modifications in body metabolism, hormonal activity, and endogenous somnogenic substance production (eg, cytokines). - Residual EDS could be caused by CNS malfunction not related to acute OSA, but rather a longer term effect of OSA. - Other causes could include comorbid sleep disorders, low total sleep time, suboptimal sleeping environment, or comorbid psychiatric conditions. - In a majority of people with residual EDS, CPAP re-titration may be necessary. |
| Jagadish S, et al. Autonomic dysfunction in childhood hypersomnia disorders. *Sleep Med*. 2021;78:43-48. | Retrospective chart review | Patients (<18 y) with hypersomnia (N=89) | Clinic-based | N/A | - Orthostatic intolerance is an important comorbidity in primary hypersomnia disorders of childhood, with female predominance. - Asking about orthostatic intolerance symptoms during initial presentation in patients with EDS and utilizing autonomic laboratory services for systematic investigation and management should be standard practice in sleep medicine. |
| Monderer R, et al. Evaluation of the sleepy patient: differential diagnosis. *Sleep Med Clin.* 2017;12(3):301-312. | Systematic review | N/A | N/A | N/A | - Evaluating a patient presenting with EDS begins with a thorough clinical assessment including detailed sleep history, medical, psychiatric, medications, and social and family history. - Physical exam should include a general medical exam, neurologic exam, and an exam of the upper airway. - A sleep log can be very helpful. - The history and physical exam will often uncover the suspected cause of EDS. - Objective testing (PSG and possibly MSLT) will help confirm diagnosis and lead to an appropriate treatment plan. |
| Ejaz SM, et al. Obstructive sleep apnea and depression: a review. *Innov Clin Neurosci*. 2011;8(8):17-25. | Review/ literature search | N/A | N/A | N/A | - Per this literature search, the prevalence of depression in those with OSA is 5% to 63%. - An exact pathophysiological relationship between OSA and depression is not fully understood. |

^a^Mean (SD) unless otherwise specified.
ACh, acetylcholine; AHI, Apnea-Hypopnoea Index; BDI, Beck Depression Inventory; BMI, body mass index; CESD-10, Center for Epidemiologic Studies Short Depression Scale; CNS, central nervous system; COMISA, comorbid insomnia and obstructive sleep apnea; CompSA, complex sleep apnea; COPD, chronic obstructive pulmonary disease; CPAP, continuous positive airway pressure; CTRW, continuous-time random-walk; ECR, Experiences in Close Relationships; EDS, excessive daytime sleepiness; ESS, Epworth Sleepiness Scale; FA, fractional anisotropy; HRSD, Hamilton Rating Scale for Depression; IQR, interquartile range; ISI, Insomnia Severity Index; MD, mean diffusivity; MDD, major depressive disorder; MSLT, multiple sleep latency test; MWT, maintenance of wakefulness test; OSA, obstructive sleep apnea; OSAS, obstructive sleep apnea syndrome; PAP, positive airway pressure; PHQ-9, Public Health Questionnaire-9; PSG, polysomnography; PSQI, Pittsburgh Sleep Quality Index; PTSD, post-traumatic stress disorder; QoL, quality of life; RBD, REM behavior disorder; RDI, respiratory distress index; REM, rapid eye movement; RES, residual excessive daytime sleepiness; RLS; restless leg syndrome; SAS, sleep apnea syndrome; SD, standard deviation; SSRIs, selective serotonin reuptake inhibitors; T2DM, type 2 diabetes mellitus; WM, white matter.

**Supplementary Table 9. Supporting evidence for Search 3A: How do you approach patients with EDS who are intolerant to PAP therapy or have attempted/are attempting therapy?**

| **Supporting evidence for Search 3A: How do you approach patients with EDS who are intolerant to PAP therapy or have attempted/are attempting therapy?** | | | | |
| --- | --- | --- | --- | --- |
| **Study** | **Article type/**  **Study design** | **Study population** | **Setting** | **Key finding(s)** |
| Brostrӧm A, et al. Development and psychometric evaluation of the Motivation to Use CPAP Scale (MUC-S) using factorial structure and Rasch analysis among patients with obstructive sleep apnea before CPAP treatment is initiated. *Sleep Breath.* 2021;25:627-637. | Psychometric validation study with cross-sectional design | Patients diagnosed with and untreated for OSA (N=193) | Clinic-based | - The MUC-S is a tool that explores the impetus related to CPAP treatment as well as evaluates CPAP treatment effects. - Patients with high motivation had higher levels of EDS, more problematic insomnia symptoms, poorer global perceived health, and a more positive attitude toward CPAP treatment. |
| Giannasi L, et al. Patients who refused CPAP have a safe option to treat severe obstructive sleep apnea: pilot study. *Sleep* *Science*. 2020. [Abstract] | Prospective study | Patients with severe OSA who refused CPAP therapy (N=17) | Clinic-based | - OSA severity, ODI, REM sleep, SaO_2_ nadir, and ESS improved after 6 mo of OAm therapy. - OAm is a safe alternative to treating severe OSA in patients who refuse, or are intolerant to, CPAP therapy. |
| Dauvilliers Y, et al. Pitolisant for daytime sleepiness in patients with obstructive sleep apnea who refuse continuous positive airway pressure treatment: a randomized trial. *Am J* *Respir Crit Care Med.* 2020;201:1135-1145. | 12-wk, phase 3, prospective, double-blind, placebo-controlled, randomized, parallel-group trial | Patients with moderate to severe OSA (AHI ≥15 events/h) and EDS (ESS ≥12) who refused CPAP treatment (pitolisant, n=201; placebo, n=67) (N=268) | Clinic-based | - Pitolisant improved EDS; mean (SD) change in ESS score from baseline to end of treatment: pitolisant, −6.3 (4.5); placebo, −3.6 (5.5); (*P*<0.001). - There was no significant difference in the ratios of increase in mean sleep latency during OSLER tests (*P*=0.108). - A significantly higher number of patients reported improvements in CGI-S on pitolisant (84.2%) vs placebo (56.3%); (*P*<0.001). - A significantly higher number of patients reported improvements in PGO on pitolisant (86.3%) vs placebo (60.9%); (*P*<0.001). - Pitolisant was effective at reducing fatigue; mean (SD) reduction in Pichot fatigue scale score: pitolisant, −3.6 (5.6); placebo, −1.0 (6.3); (*P*=0.005). |
| Kennedy B, et al. Pressure modification or humidification for improving usage of continuous positive airway pressure machines in adults with obstructive sleep apnoea. *Cochrane Database Syst Rev*. 2019;12:CD003531. | Review of parallel group or crossover RCTs (blinded and unblinded) | 64 studies recruiting 3922 patients with a history of OSA | N/A | - Auto-CPAP improved average nightly use modestly vs fixed CPAP: MD (95% CI) = 0.21 h/night (0.11–0.31). - Reductions in ESS score between auto-CPAP and fixed CPAP were small: MD (95% CI) = −0.44 (−0.72 to −0.16). - On average, patients preferred auto-CPAP in 8/14 studies but fixed CPAP in 6/14 studies. - Humidification plus fixed CPAP may increase use vs fixed CPAP alone: MD (95% CI) = 0.37 h/night (0.10–0.64). |
| Spurr KF. Adherence to positive airway pressure therapy. *Can J Respir Ther*. 2018;54:28. [Abstract] | Conference abstract | N/A | N/A | - The gold-standard therapy for the management of OSA in adults is noninvasive positive pressure therapy administered via CPAP. - Patients experiencing underlying lung disease or those who are unable to use CPAP resort to BiPAP. - PAP adherence is vital in patients with concomitant acute or chronic disease. |
| Siv J, et al. Novel predictors of CPAP adherence among patients with acute stroke. *Am J Respir Crit Care Med*. 2018;197:A7451. [Abstract] | SCOUTS2, single-arm trial | Patients undergoing inpatient rehabilitation after stroke (N=60) | Clinic-based | - Patients adherent to CPAP treatment had longer inpatient CPAP exposure: adherent, 8 days (IQR, 6–17); nonadherent, 5 days (IQR, 3–8); (*P*=0.05). - Daytime sleepiness was not associated with 3-mo adherence. - Inpatient OSA treatment may improve CPAP adherence following recovery from stroke. |
| Tippin J, et al. Sleep remains disturbed in obstructive sleep apnea patients treated with positive airway pressure: a three-month cohort study using continuous actigraphy. *Sleep Med*. 2016;24:24-31. | Actigraphy study | Patients meeting ICSD-2 criteria for OSA with no previous PAP treatment (N=80) and controls (N=50) | Clinic-based | - 3 mo of PAP use reduced the percentage of OSA patients with EDS from 60% to 17%. - Prevalence of EDS in comparison participants remained at 14%. - AHI, RDI, and SpO_2_ nadir were significant predictors of patients’ initial response to PAP therapy. - Self-reported symptoms as reported in ESS and FOSQ improve slowly after introducing PAP. |
| Epstein M, et al. CPAP machine inaccuracy in detecting residual obstructive sleep apnea. *Sleep*. 2016. [Abstract] | 12-mo, single-arm study | Patients with potential OSA (N=92) | At home | - WatchPat AHI was significantly higher in some patients with suspected residual OSA (ie, residual, untreated respiratory disturbances). - CPAP machines may be unreliable in detecting residual OSA. - While CPAP machines registered normal mean (range) AHI of 2.1 (0–5.5) for all 92 patients, WatchPAT registered 31 patients with elevated mean (range) AHI of 10.8 (0–27). |
| Azbay S, et al. The influence of multilevel upper airway surgery on CPAP tolerance in non-responders to obstructive sleep apnea surgery. *Eur Arch Otorhinolaryngol*. 2016; 273:2813-2818. | Retrospective cohort study | Patients who underwent surgery due to CPAP intolerance, had residual OSA after surgery, and still required CPAP therapy (N=67) | Clinic-based | - Patients previously intolerant of CPAP had high rates of CPAP acceptance (100%), prescription (58.2%), adherence (50.7%), and tolerance (47.8%) after OSA surgery. - Most patients (82.1%) reported no significant adverse events during postoperative CPAP use. - Patients who were intolerant of CPAP but did not respond to OSA surgery were tolerant of CPAP after surgery. |
| Wohlgemuth WK, et al. Attempters, adherers, and non-adherers: latent profile analysis of CPAP use with correlates. *Sleep Med*. 2015;16:336-342. | Retrospective correlational study | Veterans with OSA (N=207) | Clinic-based | - Greater self-efficacy, less insomnia, higher baseline AHI, longer time since CPAP initiated, and higher CPAP pressure predicted adherence. - Insomnia symptoms were associated with nonadherence to CPAP therapy: OR=1.149; (*P*=0.004). - Patients with lower reported self-efficacy scores were less likely to be adherent to CPAP therapy (OR=0.109; *P*=0.005). |
| Carlucci A, et al. Efficacy of bilevel-auto treatment in patients with obstructive sleep apnea not responsive to or intolerant of continuous positive airway pressure ventilation. *J Clin Sleep Med*. 2015;11:981-985. | Prospective study | Patients with OSA who were not responsive to CPAP (n=35) or intolerant of CPAP (n=31) | Clinic-based | - In patients not responsive to CPAP, switching to bilevel-auto CPAP significantly reduced mean (SD) AHI vs CPAP: 30.4 (16.1) vs 4.6 (4.9) (*P*<0.0001). - In patients intolerant of CPAP, switching to bilevel-auto CPAP significantly reduced mean (SD) AHI vs CPAP: 14.9 (9.1) vs 4.2 (3.3) (*P*<0.0001). - Bilevel-auto CPAP may be an alternative for patients who are not responsive to, or intolerant of, CPAP. |
| Patel J, Ruoff C. Oral pressure therapy: an effective alternative for treatment of obstructive sleep apnea after failing both CPAP and surgical approach. *Sleep*. 2015. [Abstract] | Case study | 54-year-old man with history of OSA who was intolerant of CPAP and underwent surgery but still had OSA | At-home | - Oral pressure therapy may treat OSA in patients who have failed CPAP and surgical approaches. |
| Ng R. CPAP compliance: effects of CPAP adaptation periods, OSA severity and other parameters on CPAP compliance. *Sleep*. 2014. [Abstract] | Retrospective chart review; survey | Patients diagnosed with moderate to severe OSA (N=97) | N/A | - Patients with more severe OSA were more CPAP adherent compared with moderate OSA patients (*P*<0.02). - CPAP trial of 7–14 days was associated with better adherence compared with shorter and longer CPAP adaptation periods (*P*<0.04). |
| Mori H, et al. Factors associated with CPAP acceptance in the patients with sleep apnea and cardiovascular disease. *Eur Heart J.* 2014. [Abstract] | Prospective study | Japanese patients with CVD who met clinical criteria for CPAP therapy (N=168) | Clinic-based | - 70 patients (41.7%) accepted CPAP. - CPAP acceptance was associated with higher EDS, lower QoL, higher self-reported sleep disturbance, and higher obstructive apnea index. - The absence of self-reported sleepiness in patients with CVD may reduce CPAP acceptance. |
| Diaferia G, et al. Effect of speech therapy as adjunct treatment to continuous positive airway pressure on the quality of life of patients with obstructive sleep apnea. *Sleep Med*. 2013. [Abstract] | RCT | Men with OSA [sham speech therapy (n=24), speech therapy (n=27), CPAP with no speech therapy (n=27), or CPAP + speech therapy (n=22)] | N/A | - Patients in the speech therapy and CPAP with speech therapy groups had improved physical domain scores on the WHOQoL-Brief and in the functional capacity domain of the SF-36. - Speech therapy, with or without CPAP, may be an alternative treatment for QoL improvement in patients with OSA. |
| Dettenmeier P, et al. Evaluation of a continuous positive airway pressure desensitization protocol for CPAP-intolerant patients: a pilot study. *Chest*. 2013;144:979A. [Abstract] | Retrospective cohort study | Patients with OSA who were CPAP-intolerant (N=22) | N/A | - CPAP-adherent group had higher CPAP use at 30 days (339.5 vs 47.22 min; *P*<0.001) and 90 days (361.75 vs 4.33 min; *P* <0.001). - CPAP desensitization modestly improves CPAP adherence. |
| Yang MC, et al. Factors affecting CPAP acceptance in elderly patients with obstructive sleep apnea in Taiwan. *Respir Care*. 2013;58:1504-1513. | Retrospective cohort study | Patients with OSA; categorized as young (n=35), middle age (n=169), or elderly (n=111) | Clinic-based | - BiPAP use was significantly higher in elderly patients than in middle-aged patients (17.1% vs 1.4%; *P*=0.02). - Smoking was associated with lower CPAP adherence: OR (95% CI) = 0.33 (0.13–0.82); (*P*=0.02). |
| Björnsdottir E, et al. Symptoms of insomnia among patients with obstructive sleep apnea before and after two years of positive airway pressure treatment. *Sleep*. 2013;36:1901-1909. | Prospective study | Patients recently diagnosed with OSA who were about to start PAP treatment (N=705) | Clinic-based | - 2-y PAP therapy significantly reduced the prevalence of symptoms of middle insomnia: PAP users, 30.7%; PAP nonusers, 43.5%; (*P*=0.001). - Patients with initial insomnia at baseline were more likely to be PAP nonusers: OR (95% CI): 0.59 (0.38–0.91); (*P*=0.01). - Patients with late-night insomnia at baseline were more likely to be PAP nonusers: OR (95% CI) = 0.55 (0.39–0.79); (*P*<0.001). - Symptoms of insomnia are common in patients with OSA and generally improved with PAP treatment. |
| Basoglu O, et al. Adherence to continuous positive airway pressure therapy in obstructive sleep apnea syndrome: effect of visual education. *Sleep Breath*. 2012;16:1193-1200. | 6-mo prospective study | Patients with newly diagnosed moderate to severe OSA receiving visual education (n=66) or no visual education (control; n=67) | Clinic-based | - Rates of CPAP adherence after 6 mo were numerically higher in the visual education group vs control group: 71.2% vs 56.7%; (*P*=0.08). - Patients who were adherent saw improvements in snoring, apnea, daytime sleepiness, unrefreshed sleep, nocturnal choking, nocturia, morning headache, night sweating, insomnia, dry mouth, reflux, and nocturnal cough. - Adherence to CPAP may be improved by visual education, and patients who were adherent showed improvements in many symptoms of OSA. |
| Bakker JP, Marshall NS. Flexible pressure delivery modification of continuous positive airway pressure for obstructive sleep apnea does not improve compliance with therapy: systematic review and meta-analysis. *Chest*. 2011;139:1322-1330. | Systematic review and meta-analysis | 10 RCTs comparing flexible-pressure CPAP (C-Flex) to fixed pressure CPAP in 599 patients | N/A | - Adherence with C-Flex was similar to adherence with fixed-pressure CPAP: MD (95% CI) = 0.16 h (−0.09 to 0.42) favoring C-Flex; (*P*=0.21). - Treatment efficacy was similar, nonsignificantly favoring C-Flex: MD (95% CI) = −0.31 events/h (−1.33 to 0.72); (*P*=0.56). - Improvements in ESS scores were also similar: MD (95% CI) = 0.76 (−3.82 to 2.30) out of 24 points; (*P*=0.63). - C-Flex does not provide additional adherence, efficacy, or sleepiness benefit over standard CPAP. |
| Walsh J, et al. A convenient expiratory positive airway pressure device is effective for the treatment of sleep apnoea in many patients non-adherent with positive airway pressure. *J Sleep Res*. 2010. [Abstract] | Prospective study | Patients who refused or were nonadherent to PAP therapy and tolerated EPAP device (N=47) | Clinic-based | - EPAP therapy improved AHI, min <90% SaO_2_, and ESS scores vs baseline. - EPAP therapy may reduce symptoms of OSA and EDS in patients who do not adhere to PAP therapy. |
| De Mello-Fujita L, et al. Clinical predictors of CPAP discontinuation: a sham controlled study. *Sleep Med*. 2009. [Abstract] | 6-mo RCT | Patients with OSA randomized to CPAP (n=34) or sham (n=30) | Clinic-based | - BMI and AHI values were associated with refusal to initiate CPAP treatment; (*P*=0.02). |
| Rosenberg R, Doghramji P. Optimal treatment of obstructive sleep apnea and excessive sleepiness. *Adv Ther*. 2009;26:295-312. | Review | N/A | N/A | - Simple interventions and patient support can encourage CPAP adherence and successful OSA treatment. - Some patients may warrant surgical intervention. - In some patients, residual excessive sleepiness may occur, which may require adjunctive pharmacological treatment. |
| Rolfe I, et al. Long-term acceptance of continuous positive airway pressure in obstructive sleep apnea. *Am Rev Respir Dis*. 1991;144:1130-1133. | Retrospective chart review | Patients with OSA who initiated CPAP therapy (N=168) | Clinic-based | - Patients who continued CPAP treatment (24 to 52 mo) had improved symptoms of EDS. - More patients with severe EDS (79%) continued CPAP treatment vs those with mild (59%) or unequivocal (74%) EDS; (*P*<0.02). |

AHI, Apnea-Hypopnoea Index; BMI, body mass index; CGI-S, Clinical Global Impressions scale–Severity; CI, confidence interval; CPAP, continuous positive airway pressure; CVD, cardiovascular disease; EDS, excessive daytime sleepiness; EPAP, expiratory positive airway pressure; ESS, Epworth Sleepiness Scale; FOSQ, Functional Outcomes of Sleep Questionnaire; ICSD-2, International Classification of Sleep Disorders, second edition; IQR, interquartile range; MD, mean difference; min, minimum; MUC-S, Motivation to Use CPAP Scale; N/A, not available; OAm, mandibular advancement oral appliance; ODI, oxygen desaturation index; OR, odds ratio; OSA, obstructive sleep apnea; OSLER, Oxford Sleep Resistance test; PAP, positive airway pressure; PGO, patient’s global opinion; PSG, polysomnography; QoL, quality of life; RCT, randomized controlled trial; RDI, respiratory disturbance index; REM, rapid eye movement; SaO_2_, arterial oxygen saturation; SD, standard deviation; SF-36, Medical Outcomes Study 36-Item Short Form Health Survey; SpO_2_, peripheral blood oxygen saturation; WHOQoL-BREF, World Health Organization Quality of Life questionnaire, abbreviated version.

**Supplementary Table 10. Supporting evidence for Search 3B: How do you manage a patient who has residual EDS that is likely due to other reasons? What should be done to treat the other conditions? How does it differ from EDS due to OSA specifically?**

| **Supporting evidence for Search 3B: How do you manage a patient who has residual EDS that is likely due to other reasons? What should be done to treat the other conditions? How does it differ from EDS due to OSA specifically?** | | | | |
| --- | --- | --- | --- | --- |
| **Study** | **Article type/**  **Study design** | **Study population** | **Setting** | **Key finding(s)** |
| Aro MM, et al. Mood, sleepiness, and weight gain after three years on CPAP therapy for sleep apnoea. *Eur Clin Respir J.* 2021;8:1888394. | Long-term, observational trial | Patients with symptoms of OSA (N=223) | Clinic-based | - During the 3-year follow-up, patients treated with CPAP experienced a decrease in depressive symptoms and an improvement in sleepiness and sleep quality. |
| Baniak L, et al. The effect of CPAP use on insomnia among persons with type 2 diabetes and obstructive sleep apnea. *Am J Respir Crit Care Med.* 2019;199:A2648. [Abstract] | 12-wk, randomized, placebo-controlled trial | Patients with OSA and T2DM (N=71; active-CPAP, n=35; sham-CPAP, n=36) | Clinic-based | - A higher mean daily use of therapeutic CPAP correlated with a greater improvement in ISI total score at 12 wk from baseline (β=0.011; *P*=0.012). |
| Kim H, et al. Comorbidity of narcolepsy and obstructive sleep apnea: a case report. *Sleep Med Res.* 2018;9:124-127. | Case reports | Patients with narcolepsy and OSA (co-diagnosis) (N=3) | Clinic-based | - Patient 1: 78-year-old male diagnosed with narcolepsy with cataplexy and severe OSA was treated with venlafaxine and modafinil and CPAP.   - Cataplexy events resolved and ESS score improved from 14 to 6 after treatment with venlafaxine. - Patient 2: 28-year-old male diagnosed with narcolepsy with cataplexy and severe OSA was treated with modafinil but refused CPAP therapy for financial reasons.   - Lost to follow-up. - Patient 3: 38-year-old male diagnosed with narcolepsy without cataplexy and severe OSA was treated with modafinil, then switched to methylphenidate due to AEs.   - ESS score improved from 18 to 13 after methylphenidate and then to 11 after CPAP therapy. |
| Tietjens JR, et al. Obstructive sleep apnea in cardiovascular disease: a review of the literature and proposed multidisciplinary clinical management strategy. *J Am Heart Assoc.* 2019;8:e010440. | Review | Patients with OSA and CVD | N/A | - ASV (adaptive servoventilation) mode on CPAP therapy should not be used in patients with HF and low EF as it has been shown to increase CV mortality in these patients. |
| Muraki I, et al. Sleep apnea and type 2 diabetes. *J Diabetes Investig.* 2018;9:991-997. | Review | Patients with OSA and T2DM | N/A | - The Cardiovascular Health study showed that OSA symptoms such as EDS were associated with higher levels of fasting glucose levels, 2-hour glucose levels, and lower insulin sensitivity. - CPAP therapy for 2 mo reduced insulin resistance in patients with moderate to severe OSA, as assessed by the HOMA index. |
| El-Shabrawy M, et al. Treatment outcome and predictors of better response to thyroxin in hypothyroid patients with sleep apnea syndrome in Zagazig University hospital. *Egypt J Chest Dis Tuberculosis.* 2017;66:285-291. | RCT | Patients with OSA (N=130); 65 patients with hypothyroidism on thyroxin therapy,  65 euthyroid patients on CPAP therapy | Clinic-based | - Patients with hypothyroidism were more likely to have more frequent snoring (*P*<0.001). - Patients with hypothyroidism treated with thyroxin alone who reached a euthyroid state demonstrated significant improvement in sleep efficiency, snoring, index of desaturation, stage 3 duration, and respiratory disturbance index (all = *P*<0.001) compared to patients who did not respond to thyroxin therapy alone and needed CPAP therapy. |
| Sharples LD, et al. Meta-analysis of randomised controlled trials of oral mandibular advancement devices and continuous positive airway pressure for obstructive sleep apnoea-hypopnoea. *Sleep Med Rev.* 2016;27:108-124. | Review | Patients with OSA | N/A | - Mandibular advancement devices (MAD) result in a significant improvement in AHI; the effect was similar regardless of baseline AHI. - Improvement in AHI is approximately 3x greater with CPAP than MAD. |
| Epstein M, et al. Inaccuracy of apnea-hypopnea index determination by CPAP machines in patients with incompletely treated obstructive sleep apnea. *Chest*. 2015;148:1028A. [Abstract] | N/A | Patients with OSA suspected of having incompletely treated OSA (N=48) | At home | - CPAP machines reported an AHI within normal range for all 48 patients; however, 1 night of home sleep testing (simultaneous WatchPAT) demonstrated that 15 patients had an elevated AHI. - Home sleep testing may be useful in patients who are suspected to be incompletely treated for OSA. |
| Li Y, et al. Follow-up management of refractory continuous positive airway pressure therapy: emergent periodic limb movements in sleep. *Sleep Med.* 2014;15:1165-1167. | Case report | 48-year-old male with OSA and EDS who experiences CPAP-emergent periodic limb movement in sleep | N/A | - During 28-mo follow-up, patient was treated with single dopaminergic agents, single anticonvulsants, and combination drug therapies for PLMS. - The final therapy of levodopa/benserazide and carbamazepine successfully reduced PLMS and self-reported EDS, as assessed by ESS. |
| Launois SH, et al. On treatment but still sleepy: cause and management of residual sleepiness in obstructive sleep apnea. *Curr Opin Pulm Med.* 2013;19:601-608. | Review | Patients with OSA and residual sleepiness despite CPAP therapy | N/A | - After exclusion or treatment of other causes of sleepiness, stimulant therapy may be appropriate for patients with severe residual sleepiness, especially for people who operate motor vehicles. - Modafinil and armodafinil are additional treatment options for patients with residual EDS. |
| Rosenberg R, Doghramji P. Optimal treatment of obstructive sleep apnea and excessive sleepiness. *Adv Ther.* 2009;26:295-312. | Review | Patients with OSA and EDS | N/A | - Patient adherence to CPAP and lifestyle changes, such as weight loss, increased exercise, and smoking cessation, should be encouraged. - Adjunct pharmacological treatment for patients experiencing residual EDS with CPAP use should be considered; continue encouraging CPAP use in these patients. |
| Krystal AD, et al. Randomized, double-blind, placebo-controlled study of armodafinil in patients with residual excessive sleepiness associated with treated obstructive sleep apnea and comorbid depressive disorders. *Chest.* 2009;136:70S. [Abstract] | Randomized, double-blind, placebo-controlled, parallel-group trial | Patients with OSA and residual ES associated with CPAP-treated OSA and a comorbid depressive disorder (N=134) | Clinic-based | - Of the 134 patients, 92% of patients were diagnosed with comorbid MDD. - At baseline, mean (SD) scores were: ESS, 14.7 (3.2); FOSQ, 14.4 (2.9), and worst fatigue BFI, 6.7 (2.0). |
| Maski K, et al. Treatment of central disorders of hypersomnolence: an American Academy of Sleep Medicine clinical practice guideline. *J Clin Sleep Med.* 2021;17:1881-1893. | Practice guideline | N/A | N/A | - Recommended treatments for patients with narcolepsy are modafinil, pitolisant, sodium oxybate, solriamfetol (all strongly recommended), and armodafinil, dextroamphetamine, and methylphenidate (conditionally recommended). - Recommended treatments for patients with IH are modafinil (strongly recommended), clarithromycin, methylphenidate, pitolisant, and sodium oxybate (conditionally recommended). - Recommended treatment for Kleine-Levin syndrome is lithium (conditionally recommended). - Recommended treatments for hypersomnia secondary to alpha synucleinopathies are armodafinil, modafinil, and sodium oxybate (conditionally recommended). - Recommended treatments for post-traumatic hypersomnia are armodafinil and modafinil (conditionally recommended). - Recommended treatment for genetic disorders associated with primary CNS somnolence is modafinil (conditionally recommended). - Recommended treatment for hypersomnia secondary to brain tumors, infections, or other CNS lesions is modafinil (conditionally recommended). |
| Maski K, et al. Treatment of central disorders of hypersomnolence: an American Academy of Sleep Medicine systematic review, meta-analysis, and GRADE assessment. *J Clin Sleep Med.* 2021;17:1895-1945. | Review | N/A | N/A | - Recommended treatments for patients with narcolepsy include pitolisant, solriamfetol (high overall quality of evidence), armodafinil, l-carnitine, modafinil, selegiline, sodium oxybate, triazolam (moderate overall quality of evidence), clomipramine, dextroamphetamine, and methylphenidate (very low overall quality of evidence). - Recommended treatments for patients with IH include clarithromycin, modafinil (moderate), flumazenil, methylphenidate, pitolisant, and sodium oxybate (very low). - Recommended treatments for Kleine-Levin syndrome include lithium and methylprednisolone (both very low). - Recommended treatments for hypersomnia secondary to alpha synucleinopathies include modafinil, sodium oxybate (moderate), and armodafinil (very low). - Recommended treatments for post-traumatic hypersomnia include armodafinil and modafinil (moderate). - Recommended treatments for genetic disorders associated with primary CNS somnolence include methylphenidate, modafinil (moderate), and selegiline (low). - Recommended treatment for hypersomnia secondary to brain tumors, infections, or other CNS lesions include modafinil (very low). - Recommended treatment for hypersomnia secondary to endocrine disorder include liraglutide (very low). - Recommended treatment for hypersomnia associated with a psychiatric disorder include modafinil (moderate). |

AE, adverse event; AHI, Apnea-Hypopnoea Index; BFI, Brief Fatigue Inventory; CNS, central nervous system; CPAP, continuous positive airway pressure; CV, cardiovascular; CVD, cardiovascular disease; EDS, excessive daytime sleepiness; EF, ejection fraction; ES, excessive sleepiness; ESS, Epworth Sleepiness Scale; FOSQ, Functional Outcomes of Sleep Questionnaire; HF, heart failure; HOMA, homeostatic model assessment; IH, idiopathic hypersomnia; ISI, Insomnia Severity Index; MDD, major depressive disorder; N/A, not available; OSA, obstructive sleep apnea; PLMS, periodic limb movement in sleep; RCT, randomized controlled trial; SD, standard deviation; T2DM, type 2 diabetes mellitus.

**Supplementary Table 11. Supporting evidence for Search 3C: How do you distinguish between a diagnosis of EDS in OSA and OSA with comorbid idiopathic hypersomnia (IH)? Can a patient have a diagnosis of OSA with comorbid IH?**

| **Supporting evidence for Search 3C: How do you distinguish between a diagnosis of EDS in OSA and OSA with comorbid idiopathic hypersomnia (IH)? Can a patient have a diagnosis of OSA with comorbid IH?** | | | | |
| --- | --- | --- | --- | --- |
| **Study** | **Article type/**  **Study design** | **Study population** | **Setting** | **Key finding(s)** |
| Maski K, et al. Treatment of central disorders of hypersomnolence: an American Academy of Sleep Medicine clinical practice guideline. *J Clin Sleep Med.* 2021;171881-1893. | Practice guideline | N/A | N/A | - The recommended interventions for patients with IH differ from the recommended interventions for patients with hypersomnia due to other conditions. - Recommended treatments for patients with IH are modafinil (strongly recommended), clarithromycin, methylphenidate, pitolisant, and sodium oxybate (conditionally recommended). |
| Dauvilliers Y, et al. Measurement of symptoms in idiopathic hypersomnia: the Idiopathic Hypersomnia Severity Scale. *Neurology.* 2019;92:e1754-e1762. | Research study | Patients with untreated IH (n=210), treated IH (n=43), untreated narcolepsy type 1 (n=37); and controls (no sleepiness; n=73) | N/A | - The IHSS scoring system may be able to differentiate patients with IH from patients with narcolepsy type 1 and from patients without sleepiness.   - The IHSS total score correlated positively with ESS scores in both untreated and treated patients with IH and in patients without sleepiness. - Treatments used for patients with IH included modafinil, methylphenidate, and pitolisant. |
| Maski K, et al. Treatment of central disorders of hypersomnolence: an American Academy of Sleep Medicine systematic review, meta-analysis, and GRADE assessment. *JCSM.* 2021;17:1895-1945. | Review | N/A | N/A | - Recommended treatments for patients with IH include clarithromycin, modafinil (moderate), flumazenil, methylphenidate, pitolisant, and sodium oxybate (very low). |
| Dauvilliers Y. Differential diagnosis in hypersomnia. *Curr Neurol Neurosci Rep.* 2006;6:156-162. | Review | N/A | N/A | - PSG plus MSLT are required to diagnose IH, assess the objective EDS, and rule out other causes of hypersomnia such as sleep apnea syndrome. - IH is defined as a normal or prolonged nocturnal sleep episode associated with EDS.   - Symptoms include: constant EDS and unwanted naps, interrupted night sleep, and difficult morning awakening. |

EDS, excessive daytime sleepiness; ESS, Epworth Sleepiness Scale; IH, idiopathic hypersomnia; IHSS, Idiopathic Hypersomnia Severity Scale; MSLT, Multiple Sleep Latency test; N/A, not available; OSA, obstructive sleep apnea; PSG, polysomnography.

**Supplementary Table 12. Supporting evidence for Search 4: When/how should pharmacologic treatment for EDS in OSA be initiated? Which clinical considerations and/or patient-specific factors should guide decision making related to initiation of pharmacologic treatment? Are there concerns that would lead you to consider implementing pharmacotherapy earlier in some patients (eg, driving risk, patient worries about losing their job)? What metrics should be used to determine success of a pharmacologic treatment?**

| **Supporting evidence for Search 4: When/how should pharmacologic treatment for EDS in OSA be initiated? Which clinical considerations and/or patient-specific factors should guide decision making related to initiation of pharmacologic treatment? Are there concerns that would lead you to consider implementing pharmacotherapy earlier in some patients (eg, driving risk, patient worries about losing their job)? What metrics should be used to determine success of a pharmacologic treatment?** | | | | | |
| --- | --- | --- | --- | --- | --- |
| **Study** | **Article type/**  **Study design** | **Study population** | **Setting** | **Treatment(s) assessed** | **Key finding(s)** |
| Pepin JL, et al. Pitolisant for residual excessive daytime sleepiness in OSA patients adhering to CPAP: a randomized trial. *Chest.* 2021;159(4):1598-1609. | 12-wk, phase 3, double-blind, randomized, placebo-controlled, parallel-design trial | Patients with OSA and EDS who had been treated with CPAP for ≥3 mo (usage ≥4 h/night) (N=244; pitolisant, n=183; placebo, n=61) | Clinic-based | Pitolisant | - Pitolisant was effective in improving self-reported EDS, as assessed by ESS mean (95% CI) change from baseline to end of treatment: pitolisant, –5.5 (–6.2, –4.9); placebo, –2.8 (–4.3, –1.2); (*P<*0.001).   - No significant effect on ability to maintain wakefulness, as assessed by the OSLER, was found (*P*=0.05). - The frequency of treatment-related TEAEs was similar between pitolisant- and placebo-treated patients. - No changes from baseline were found in SBP, DBP, or HR; however, 4 pitolisant-treated patients (2.2%) had ≥1 post-dose QTcF interval >450 msec, and 6 pitolisant-treated patients (3.3%) had 1 QTcF elongation ≥60 msec compared with 2 (3.3%) and 3 (4.9%) placebo-treated patients, respectively. |
| Rosenberg R, et al. Clinically relevant effects of solriamfetol on excessive daytime sleepiness: a posthoc analysis of the magnitude of change in clinical trials in adults with narcolepsy or obstructive sleep apnea. *J Clin Sleep Med.* 2021;17(4):711-717. | Two 12-wk, phase 3, randomized, placebo-controlled, parallel-design trial | Patients with narcolepsy or OSA and EDS with a baseline ESS score ≥10  OSA (solriamfetol, n=345; placebo, n=114) | Clinic-based | Solriamfetol | - A higher percentage of patients treated with solriamfetol at wk 12 vs placebo were considered treatment responders, as assessed by a reduction in ESS score ≥25% from baseline: 37.5 mg, 50.0%; 75 mg, 55.2%; 150 mg, 81.9%; 300 mg, 75.7%; placebo, 36.8%. - The majority of TEAEs were mild to moderate in severity and were generally more frequent with higher doses (150 mg and 300 mg) of solriamfetol. |
| Schweitzer PK, et al. Randomized controlled trial of solriamfetol for excessive daytime sleepiness in OSA: An analysis of subgroups adherent or nonadherent to OSA treatment. *Chest.* 2021;160(1):307-318. | 12-wk, phase 3, double-blind, randomized, placebo-controlled, parallel-design trial | Patients with OSA and EDS with current or prior use of a primary OSA therapy (N=459; solriamfetol, n=345; placebo, n=114) | Clinic-based | Solriamfetol | - Solriamfetol increased MWT sleep latency and decreased ESS scores in a dose-dependent manner compared to placebo in patients both adherent and nonadherent to OSA treatment.   - LS mean difference from placebo in MWT sleep latency change from baseline to wk 12 in adherent vs nonadherent patients: 37.5 mg, 3.7 vs 4.8; 75 mg, 8.4 vs 9.9; 150 mg, 10.2 vs 11.9; 300 mg, 12.5 vs 13.5.   - LS mean difference from placebo in ESS change from baseline to wk 12 in adherent vs nonadherent patients: 37.5 mg, −2.4 vs −0.7; 75 mg, −1.3 vs −2.6; 150 mg, −4.2 vs −5.0; 300 mg, −4.7 vs −4.6. - TEAEs across both subgroups were mild to moderate in severity and were generally dose-dependent. - At wk 12, solriamfetol was associated with small mean increases from baseline in SBP, DBP, or HR compared to placebo in both subgroups. |
| Schweitzer PK, et al. Effects of solriamfetol in a long-term trial of participants with obstructive sleep apnea who are adherent or nonadherent to airway therapy. *J Clin Sleep Med.* 2021;17(4):659-668. | Phase 3, open-label trial with a randomized withdrawal period | Patients with OSA and EDS with either use of a primary OSA therapy, history of an OSA primary therapy use attempt, or history of surgical intervention to treat OSA symptoms (N=417; group A, n=333; group B, n=84)  Group A: patients who enrolled immediately after completion of a parent study  Group B: patients who enrolled at a later time | Clinic-based | Solriamfetol | - Patients who continued solriamfetol therapy after immediate completion of a prior parent study demonstrated sustained improvement in EDS (wk 40 of the open-label study), as assessed by ESS, regardless of adherence to OSA primary therapy. - The frequency of TEAEs was similar among patients who were adherent and nonadherent to OSA primary therapy. |
| Weaver TE, et al. Determination of thresholds for minimally important difference and clinically important response on the functional outcomes of sleep questionnaire short version in adults with narcolepsy or obstructive sleep apnea. *Sleep Breath.* 2021;25(3):1707-1715. | 12-wk, phase 3, double-blind, randomized, placebo-controlled trial | Patients with narcolepsy or OSA and EDS  OSA: patients with current or prior use of a primary OSA therapy, including a CPAP machine, oral appliance, or surgical intervention (n=459) | Clinic-based | Solriamfetol | - The minimally important difference and clinically important response estimates on the FOSQ-10 total score were 1.8 and 2.2 points, respectively. |
| Dauvilliers Y, et al. Pitolisant for daytime sleepiness in patients with obstructive sleep apnea who refuse continuous positive airway pressure treatment: a randomized trial. *Am J Respir Crit Care Med.* 2020;201(9): 1135-1145. | 12-wk, phase 3, double-blind, randomized, placebo-controlled, parallel-design trial | Patients with moderate to severe OSA and EDS (ESS score ≥12), refusing CPAP treatment, and without significant CV disease (N=268; pitolisant, n=201; placebo, n=67) | Clinic-based | Pitolisant | - Pitolisant significantly reduced ESS scores from baseline to end of intervention compared to placebo: –6.3 vs −3.6 points; *P<*0.001.   - No significant effect on ability to maintain wakefulness (assessed by OSLER) was seen (*P*=0.108). - The frequency of treatment-related TEAEs was similar between pitolisant- and placebo-treated patients (24.0% and 19.4%, respectively). - No changes from baseline were found in SBP, DBP, or HR; however, 3 pitolisant-treated patients (1.5%) had ≥1 post-dose QTcF interval >450 msec, 4 pitolisant-treated patients (2.0%) had 1 QTcF elongation ≥60 msec, and 1 placebo-treated patient had 1 QTcF interval >450 msec. |
| Malhotra A, et al. Long-term study of the safety and maintenance of efficacy of solriamfetol (JZP-110) in the treatment of excessive sleepiness in participants with narcolepsy or obstructive sleep apnea. *Sleep.* 2020;43(2):zsz220. | Phase 3, open-label trial with a double-blind, placebo-controlled randomized withdrawal period | Patients with narcolepsy or OSA and EDS  OSA: patients with current or prior use of a primary OSA therapy (n=417)  Group A: patients who enrolled immediately after completion of a phase 3, 12-wk study  Group B: patients who enrolled after completion of a phase 2 study or a phase 3, 6-wk study | Clinic-based | Solriamfetol | - Patients who continued solriamfetol therapy (≤52 wk) demonstrated sustained improvement in EDS, as assessed by ESS.   - In group A, 81.7% of patients had ESS ≤10 at wk 40 compared with 6.0% of patients at parent study baseline.   - In group B, 84.5% of patients had ESS scores ≤10 at wk 52 compared with 11.9% of patients at current study baseline. - Most TEAEs were mild or moderate and mostly occurred during the first 2 wk of the study. - 9 patients had CV or potential CV serious TEAEs; of these, 2 were considered treatment related: atrial fibrillation in 1 patient who had concomitant administration of thyroid medications and CVA in 1 patient with a history of hypertension. |
| Malhotra RK. Pro-con debate: use of wake-promoting agents for the treatment of daytime fatigue in OSA patients with curtailed CPAP use (less than 6 h). *Curr Sleep Med Rep.* 2020;6(3):184-188. | Review | Patients with OSA and residual EDS despite PAP therapy | N/A | Wake-promoting agents - modafinil, armodafinil, and solriamfetol | - After careful assessment of patient symptoms, consider adding a wake-promoting agent in conjunction with PAP therapy for symptomatic benefit.   - ie, if patient is suffering from drowsy driving or has difficulties maintaining alertness at work or school. - There are risks associated with these agents, especially in patients with underlying cardiac disease or risk factors for cardiac disease   - WPAs are known to increase BP, trigger cardiac arrhythmias, or possibly worsen coronary artery disease. |
| Weaver TE, et al. Effects of solriamfetol on quality-of-life measures from a 12-week phase 3 randomized controlled trial. *Ann Am Thorac Soc.* 2020;17(8):998-1007. | 12-wk, phase 3, double-blind, randomized, placebo-controlled, parallel-design trial | Patients with OSA and EDS with current or prior use of a primary OSA therapy (N=459; solriamfetol, n=345; placebo, n=114) | Clinic-based | Solriamfetol | - Solriamfetol at 150 mg and 300 mg improved daily functioning, HRQoL, and work productivity compared with placebo. |
| Strollo PJ, et al. Solriamfetol for the treatment of excessive sleepiness in OSA: A placebo-controlled randomized withdrawal study. *Chest.* 2019;155(2):364-374. | 6-wk, phase 3 trial with a double-blind, placebo-controlled randomized withdrawal period | Patients with OSA and EDS with current or prior use of a primary OSA therapy (N=174) | Clinic-based | Solriamfetol | - After 4 wk of treatment, solriamfetol increased MWT mean sleep latency and decreased ESS scores compared to placebo. - During the randomized withdrawal period (wks 4 to 6; n=124), patients who continued solriamfetol maintained their efficacy, whereas patients who switched to placebo had worsened MWT and ESS scores.   - LS mean change in MWT sleep latency was −1.0 min with solriamfetol compared with −12.1 min with placebo; LS mean difference, 11.2 min (*P*<0.0001).   - LS mean change in ESS score was −0.1 point for solriamfetol and 4.5 points for placebo; LS mean difference was −4.6 (*P*<0.0001). - There was no evidence of rebound hypersomnia or withdrawal effects after abrupt discontinuation of solriamfetol in patients randomized to placebo. |
| Abad VC, Guilleminault C. Solriamfetol for the treatment of daytime sleepiness in obstructive sleep apnea. *Expert Rev Respir Med.* 2018;12(12):1007-1019. | Review | Patients with OSA and residual EDS despite primary OSA therapy | N/A | Solriamfetol | - Solriamfetol can be considered as adjunctive therapy for patients with OSA and EDS. - As a relatively newer agent, physicians may be more likely to prescribe solriamfetol in patients with OSA who have failed other stimulants, but it may emerge as first-line therapy with increased clinical experience and use. |
| Chapman JL, et al. Does armodafinil improve driving task performance and weight loss in sleep apnea? A randomized trial. *Am J Resp Crit Care Med.* 2018;198(7):941-950. | 6-mo, double-blind, randomized, placebo-controlled, parallel-design trial | Patients with OSA and EDS who are overweight and had rejected standard treatment (N=113; armodafinil, n=55; placebo, n=58) | Clinic-based | Armodafinil | - Steering deviation from the median lane position in the final 30 min of a 90-min afternoon drive at 6 mo was not better with armodafinil compared to placebo.   - There was no interaction between diet and treatment for this primary outcome. - Patients in the armodafinil arm experienced higher risk of serious AEs and AEs leading to withdrawal. - In conjunction with diet, SBP and DBP were reduced in patients treated with armodafinil and placebo. |
| Gurubhagavatula I, et al. Management of obstructive sleep apnea in commercial motor vehicle operators: recommendations of the AASM Sleep and Transportation Safety Awareness Task Force. *J Clin Sleep Med.* 2017;13(5):745-758. | Practice guideline | Commercial motor vehicle operators with OSA | N/A | Primary OSA therapy | - Guidelines for screening for OSA in commercial motor vehicle operators have been proposed by several entities - The article recommends that drivers who meet the following 3 criteria be considered high-risk individuals who should be referred to a board-certified sleep medicine specialist for clinical sleep evaluation and diagnostic testing: - Individuals with a BMI ≥40 kg/m^2^. - Individuals who have admitted fatigue or sleepiness during the duty period OR who have been involved in a sleepiness-related crash or accident. - Individuals with a BMI ≥33 kg/m^2^ and either hypertension requiring ≥2 medications for control of type 2 diabetes. |
| Inoue Y, et al. Findings of the maintenance of wakefulness test and its relationship with response to modafinil therapy for residual excessive daytime sleepiness in obstructive sleep apnea patients adequately treated with nasal continuous positive airway pressure. *Sleep Med.* 2016;27-28:45-48. | 4-wk, double-blind, randomized, placebo-controlled, parallel-design trial | Patients with OSA and EDS receiving optimal CPAP therapy (N=50) | Clinic-based | Modafinil | - No significant correlation was found between subjective (MWT sleep latency) and objective (ESS) sleepiness in patients with EDS. - In patients with mean baseline MWT <14 min, the improvements of subjective (MWT sleep latency) and objective (ESS) sleepiness were significantly greater in patients treated with modafinil compared to placebo. |
| Chapman JL, et al. Modafinil improves daytime sleepiness in patients with mild to moderate obstructive sleep apnoea not using standard treatments: a randomised placebo-controlled crossover trial. *Thorax.* 2014;69(3):274-279. | Double-blind, randomized, placebo-controlled, crossover trial | Patients with untreated mild to moderate OSA (no prior treatment with CPAP or mandibular advancement splint within the past 3 mo) and EDS (N=32) | Clinic-based | Modafinil | - During the 2-wk treatment period, modafinil was effective in improving ESS scores (mean improvement [95% CI] compared to placebo: 3.6 points [1.3–5.8]). - The relative risk of experiencing an AE on modafinil compared to placebo was 2.7 (95% CI 1.4–5.3; *P*=0.003); these AEs were primarily transient and mild in severity. - There was no significant change in BP between baseline and end of treatment in patients treated with modafinil and placebo. |
| Greve DN, et al. Effect of armodafinil on cortical activity and working memory in patients with residual excessive sleepiness associated with CPAP-treated OSA: a multicenter fMRI study. *J Clin Sleep Med.* 2014;10(2):143-153. | 2-wk, double-blind, randomized, placebo-controlled, parallel-design trial | Patients with OSA and residual excessive sleepiness despite stable use of CPAP (usage ≥4 h/night on ≥70% of nights) (N=40; armodafinil, n=21; placebo, n=19) | Clinic-based | Armodafinil | - Armodafinil did not improve fMRI-measured task-related prefrontal cortex activation. |
| Aarts MCJ, et al. Remarkable differences between three evidence‐based guidelines on management of obstructive sleep apnea‐hypopnea syndrome. *Laryngoscope.* 2013;123(1):283-291. | Review | Patients with OSA | N/A | N/A | - This review concluded there are remarkable disagreements between evidence-based guidelines within the same clinical area. |
| Inoue Y, et al. Efficacy and safety of adjunctive modafinil treatment on residual excessive daytime sleepiness among nasal continuous positive airway pressure-treated Japanese patients with obstructive sleep apnea syndrome: a double-blind placebo-controlled study. *J Clin Sleep Med.* 2013;9(8):751-757. | 4-wk, double-blind, randomized, placebo-controlled, parallel-design trial | Patients with OSA and residual EDS despite optimal nasal CPAP therapy (N=114; modafinil, n=52; placebo, n=62) | Clinic-based | Modafinil | - Modafinil was effective in improving self-reported EDS, as assessed by ESS (LS mean change from baseline to end of treatment: modafinil, −6.61; placebo, −2.44; *P<*0.001), and objective EDS, as assessed by MWT sleep latency (LS mean change from baseline to end of treatment: modafinil, 2.8 min; placebo, −0.40 min; *P=*0.009). - There were no significant differences in the rate of AEs between the groups (*P=*0.146), and no patients withdrew from the study due to any AEs. |
| Kay GG, Feldman N. Effects of armodafinil on simulated driving and self-report measures in obstructive sleep apnea patients prior to treatment with continuous positive airway pressure. *J Clin Sleep Med.* 2013;9(5):445-454. | Single-site, double-blind, randomized, placebo-controlled trial | Newly diagnosed patients with OSA and EDS prior to CPAP therapy (N=69; armodafinil, n=35; placebo, n=34) | Clinic-based | Armodafinil | - Armodafinil significantly improved patients’ Driving Safety Score (DSS) vs placebo (*P*=0.03).   - Of the 7 components of the DSS, significant differences were seen in Out of Lane (*P*=0.02) and Lane Deviation (*P*=0.002) scenarios. - There was a trend toward improved self-reported sleepiness, as assessed by the ESS in patients treated with armodafinil (*P*=0.066), which significantly improved following CPAP therapy (*P*<0.0001). - The frequency of treatment-related, TEAEs was similar between armodafinil- and placebo-treated patients. |
| Qaseem A, et al. Management of obstructive sleep apnea in adults: a clinical practice guideline from the American College of Physicians. *Ann Intern Med.* 2013;159(7):471-483. | Practice guideline | Patients with OSA | N/A | Primary OSA therapy | - CPAP is more effective than control or sham CPAP. - Fixed and auto-CPAP have overall similar efficacy and adherence despite small differences, and low-quality evidence showed that C-Flex and fixed CPAP were similarly efficacious. - CPAP more effectively reduced AHI and arousal index scores and increased the minimum oxygen saturation compared with MADs. |
| Black JE, et al. The long-term tolerability and efficacy of armodafinil in patients with excessive sleepiness associated with treated obstructive sleep apnea, shift work disorder, or narcolepsy: an open-label extension study. *J Clin Sleep Med.* 2010;6(5):458-466. | Open-label extension study of four 12-wk, double-blind, placebo-controlled trials | Patients with narcolepsy, SWD, or OSA and excessive sleepiness  OSA: patients who are adherent with CPAP (usage ≥4 h/night on ≥70% of nights) (n=474) | Clinic-based | Armodafinil | - 18% of patients discontinued treatment due to AEs, including palpitations and hypertension.   - Cardiac and vascular AEs were reported in 10% and 9% of patients, respectively.   - Modest increases in BP and HR were reported from baseline to final visit. - Improvements on the CGI-C and mean changes on the ESS and BFI suggest that efficacy was maintained in patients treated with armodafinil.   - At final visit (≥12 mo), mean ESS score was reduced by 6.4 points. |
| Rosenberg R, Bogan R. Armodafinil in the treatment of excessive sleepiness. *Nat Sci Sleep.* 2010;2:95-105. | Review | Patients with narcolepsy, SWD, or OSA and ES | N/A | Armodafinil | - In a combined analysis of 2 phase 3 trials comparing armodafinil and placebo, MWT sleep latency increased by 2.0 min in patients treated with armodafinil vs decreased by 1.5 min in patients treated with placebo (*P*<0.0001). - Armodafinil was generally well tolerated in randomized trials and open-label extension studies - The most common AEs were headache, nasopharyngitis, anxiety, and insomnia. |
| Schwartz JRL, et al. Tolerability and efficacy of armodafinil in naïve patients with excessive sleepiness associated with obstructive sleep apnea, shift work disorder, or narcolepsy: a 12-month, open-label, flexible-dose study with an extension period. *J Clin Sleep Med.* 2010;6(5):450-457. | 12-mo, open-label study | Patients with narcolepsy, SWD, or OSA and ES  OSA: patients who are adherent with CPAP (usage ≥4 h/night on ≥70% of nights) (n=170) | Clinic-based | Armodafinil | - Armodafinil administered for ≥12 mo improved the overall clinical condition of the patient and ESS scores.   - At the final visit, 80% of patients were rated as at least minimally improved on the CGI-I and 68% were rated as much or very much improved.   - From baseline to final visit, mean (SD) ESS decreased by 7.3 (5.6) points (95% CI, −8.39, −6.30). - 16% of patients withdrew from the 12-mo study due to AEs. |
| Williams SC, et al. Modafinil effects during acute continuous positive airway pressure withdrawal: a randomized crossover double-blind placebo-controlled trial. *Am J Respir Crit Care Med.* 2010;181(8):825-831. | Double-blind, randomized, placebo-controlled, crossover trial | Patients with OSA and EDS with acute withdrawal of CPAP (N=21) | Clinic-based | Modafinil | - Modafinil treatment during acute withdrawal of CPAP significantly improved (*P*<0.01) simulated driving performance, neurocognitive performance, and self-reported sleepiness. |
| Epstein LJ, et al. Clinical guideline for the evaluation, management and long-term care of obstructive sleep apnea in adults. *J Clin Sleep Med.* 2009;5(3):263-276. | Practice guideline | Patients with OSA | N/A | Modafinil | - Modafinil is recommended for the treatment of EDS in patients with OSA who have residual sleepiness despite effective PAP therapy. |
| Weaver TE, et al. Modafinil improves functional outcomes in patients with residual excessive sleepiness associated with CPAP treatment. *J Clin Sleep Med.* 2009;5(6):499-505. | Two double-blind, randomized, placebo-controlled trials | Patients with OSA and EDS despite CPAP use (N=480, modafinil, n=292; placebo, n=188) | Clinic-based | Modafinil | - A greater proportion of patients treated with modafinil were considered responders compared with those treated with placebo (45% vs 25%; *P*<0.001).   - Responders were patients who experienced an increase in FOSQ Total score by ≥2 points and each individual domain score by ≥1 point. |
| Bittencourt LRA, et al. Placebo and modafinil effect on sleepiness in obstructive sleep apnea. *Prog Neuropsychopharmacol Biol Psychiatry*. 2008;32(2):552-559. | 4-wk, double-blind, randomized, placebo-controlled, parallel-design trial | Patients with OSA and EDS receiving CPAP (N=20; modafinil, n=9; placebo, n=11) | Clinic-based | Modafinil | - During the treatment period, modafinil significantly reduced mean (SD) ESS scores from 15.2 (6.1) to 7.8 (7.2); *P*=0.0006. - Based on ESS scores, there was a placebo effect seen in patients prior to randomization. |
| Roth T, et al. Armodafinil improves wakefulness and long-term episodic memory in nCPAP-adherent patients with excessive sleepiness associated with obstructive sleep apnea. *Sleep Breath.* 2008;12(1):53-62. | Two 12-wk, multicenter, double-blind, randomized, placebo-controlled, parallel-design trials | Patients with OSA and EDS receiving stable and effective nCPAP (usage ≥4 h/night on ≥70% of nights) (N=658; armodafinil, n=395; placebo, n=263) | Clinic-based | Armodafinil | - Armodafinil increased mean MWT sleep latency from baseline to final visit vs placebo: (2.0 min vs −1.5 min; *P*<0.0001). - Armodafinil significantly improved patients’ ability to engage in activities of daily living as assessed by ESS (*P*<0.0001) and global fatigue (*P*<0.01) at all visits compared to placebo. - There were no clinically significant differences for laboratory values, EKG parameters, or physical exam between groups. |
| Williams SC, et al. The effect of modafinil following acute CPAP withdrawal: a preliminary study. *Sleep Breath*. 2008;12(4):359-364. | Double-blind, randomized, placebo-controlled, crossover trial | Patients with OSA and EDS with acute withdrawal of CPAP (N=12) | Clinic-based | Modafinil | - Modafinil treatment after acute withdrawal of CPAP did not alter neurobehavioral performance compared to placebo. - However, modafinil improved self-reported sleepiness, as assessed by SSS (*P*=0.03) and KSS (*P=*0.04), after CPAP withdrawal compared to placebo. |
| Hirshkowitz M, et al. Adjunct armodafinil improves wakefulness and memory in obstructive sleep apnea/hypopnea syndrome. *Respir Med.* 2007;101(3):616-627. | 12-wk, double-blind, randomized, placebo-controlled, parallel-design trial | Patients with OSA and EDS receiving stable and effective nCPAP (usage ≥4 h/night on ≥70% of nights) (N=259; armodafinil, n=129; placebo, n=130) | Clinic-based | Armodafinil | - Armodafinil increased mean MWT sleep latency from baseline to final visit vs placebo: (2.3 min vs −1.3 min; *P*=0.0003). - Armodafinil significantly improved patients’ clinical condition on the CGI-C scale (*P*=0.0069), episodic secondary memory (*P*=0.0102), ESS scores (*P*<0.01), and global fatigue (*P*<0.05) compared to placebo. - There were no statistically significant or clinically meaningful differences in SBP, DBP, or HR between groups. |
| Hirshkowitz M, Black J. Effect of adjunctive modafinil on wakefulness and quality of life in patients with excessive sleepiness-associated obstructive sleep apnoea/ hypopnoea syndrome: a 12-month, open-label extension study. *CNS Drugs.* 2007;21(5):407-416. | 12-mo open-label extension study of a 12-wk, double-blind, randomized, placebo-controlled trial | Patients with OSA and EDS receiving continued CPAP therapy (N=266) | Clinic-based | Modafinil | - Modafinil maintained wakefulness, functioning, and quality of life.   - Mean ESS score at 3, 6, 9, and 12 mo significantly improved compared with baseline (*P*<0.0001).   - Mean FOSQ total score at 6 and 12 mo significantly improved compared with baseline (*P*<0.0001).   - Mean SF-36 scores at 6 and 12 mo significantly improved compared with baseline (*P*<0.05). - 36 patients withdrew due to AEs, most commonly: anxiety, insomnia, and nervousness. - There was a clinically significant increase in BP in 6 patients, 5 of whom had a history of hypertension. |
| Roth T, et al. Evaluation of the safety of modafinil for treatment of excessive sleepiness. *J Clin Sleep Med.* 2007;3(6):595-602. | 6 double-blind, randomized, placebo-controlled trials (2 in OSA) | Patients with narcolepsy, SWD, or OSA and ES  OSA (modafinil, n=295; placebo, n=189) | Clinic-based | Modafinil | - Modafinil was well tolerated. - Headache was the most common AE with modafinil, and was associated with a dose-related effect. - Modafinil was not associated with clinically significant changes in SBP, DBP, and HR. |
| Roth T, et al. Effects of armodafinil in the treatment of residual excessive sleepiness associated with obstructive sleep apnea/hypopnea syndrome: a 12-week, multicenter, double-blind, randomized, placebo-controlled study in nCPAP-adherent adults. *Clin Ther.* 2006;28(5):689-706. | 12-wk, double-blind, randomized, placebo-controlled, parallel-design trial | Patients with OSA and EDS despite effective CPAP use (N=395, armodafinil 250 mg, n=131; armodafinil 150 mg, n=133; placebo, n=131) | Clinic-based | Armodafinil | - Mean change from baseline in MWT sleep latency at wks 4, 8, 12, and final visit indicated significant improvement in the armodafinil 150 mg and 250 mg groups compared with placebo (change was similar between armodafinil 150 mg and 250 mg). - The most common AEs that led to discontinuation in patients treated with armodafinil were headache and nausea. |
| Black JE, Hirshkowitz M. Modafinil for treatment of residual excessive sleepiness in nasal continuous positive airway pressure-treated obstructive sleep apnea/ hypopnea syndrome. *Sleep*. 2005;28(4):464-471. | 12-wk, double-blind, randomized, placebo-controlled, parallel-design trial | Patients with OSA and EDS despite nCPAP therapy (N=309, modafinil 200 mg, n=104; modafinil 400 mg, n=101; placebo, n=104) | Clinic-based | Modafinil | - In modafinil-treated patients, mean MWT sleep latency increased by 1.6 min (200 mg) and 1.5 min (400 mg) at wk 12 from baseline compared to a decrease by 1.1 min in patients receiving placebo (*P*<0.0001). - Modafinil decreased ESS scores by 4.5 points (both groups) at wk 12 compared to a 1.8-point decrease for placebo (*P*<0.0001). - There were no changes in mean SBP and DBP during the treatment period. |
| Dinges DF, Weaver TE. Effects of modafinil on sustained attention performance and quality of life in OSA patients with residual sleepiness while being treated with nCPAP. *Sleep Med.* 2003;4(5):393-402. | Double-blind, randomized, placebo-controlled, parallel-design trial | Patients with OSA and EDS despite nCPAP therapy for at least 2 mo (N=157, modafinil, n=77; placebo, n=80) | Clinic-based | Modafinil | - Modafinil significantly improved behavioral alertness, as assessed by the PVT: decrease in number of lapses (transformed; *P=*0.010), increase in the reciprocal of the 10% slowest reaction times (RTs) (*P=*0.023), and decrease in median RT (*P=*0.010). - Modafinil significantly improved FOSQ total scores at wks 1 and 4 from baseline compared with placebo (*P*<0.05). |
| Schwartz JRL, et al. Modafinil as adjunct therapy for daytime sleepiness in obstructive sleep apnea: a 12-week, open-label study. *Chest.* 2003;124(6): 2192-2199. | 12-wk, open-label study of patients who completed a 4-wk, double-blind, placebo-controlled trial | Patients with OSA and EDS despite effective nCPAP therapy (N=125) | Clinic-based | Modafinil | - Improvements in wakefulness, as assessed by ESS, demonstrated during the 12-wk, double-blind treatment period were maintained throughout the 12 wk of the open-label study. - 8 patients reported CV AEs, including hypertension, palpitations, and tachycardia. |
| Kingshott RN, et al. Randomized, double-blind, placebo-controlled crossover trial of modafinil in the treatment of residual excessive daytime sleepiness in the sleep apnea/hypopnea syndrome. *Am J Respir Crit Care Med.* 2001;163(4):918-923. | 7-wk, double-blind, randomized, placebo-controlled, crossover trial | Patients with OSA and EDS despite CPAP therapy (N=32) | Clinic-based | Modafinil | - There were no significant improvements in self-reported sleepiness (ESS), QoL (FOSQ, SF-36), or cognitive performance. - There was significant improvement in objective sleepiness (MWT). |
| Pack AI, et al. Modafinil as adjunct therapy for daytime sleepiness in obstructive sleep apnea. *Am J Respir Crit Care Med.* 2001;164(9):1675-1681. | 4-wk, double-blind, randomized, placebo-controlled, parallel-design trial | Patients with OSA and EDS despite effective nCPAP therapy (N=157; modafinil, n=77; placebo, n=80) | Clinic-based | Modafinil | - Greater improvements in mean change from baseline in ESS scores at wks 1 and 4 were demonstrated with modafinil compared to placebo (*P*<0.001).   - Mean improvement from baseline in ESS scores was significantly different between the treatment groups at wk 4 for patients with baseline ESS score of 10 to 14 (*P*<0.01). - The most common TEAEs associated with modafinil treatment were headache and nervousness. |
| Arnulf I, et al. Modafinil in obstructive sleep apnea-hypopnea syndrome: a pilot study in 6 patients. *Respiration.* 1997;64(2):159-161. | 5-wk, double-blind, randomized, placebo-controlled, crossover trial | Patients with OSA and EDS (N=6) | Clinic-based | Modafinil | - The daytime PSG demonstrated a 42% reduction in daytime sleep duration (mean [SD]): modafinil, 45 (36) min; placebo, 77 (41) min; (*P*<0.05). - Modafinil improved duration of alert wake during the daytime by 1 h: modafinil, 18.7 (1.2) h; placebo, 17.7 (1.2) h; (*P*<0.01). - No patient discontinued treatment and no differences were found between groups in cardiac events. |
| Wang J, et al. Efficacy and safety of solriamfetol for excessive sleepiness in narcolepsy and obstructive sleep apnea: findings from randomized controlled trials. *Sleep Med.* 2021;79:40-47. | Review | Patients with narcolepsy or OSA and ES | N/A | Solriamfetol | - MWT mean sleep latency improved by 9.52 min with solriamfetol relative to placebo   - Data from separate meta-analyses showed improvement of 2.29 min with modafinil/armodafinil, and 4.48 min with pitolisant 40 mg/d. - ESS score decreased by 3.74 points with solriamfetol, compared with placebo.   - Data from separate meta-analyses showed decreases of 2.21 points with modafinil/armodafinil and 3.05 points with pitolisant. |
| Strollo PJ, et al. Cardiovascular effects during solriamfetol treatment in phase 3 trials in obstructive sleep apnea. *Am J Respir Crit Care Med.* 2020;201:A6257. [Abstract] | 12-wk, randomized, placebo-controlled trial and an open-label extension study | Patients with OSA and EDS (randomized: N=474; solriamfetol, n=355; placebo, n=119; OLE: N=417) | Clinic-based | Solriamfetol | - During the 12-wk trial and OLE, patients treated with solriamfetol demonstrated small mean changes in BP and HR. - Few solriamfetol-treated patients in both the 12-wk study (1.1%) and the OLE (1.9%) initiated new antihypertensive medication. |
| Subedi R, et al. Efficacy and safety of solriamfetol for excessive daytime sleepiness in narcolepsy and obstructive sleep apnea: a systematic review and meta-analysis of clinical trials. *Sleep Med.* 2020;75:510-521. | Review | Patients with narcolepsy or OSA and EDS | N/A | Solriamfetol | - Use of solriamfetol resulted in a significant improvement in MWT sleep latency by 9.93 min relative to placebo. - This meta-analysis showed ESS score significantly reduced by 4.44 points with solriamfetol compared with sodium oxybate, modafinil, and pitolisant. |
| Weaver TE, et al. Relationship between sleep efficacy endpoints and measures of functional status and health-related quality of life in participants with narcolepsy or obstructive sleep apnea treated for excessive daytime sleepiness. *J Sleep Res.* 2021;30(3):e13210. | 12-wk, double-blind, RCT | Patients with OSA and EDS (N=459) | Clinic-based | Solriamfetol | - Change in ESS score was highly correlated with change in the FOSQ-10 total score (−0.541; *P*<0.001) and moderately correlated with change in MWT (−0.328; *P*<0.001). |
| Carlton R, et al. Healthcare costs among patients with excessive sleepiness associated with obstructive sleep apnea, shift work disorder, or narcolepsy. *Am Health Drug Benefits.* 2014;7(6):334-340. | Retrospective cohort HEOR analysis | Patients with narcolepsy, SWD, or OSA | N/A | Armodafinil and modafinil | - After therapy initiation, the armodafinil cohort had lower total all-cause medical costs compared with the modafinil cohort. - Armodafinil has a daily average consumption (DACON) of 1.04 compared with a DACON of 1.47 for modafinil. - Results support true once-daily dosing for armodafinil and provide real-world support for the pharmacokinetic data showing that on a milligram-to-milligram basis, armodafinil sustains higher plasma concentrations versus modafinil during a 24-h period. |
| Herring JW, et al. Alertness and psychomotor performance effects of the histamine-3 inverse agonist MK-0249 in obstructive sleep apnea patients on continuous positive airway pressure therapy with excessive daytime sleepiness: a randomized adaptive crossover study. *Sleep Med.* 2013;14(10): 955-963. | Double-blind, randomized, placebo- and active-controlled, 6-sequence crossover trial | Patients with OSA and EDS despite nCPAP therapy (N=125) | Clinic-based | MK-0249 | - Trial was terminated at the second interim analysis due to futility. - MK-0249 did not demonstrate statistical significance in its primary efficacy (mean MWT after 2 wk of treatment) compared to placebo, whereas modafinil demonstrated an improvement compared to placebo. - A higher percentage of patients reported AEs with MK-0249 (45.0%) compared to placebo (35.9%) or modafinil (34.2%), mainly due to the higher incidences of insomnia and anxiety associated with MK-0249. |
| Sun H, et al. Early-stage comparative effectiveness: randomized controlled trial with histamine inverse agonist mk-7288 in excessive daytime sleepiness patients. *J Clin Pharmacol.* 2013;53(12):1294-1302. | Double-blind, randomized, placebo- and active-controlled, 4-period crossover trial | Patients with OSA and EDS despite nCPAP therapy (N=56) | Clinic-based | MK-7288 | - MK-7288 improved MWT sleep latency and country vigilance driving standard deviation of lane position (SDLP) compared to placebo.   - Modafinil demonstrated greater improvement in mean MWT sleep latency than MK-7288.   - There was no difference between modafinil and MK-7288 on country vigilance driving SDLP. - The most frequently reported AEs in patients treated with MK-7288 were insomnia and headache. |
| Vernet C, et al. Residual sleepiness in obstructive sleep apnoea: phenotype and related symptoms. *Eur Respir J.* 2011;38(1):98-105. | N/A | Patients with OSA and EDS vs patients without EDS vs healthy patients (N=60) | Clinic-based | N/A | - Patients with RES tended to be more sleepy before CPAP and had lower stage N3 percentages, more periodic leg movements (without arousals), lower mean sleep latencies and longer daytime sleep durations after CPAP than patients without RES. - Numerous mechanisms of RES in OSA include insufficient CPAP adherence/titration, insufficient sleep syndrome, and coexisting sleep, psychiatric, and medical disorders. - RES in apneic patients differs markedly from sleepiness in central hypersomnia and the association between RES, periodic leg movements, apathy, and depressive mood parallels the post-hypoxic lesions in noradrenaline, dopamine, and serotonin systems in animals exposed to intermittent hypoxia. |

AE, adverse event; BFI, Brief Fatigue Inventory; BP, blood pressure; CGI-C, Clinical Global Impression of Change; CGI-I, Clinical Global Impression–Improvement; CI, confidence interval; CPAP, continuous positive airway pressure; CV, cardiovascular; CVA, cerebrovascular accident; DBP, diastolic blood pressure; EDS, excessive daytime sleepiness; ES, excessive sleepiness; ESS, Epworth Sleepiness Scale; fMRI, functional magnetic resonance imaging; FOSQ-10, Functional Outcomes of Sleep Questionnaire; HR, heart rate; HRQoL, health-related quality of life; KSS, Karolinska Sleepiness Scale; LS, least squares; min, minutes; MWT, Maintenance of Wakefulness Test; nCPAP, nasal CPAP; OLE, open-label extension; OSA, obstructive sleep apnea; OSLER, Oxford Sleep Resistance Test; PAP, positive airway pressure; PSG, polysomnography; PVT, psychomotor vigilance task; QoL, quality of life; QTcF, QT interval using Fridericia’s correction; RT, reaction time; SBP, systolic blood pressure; SD, standard deviation; SDLP, standard deviation of lane position; SF-36, 36-Item Short Form Health Survey; SSS, Stanford Sleepiness Scale; SWD, shift work disorder; TEAE, treatment-emergent adverse event.
